# Chimeric Antigen Receptor T Cells Targeting CD19 and GCC in Metastatic Colorectal Cancer: A Nonrandomized Clinical Trial

**DOI:** 10.1001/jamaoncol.2024.3891

**Published:** 2024-09-19

**Authors:** Naifei Chen, Chengfei Pu, Lingling Zhao, Wei Li, Chang Wang, Ruihong Zhu, Tingting Liang, Chao Niu, Xi Huang, Haiyang Tang, Yizhuo Wang, Hang Yang, Beibei Jia, Xianyang Jiang, Guiting Han, Wensheng Wang, Dongqi Chen, Yiming Wang, Eric K. Rowinsky, Eugene Kennedy, Victor X. Lu, Guozhen Cui, Zhao Wu, Lei Xiao, Jiuwei Cui

**Affiliations:** 1 Cancer Center, the First Hospital of Jilin University, Changchun, China; 2 Innovative Cellular Therapeutics Co, Ltd, Shanghai, China; 3 Innovative Cellular Therapeutics Inc, Rockville, Maryland; 4 80 Horseshoe Pt, Phoenixville, Pennsylvania

## Abstract

**Importance:**

Chimeric antigen receptor (CAR) T-cell therapy (CART) has transformed the treatment landscape of hematologic cancer, but has negligible effects for adult solid cancers. In this trial, an autologous CAR T-cell product demonstrated antitumor activity in heavily pretreated patients with metastatic colorectal cancer (mCRC).

**Objective:**

To evaluate the safety and efficacy of guanylate cyclase-C (GCC19) CART in participants with metastatic colorectal cancer (mCRC).

**Design, Setting, and Participants:**

This single-arm, nonrandomized, phase 1 trial was conducted at the First Hospital of Jilin University from December 3, 2020, to April 13, 2022. Data analysis was conducted from May 2022 to April 2024. Adults with relapsed and refractory mCRC expressing GCC were treated with GCC19CART, a mixture of autologous CAR T cells transduced with lentiviral vectors expressing genes that encode either CD-19 CAR or GCC CAR.

**Main Outcomes and Measures:**

Safety and tolerability of CAR T-cell therapy targeting GCC in patients with mCRC without therapeutic options is capable of conferring a reasonable likeliness of clinical benefit. Other outcomes included objective response rate, progression-free survival, overall survival, and immune activation.

**Results:**

Of 15 patients 9 (60%) were women, and the median (range) age was 44 (33-61) years. Treatment with GCC19CART was associated with the development of cytokine release syndrome and diarrhea in most patients, all of which were self-limited and manageable. The objective response rate was 40%, with a partial response in 2 of 8 and 4 of 7 patients treated with either 1 × 10^6^ cells/kg or 2 × 10^6^ cells/kg. Median overall survival was 22.8 months (95% CI, 13.4-26.1) at data cutoff; the median progress-free survival was 6.0 months in the high dose level group (95% CI, 3.0 to not available).

**Conclusions and Relevance:**

The results of this nonrandomized clinical trial suggest that GCC19CART was safe and tolerable in heavily pretreated patients with mCRC and is the first CAR T-cell therapy known to produce objective clinical activity in refractory cancer. Given the paucity of effective therapeutics developed for colorectal cancer in recent decades, the observation that CD-19 CART target engagement can robustly induce GCC19CART target engagement sufficient to produce objective activity may serve as a foundation to develop effective cellular therapy in mCRC and other solid cancers.

**Trial Registration:**

Chinese Clinical Trial Registry: ChiCTR2000040645

## Key Points

**Question:**

Can chimeric antigen receptor T-cell therapy (CART) change the therapeutic landscape of advanced colorectal and other solid cancers akin to its effects in the hematologic cancers?

**Findings:**

In this phase 1 nonrandomized clinical trial of 15 patients with metastatic colorectal cancer (mCRC), the safety and preliminary antitumor activity of guanylate cyclase-C (GCC19) CART, a novel chimeric antigen receptor technology, were evaluated. Its CD19CART component expanded on engaging CD19 T-cells, which activate GCCCART directed against mCRC that express GCC; an objective response occurred in 40% of patients with mCRC, and GCC19CART was well tolerated, with diarrhea, an on-target effect, as its principal toxic effect.

**Meaning:**

The results of this nonrandomized clinical trial indicate that CART is capable of anticancer activity in heavily pretreated mCRC by targeting a solid cancer antigen.

## Teaser

This nonrandomized clinical trial examines the safety and efficacy of guanylate cyclase-C (GCC) chimeric antigen receptor T-cell therapy in patients with metastatic colorectal cancer.

## Introduction

Colorectal cancer (CRC) is the second leading cause of cancer death worldwide, with approximately half of patients ultimately developing advanced, incurable CRC, and limited advancements in treatment outcomes.^1,2,3^ Chimeric antigen receptor (CAR) T-cell therapy (CART) has conferred marked efficacy in hematologic cancers but not in solid cancers.^4^ One contributing factor is the robust expansion of CD19-targeting CAR T cells compared with those targeting solid tumor antigens. We observed that when autologous transduced CD19 CAR T cells mixed with nontransduced T cells are administered after ex vivo expansion, both cell types rapidly proliferate (eResults in Supplement 1). Moreover, CD19 CART enhanced the proliferation of coinfused CAR T-cells targeting solid tumor antigens, like guanylyl cyclase C (GCC), which is expressed by 70% to 80% of CRC metastases. Its expression in normal tissue is limited to the apical surfaces of intestinal epithelial cells facing the lumen of the intestine, where it is isolated from systemic circulation by tight junctions; thus, it is inaccessible to T cells.^5,6,7^ Additionally, transduction of genes encoding for interferon γ (IFN-γ), interleukin (IL)–6, and IL-12 enhanced proliferation, trafficking, and infiltration of CD19 CAR T cells into malignant tumors.^8,9,10^

This CAR platform, known as CoupledCAR, consists of CAR T cells targeting a specific solid tumor antigen, such as GCC CAR T cells directed against GCC, and 3 subpopulations of CD19 CAR T cells, each engineered to express either IFN-γ, IL-6, or IL-12 in a single product (eFigure 1 in Supplement 1). This report describes the safety and preliminary anticancer activity of GCC19CART, which is a clinical candidate engineered using this approach, in what is to our knowledge the first phase 1 study in participants with mCRC.

## Methods

### Study Design and Treatment

**Figure 1.**
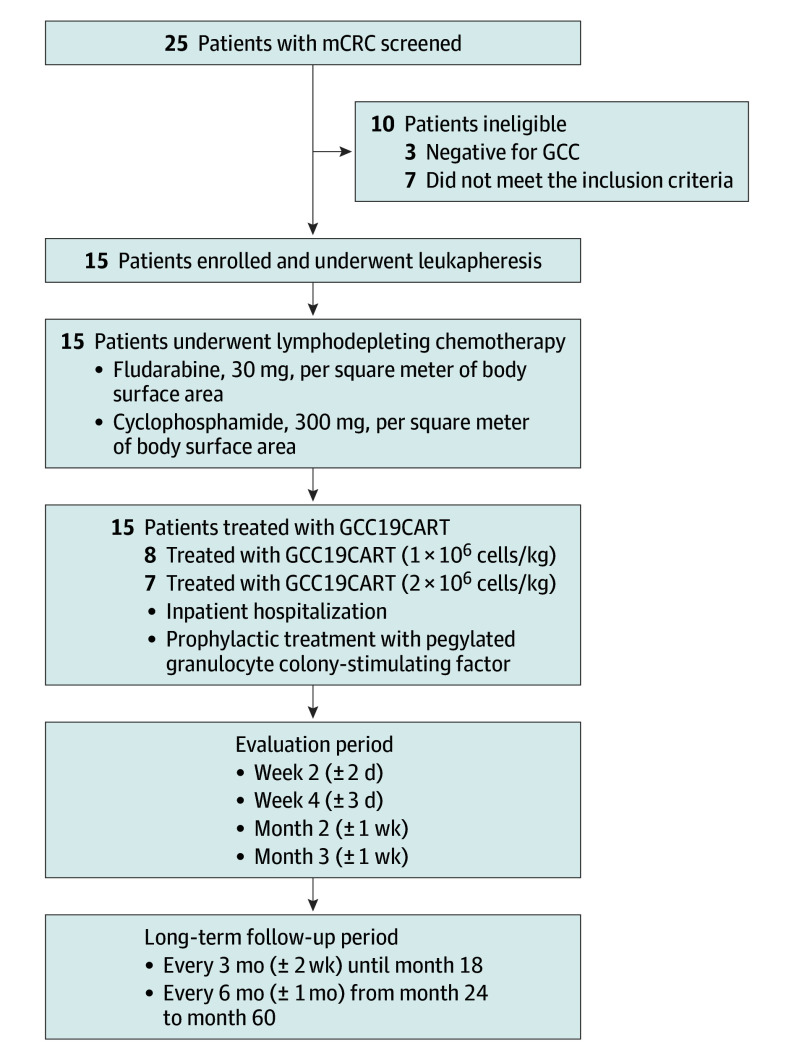
Trial Progress from Screening to Long-Term Follow-Up GCC indicates guanylate cyclase-C; GCC19CART, guanylate cyclase-C chimeric antigen receptor T-cell therapy; mCRC, metastatic colorectal cancer.

Patient eligibility and additional details are provided in the eMethods in Supplement 1. A single course of fludarabine, 30 mg/m^2^, and cyclophosphamide, 300 mg/m^2^, was administered for lymphodepletion 3 days before GCC19CART cells at 1 × 10^6^ or 2 × 10^6^ cells/kg (Figure 1). The protocol (Supplement 2) was approved by the institutional review board at the Medical Ethical Committee of First Hospital of Jilin University, and written informed consent was obtained from all patients. Data were locked on April 15, 2024. Full details regarding the manufacture of GCC19CART are provided in the eMethods in Supplement 1.

### Study End Points

The primary objective was to assess the adverse effects of GCC19CART, particularly dose-limiting toxic effects. Toxic effects were assessed by the Common Terminology Criteria for Adverse Events, version 4.0 (National Cancer Institute). The definition of dose-limiting toxic effects and discontinuation rules for dose escalation are provided in the eMethods in Supplement 1. Secondary objectives included the objective response rate (ORR) and progression-free survival (PFS) as assessed by an independent review committee and overall survival (OS). Metabolic response was assessed by the independent review committee using Positron Emission Tomography Response Criteria in Solid Tumors, version 1.0 (Radiological Society of North America).

### Statistical Analysis

Statistical analyses are described in the eMethods in the Supplement 1. All preclinical and clinical statistical analyses were conducted using GraphPad PRISM software, version 9.1.0.

## Results

### Study Population and Treatment

Fifteen patients with mCRC received GCC19CART from December 2020 to September 2021 following screening of 25 patients, of whom 10 were ineligible. The median age of the enrolled patients was 44 years (range, 33-61 years) (eTable 1 in Supplement 1). Fourteen patients had programmed cell death while receiving multiple lines of prior standard therapy (median, 3 lines [range, 2-6]), whereas 1 patient who was intolerant to initial treatment received only first-line therapy.

### Safety and Toxic Effects

Fourteen of 15 patients (93%) experienced at least 1 grade 3 or higher adverse event (AE) (Table). Fourteen participants (93%) developed manifestations of cytokine release syndrome (CRS), with 13 (87%) and 1 (8%) experiencing grades 1 and 2, respectively. The median onset of CRS was 4 days (range, 2-7) postinfusion, and the median duration was 4 days (range, 2-12). Twelve patients (80%) received tocilizumab to manage CRS, whereas 5 (33%) and 3 patients (20%) received corticosteroids and/or dasatinib, respectively. One patient experienced grade 4 neurotoxic effects that resolved within 30 days with high-dose corticosteroids.

**Table.** Adverse Events After GCC19CART Treatment for 15 Patients

Adverse event	No. of patients (%)					
	Dose level 1 (1 × 10^6^ cells/kg; n = 8)			Dose level 2 (2 × 10^6^ cells/kg; n = 7)		
	1-2	3	4	1-2	3	4
Leukopenia	5 (62.5)	2 (25.0)	0	2 (28.6)	4 (57.1)	0
Neutropenia	5 (62.5)	2 (25.0)	0	3 (42.9)	1 (14.3)	2 (28.6)
Lymphopenia	1 (12.5)	6 (75.0)	1 (12.5)	2 (28.6)	2 (28.6)	3 (42.9)
Thrombocytopenia	6 (75.0)	2 (25.0)	0	4 (57.1)	1 (14.3)	1 (14.3)
Fibrinogen level decreased	5 (62.5)	0	0	5 (71.4)	2 (28.6)	0
Alanine aminotransferase level increased	1 (12.5)	0	0	1 (14.3)	0	0
Aspartate aminotransferase level increased	5 (62.5)	0	0	6 (85.7)	0	0
GGT level increased	5 (62.5)	0	0	6 (85.7)	0	0
Alkaline phosphatase level increased	6 (75.0)	0	0	5 (71.4)	0	0
Hypoalbuminemia	8 (100.0)	0	0	7 (100.0)	0	0
Neurotoxic effects	0	0	0	0	0	1 (14.3)
Cytokine release syndrome	7 (87.5)	0	0	6 (85.7)	0	0
Infection	0	0	0	1 (14.3)	1 (14.3)	0
Diarrhea	3 (37.5)	4 (50.0)	0	3 (42.9)	4 (57.1)	0
Abdominal pain	3 (37.5)	0	0	1 (14.3)	0	0
Rash	3 (37.5)	0	0	0	0	0
Hypokalemia	3 (37.5)	0	0	5 (71.4)	1 (14.3)	0
Hyponatremia	3 (37.5)	1 (12.5)	0	6 (85.7)	1 (14.3)	0
Blood bilirubin level increased	3 (37.5)	0	0	4 (57.1)	0	0
Nausea	6 (75.0)	0	0	0	0	0
Abdominal distension	2 (25.0)	0	0	1 (14.3)	0	0
Creatinine level increased	3 (37.5)	0	0	3 (42.9)	0	0
Serum amylase level increased	2 (25.0)	0	0	1 (14.3)	0	0
Constipation	1 (12.5)	0	0	2 (28.6)	0	0
Cough	3 (37.5)	0	0	2 (28.6)	0	0
Blood lactate dehydrogenase level increased	8 (100.0)	0	0	7 (100.0)	0	0
Anemia	8 (100.0)	0	0	6 (85.7)	0	0
Hyperglycemia	6 (75.0)	1 (12.5)	0	4 (57.1)	1 (14.3)	0
Hypertriglyceridemia	7 (87.5)	1 (12.5)	0	5 (71.4)	2 (28.6)	0
Hypercholesterolemia	1 (12.5)	0	0	2 (28.6)	0	0
Chills	1 (12.5)	0	0	3 (42.9)	0	0

Abbreviations: GCC19CART, guanylate cyclase-C chimeric antigen receptor T-cell therapy; GGT, gamma-glutamyl transferase.

Diarrhea was observed in 14 patients (93%), with 6 and 8 experiencing grade 1 to 2 or 3 events, respectively. The median onset of diarrhea was 9 days (range, 5-14) and the median duration of grade 3 or higher diarrhea was 5 days (range, 1-13). Grade 3 diarrhea was treated with corticosteroids, dasatinib, and/or infliximab in addition to more conventional types of antidiarrheal and supportive care agents.^11,12,13^ Six patients were treated with corticosteroids (1-2 mg/kg of methylprednisolone or 5-10 mg of dexamethasone). Three patients received dasatinib, while 11 patients were treated with infliximab. Although diarrhea occasionally recurred, it completely resolved in all patients.

All patients had transient and reversible hematologic AEs, predominantly grade 1 to 2 (Table). These AEs typically resolved within 2 to 3 days. Lymphocytopenia, likely attributed to treatment effects, resolved within 3 months. There were no study-related deaths.

### Outcomes

**Figure 2.**
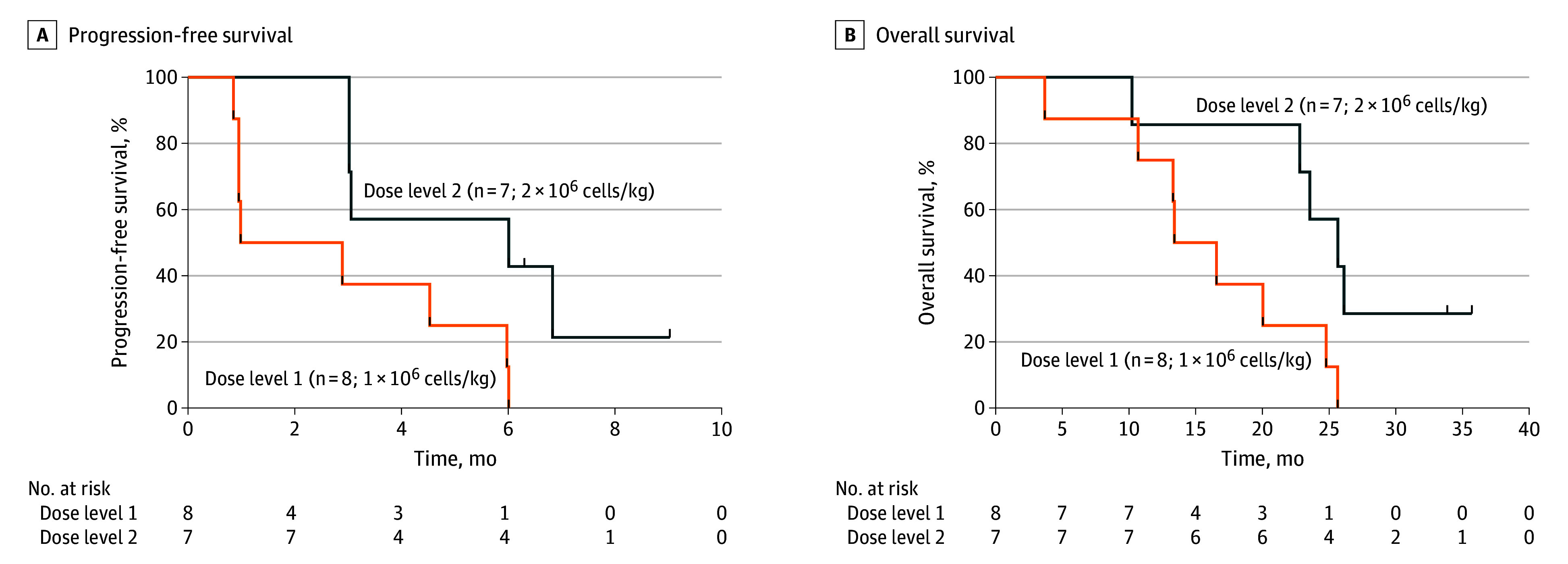
Clinical Response in 15 Heavily Pretreated Patients A, Kaplan-Meier analyses of progression-free survival in the dose level 1 (n = 8; 1 × 10^6^ cells/kg) and dose level 2 (n = 7; 2 × 10^6^ cells/kg) subsets of study participants. B, Kaplan-Meier analyses of overall survival in the dose level 1 (n = 8; 1 × 10^6^ cells/kg) and dose level 2 (n = 7; 2 × 10^6^ cells/kg) subsets of study participants.

The median PFS was significantly higher in patients treated with the higher dose than the lower dose (6.0 months [95% CI, 3.0 to not available] vs 1.9 months [95% CI, 1.0 to not available]; *P* = .03) (Figure 2A). Median overall survival was 22.8 months (95% CI, 13.4-26.1) at the data cutoff date, representing a median follow-up of 22.8 months (range, 3.7-35.7) (Figure 2B).

Of 15 patients, 6 (40%) experienced PRs, including 3 not confirmed (eFigure 13A in Supplement 1). The median duration of response was 5.1 months (range, 2.0-8.0). Five additional patients had stable disease as their best response, resulting in an overall clinical benefit rate of 73%.

Eleven of 15 patients (73%) had either a complete metabolic response (1 patient) or partial metabolic responses (10 patients), including 5 (63%) and 6 patients (86%) treated with GCC19CART at dose levels 1 and 2, respectively (eFigure 13B in Supplement 1). Pretreatment and posttreatment biopsy specimens of 1 patient were analyzed using single-cell RNA sequencing to characterize the nature of the CAR T cells in the tumor microenvironment(eFigures 14-15 in Supplement 1).

## Discussion

To our knowledge, this is the first time a cellular therapy consisting of autologous CAR T cells transduced to express a solid cancer antigen dependent on CD19 CAR T cells for expansion. Unlike hematologic cancers, GCC-targeting CAR T cells may cause self-limited diarrhea by binding to GCC in the intestinal lumen. In the present study, diarrhea was managed proactively with supportive care measures, as well as with infliximab, dasatinib, and corticosteroids. Severe manifestations of CRS were not observed despite engineering genes encoding for IFN-γ, IL-6, and IL12 into the GCC19CART product.

The clinical activity of GCC19CART is attributed to the CoupledCAR platform, which uses CD19 CAR T cells to expand GCCCART cells targeting CRC. The objective activity of GCC19CART suggests its potential effect in less heavily pretreated individuals or as consolidation/adjuvant therapy for high-risk recurrence. Despite the limited number of cases, the clinical data suggest a clear efficacy of GCC19CART in patients with advanced CRC with liver metastasis, as shown in eFigure 19 in Supplement 1.

Although complete responses were not noted, this study may be viewed as foundational for future investigations in CRC and other solid cancers, particularly in less advanced disease. This mechanistic approach in CoupledCAR may extend to other CARs and adoptive cellular immunotherapies, potentially offering promise for treating a broader spectrum of solid cancers.

### Limitations

This study was limited by the small sample size and brief durability of objective responses. However, these results may provide a hopeful foundation for CART in CRC and other solid cancers.

## Conclusions

This nonrandomized clinical trial demonstrated the favorable safety profile of GCC19CART and its significant clinical objective activity in patients with relapsed or refractory metastatic CRC after extensive prior treatment.

## Notes

Supplement 1. eResults. eMethods. eTable 1. Characteristics of Patients at Baseline (n = 15) eTable 2. Medical history of each patient prior to GCC19CART treatment eTable 3. CAR-T cell expansion post infusion for each patient eFigure 1. Schematic diagram of the GCC19CAR constructs used in this study eFigure 2. GCC CAR-T and CD19 CAR-T cell proliferation. eFigure 3. CAR-T cell phenotypes eFigure 4. CAR-T cell cytokines eFigure 5. CD19 CAR-T cells enhance the cytotoxicity of GCC CAR-T cells in vivo eFigure 6. GCC staining by immunohistochemistry of primary and metastatic colorectal carcinoma samples of the study patients eFigure 7. Characterization of each infusion product by quantitative polymerase chain reaction (qPCR) eFigure 8. Characterization of GCC19CART administered to each patient eFigure 9. CAR T-cell proliferation and cytokine release after GCC19CART treatment eFigure 10. CAR copy number in transduced T cells that express different transgenes in peripheral blood samples, as assessed by quantitative polymerase chain reaction (qPCR) eFigure 11. GCC CAR-T cell numbers, as assessed by both flow cytometry and qPCR in each of the fifteen study patients eFigure 12. CD19 CAR-T cell numbers as assessed by both flow cytometry and qPCR in the fifteen study patients eFigure 13. Clinical Response in 15 Heavy-Pretreated Patients. eFigure 14. Single-cell RNA-sequencing from colorectal cancer biopsies pre- and post-treatment with GCC19CART eFigure 15. Single cell clustering and downstream analysis eFigure 16. Imaging from patient 1 after CAR-T cells infusion eFigure 17. Imaging results from patient 4 eFigure 18. Imaging results from patient 9 eFigure 19. Response rate in patients after CAR-T cells infusion eReferences.

Supplement 2. Trial protocol

Supplement 3. Data sharing statement
