# Supplementary material for: Chimeric Antigen Receptor T Cells Targeting CD19 and GCC in Metastatic Colorectal Cancer: A Nonrandomized Clinical Trial
Source: JAMA Oncol. 2024 Sep 19;10(11):1532–6. doi: 10.1001/jamaoncol.2024.3891 (PMC11413756; doi:10.1001/jamaoncol.2024.3891)
Supplement: Supplement 1. — eResults. eMethods. eTable 1. Characteristics of Patients at Baseline (n = 15) eTable 2. Medical history of each patient prior to GCC19CART treatment eTable 3. CAR-T cell expansion post infusion for each patient eFigure 1. Schematic diagram of the GCC19CAR constructs used in this study eFigure 2. GCC CAR-T and CD19 CAR-T cell proliferation. eFigure 3. CAR-T cell phenotypes eFigure 4. CAR-T cell cytokines eFigure 5. CD19 CAR-T cells enhance the cytotoxicity of GCC CAR-T cells in vivo eFigure 6. GCC staining by immunohistochemistry of primary and metastatic colorectal carcinoma samples of the study patients eFigure 7. Characterization of each infusion product by quantitative polymerase chain reaction (qPCR) eFigure 8. Characterization of GCC19CART administered to each patient eFigure 9. CAR T-cell proliferation and cytokine release after GCC19CART treatment eFigure 10. CAR copy number in transduced T cells that express different transgenes in peripheral blood samples, as assessed by quantitative polymerase chain reaction (qPCR) eFigure 11. GCC CAR-T cell numbers, as assessed by both flow cytometry and qPCR in each of the fifteen study patients eFigure 12. CD19 CAR-T cell numbers as assessed by both flow cytometry and qPCR in the fifteen study patients eFigure 13. Clinical Response in 15 Heavy-Pretreated Patients. eFigure 14. Single-cell RNA-sequencing from colorectal cancer biopsies pre- and post-treatment with GCC19CART eFigure 15. Single cell clustering and downstream analysis eFigure 16. Imaging from patient 1 after CAR-T cells infusion eFigure 17. Imaging results from patient 4 eFigure 18. Imaging results from patient 9 eFigure 19. Response rate in patients after CAR-T cells infusion eReferences. [file jamaoncol-e243891-s001.pdf]

Supplemental Online Content

Chen N, Pu C, Zhao L, et al. Chimeric antigen receptor T cells targeting CD19 and GCC in metastatic colorectal cancer: a nonrandomized clinical trial. *JAMA Oncol*. Published online September 19, 2024. doi:10.1001/jamaoncol.2024.3891

eResults.
eMethods.
eTable 1. Characteristics of Patients at Baseline (n = 15)
eTable 2. Medical history of each patient prior to GCC19CART treatment
eTable 3. CAR-T cell expansion post infusion for each patient
eFigure 1. Schematic diagram of the GCC19CAR constructs used in this study
eFigure 2. GCC CAR-T and CD19 CAR-T cell proliferation.
eFigure 3. CAR-T cell phenotypes
eFigure 4. CAR-T cell cytokines
eFigure 5. CD19 CAR-T cells enhance the cytotoxicity of GCC CAR-T cells *in vivo*
eFigure 6. GCC staining by immunohistochemistry of primary and metastatic colorectal carcinoma samples of the study patients
eFigure 7. Characterization of each infusion product by quantitative polymerase chain reaction (qPCR)
eFigure 8. Characterization of GCC19CART administered to each patient
eFigure 9. CAR T-cell proliferation and cytokine release after GCC19CART treatment
eFigure 10. CAR copy number in transduced T cells that express different transgenes in peripheral blood samples, as assessed by quantitative polymerase chain reaction (qPCR)
eFigure 11. GCC CAR-T cell numbers, as assessed by both flow cytometry and qPCR in each of the fifteen study patients
eFigure 12. CD19 CAR-T cell numbers as assessed by both flow cytometry and qPCR in the fifteen study patients
eFigure 13. Clinical Response in 15 Heavy-Pretreated Patients.
eFigure 14. Single-cell RNA-sequencing from colorectal cancer biopsies pre- and post-treatment with GCC19CART
eFigure 15. Single cell clustering and downstream analysis
eFigure 16. Imaging from patient 1 after CAR-T cells infusion
eFigure 17. Imaging results from patient 4
eFigure 18. Imaging results from patient 9
eFigure 19. Response rate in patients after CAR-T cells infusion
eReferences.

This supplemental material has been provided by the authors to give readers additional information about their work.

eResults

Engineering GCC19CART

GCC19CART consists of purified T-cells engineered to express: i) chimeric antigen receptor (CAR) against human GCC; and ii) CAR targeting the human CD19 B-cell marker (CD19CART) in three separate constructs, each of which comprises one of three vector-encoded cytokine genes, interferon-γ (IFN-γ), interleukin-6 (IL-6), or interleukin-12 (IL-12). Both proteins are comprised of the extracellular structure of a single-chain antibody variable fragment (scFv) that recognizes human GCC or CD19, a CD8 transmembrane domain, a 4-1BB costimulatory domain, and a CD3ζ signaling domain. An NFAT-mediated promoter drives expression of the cytokines in the CD19CART constructs.

CD19 CAR-T cells with additional exogenous cytokine further activate GCC CAR-T cells in vitro

An aim of our studies was to determine if T-cell activation due to CD19 CAR-T combined with B cells promote GCC CAR-T cell proliferation.

As shown in eFigure 2, both GCC CAR-T cells and CD19 CAR-T cells in the test group (GCC CAR + CD19 cytokine CAR-T) and control group #2 (GCC CAR + CD19 CAR-T) are comprised of higher percentages of proliferating cells when co-cultured with B cells. This is supported by the results of studies using a cell proliferation assay.

GCC CAR-T and CD19 CAR-T cell pellets were analyzed for expression of the CAR-T cell activation markers CD25 and CD137, exhaustion marker PD-1, and effector/memory markers CD45RA and CD62L. Activation markers in the GCC CAR-T cell population were studied under several conditions, most notably in the presence or absence of CD19 CAR cells or B cells, as shown

© 2024 Chen N et al. *JAMA Oncology.*

in eFigures 3A and B. Higher expression of the T-cell activation markers was noted in both GCC-CART and CD19CART components of GCC19CART when co-cultured with B-cells, thereby validating this *in vitro* cell culture model. As shown in eFigure 3C, the percentages of GCC CAR and CD19 CAR T cells expressing exhaustion markers were also increased. Regarding the effector/memory T-cell markers CD45RA and CD62L, GCC-CART cells with CD19 CAR and CD19+ B cells had higher effector (CD45RA+ CD62L-) and effector memory (CD45RA- CD62L-) phenotypes, and lower central memory (CD45RA- CD62L+) phenotypes (eFigures 3D-F). There were no differences in activation status of GCC CAR-T and CD19 CAR-T between the test group (GCC19CART) and control group #2 (GCC CAR + CD19 CAR-T). However, significant differences were observed between: i) subgroups co-cultured with and without B cells ($P<0.05$), and ii) test group/control #2 and control #1 without CD19 CAR-T components ($P<0.05$). These results suggest that the CD19 CAR-T component of GCC19CART activated GCC CAR by increasing activation markers and effector memory T cell populations. Both CD19 CAR and CD19+ B cells were required to activate GCC CAR.

The mean concentration values of serum and intracellular cytokines from the six sub-groups are displayed in eFigure 4. Higher levels of both serum and intracellular cytokines were observed in both GCC CAR-T and CD19 CAR-T components when co-cultured with B cells than without B cells. The activation of GCC19CART in the presence of B cells increased cytokine (IL-6, IL-12, and IFN-γ) production *in vitro.* This was also the case for endogenous IL-2, GZMB, and TNF-α. Both CD19 CAR-T components of GCC19CART and B cells were likely responsible for these effects.

These studies demonstrated that the CD19 CAR-T components of GCC19CART increase the

© 2024 Chen N et al. *JAMA Oncology.*

activation and proliferation of both CD19 CAR-T and GCC CAR-T populations in the presence of CD19+ B cells, which, in turn, results in cytokine release. GCC-CART can be activated by the interactions between CD19 CAR-T and B cells, even in the absence of GCC positive cells, and activation can occur prior to cell trafficking into the tumor *in vivo*. Compared to the non-cytokine control group (control #2 GCC CAR+CD19 CAR-T without B cells), the GCC19CART (GCC CAR + CD19 cytokine CAR-T) group releases more cytokines and proliferates more rapidly, however, the magnitude of these differences is donor-dependent. The results of co-culturing studies, as well as studies performed in various animal models, support the proposed mechanism of action for GCC19CART.

CD19 CAR-T enhances killing by GCC CAR-T in vivo

To investigate if GCC19CART enhances the activity of GCC CAR-T against solid malignancies *in vivo*, a tumor model was established by implanting 5.0 x 10^6 T84 cells SC into NOG mice. Various combinations of CD19 CAR-T, GCC CAR-T, and B cells were administered via the tail vein 11 days after tumor cell implantations, which was followed by administration of B cells at four times, thereby mimicking the B cell regenerative environment in patients (eFigure 5A). CD19 CAR-T cells expanded rapidly upon encountering B cells in both the cytokine CD19 CAR-T cell + B cell and the GCC19CART cell + B cell groups (eFigure 5C). Compared to GCC19CART + B cell group, expansion of GCC CAR-T cells in the GCC19CART cell group was much lower post- infusion (eFigure 5D). The peripheral blood levels of IFN-γ and GZMB were also significantly increased in the GCC19CART cell + B cell group (eFigure 5B). The GCC19CART cell + B cell group demonstrated the greatest magnitude of cytotoxicity, completely eliminated the tumor, while the

© 2024 Chen N et al. *JAMA Oncology.*

other groups did not (eFigure 5E). In conclusion, the GCC19CART + B cell group was superior at CAR-T cell expansion and cytokine release than the other groups.

GCC expression in colorectal cancer tissue

Immunohistochemistry (IHC) was used to determine GCC in colorectal cancer tissues of candidates for the study. The colorectal cancer tissue samples from all enrolled patients, comprised of primary or metastatic lesions to liver or lung, stained either 2+ or 3+ positive for GCC. Representative IHC images are shown in eFigure 6A.

In vivo expansion of CAR-T cells

CAR vector copies were detected using quantitative PCR as early as 2 days post-infusion (eFigure 9A-C), with peak GCC CAR-T vector copy number detected at a median time of 10 days (range, 7 to 12) post-infusion (eFigure 9B). GCCCART cells were detected in all patients, with median peak expansion observed at 10 days (range, 8-11) using flow cytometry (eFigure 9E). Both expansion and persistence of GCC19CART, as assessed by copy number and cell number, were superior in patients treated at the higher dose (eFigure 9A, D).

Notable increases in circulating cytokines, suggesting immune activation, occurred in all patients (eFigure 9G-O). IL-6, INF-γ, and granzyme B levels increased immediately post-infusion and peaked in approximately 1 week. A second peak was noted 2 to 3 weeks later. At both dose levels, IL-6, INF-γ, and granzyme B levels fluctuated indiscriminately following GCC19CART treatment until around Day 30 (eFigure 9H, L, M).

© 2024 Chen N et al. *JAMA Oncology.*

Frequency of transduced T cells expressing different vectors and correlation between flow cytometry and qPCR for CAR-T cell number in 15 patients

To examine the relationship between the frequency of CAR-positive cells and the copy numbers of the four vectors in peripheral blood, qPCR was used to monitor the copy number of each vector. This analysis enabled an understanding of the relationship between the copy number of CAR and the frequency, as well as the dynamic changes of the copy numbers of the four vectors in the blood (eFigure 10). By integrating the frequency data obtained through flow cytometry and the copy number data obtained through qPCR, the frequency of CAR-positive cells was related to their corresponding copy numbers (eFigures 11-12).

Immune Activation

A 33-year-old woman underwent serial biopsies of lung metastases and blood sampled, pre-treatment and on day 13 post-infusion concurrent with maximal T-cell expansion. SD disease was her best response, with the sum of her target lesions decreasing by 25%. Single-cell RNA sequencing and unsupervised analyses conducted on the lung metastases revealed 19 different cell clusters from pre- and post-treatment tumor samples (eFigure 15). On day 13 post-infusion, CAR-T cells accounted for 32% and 30% of all T cells in her peripheral blood and lung metastases, respectively (eFigure 14A) with GCCCART cells accounting for 20% and 34% of CAR-T cells in blood and tumor tissue, respectively (eFigure 14B). There has been a reduction in the ratio of cancer cell subsets compared to the total cell population (eFigure 14C). Further, B cells in both tumor and blood samples decreased after treatment, with the transforming growth factor (TGF)-β expressing (TGFβ+) and regulatory B cell subpopulations decreasing most notably (eFigure 14D). Furthermore, CD80/CD86+ M1 macrophages increased and CD163/CD206+ M2 macrophages decreased post-

© 2024 Chen N et al. *JAMA Oncology.*

treatment (eFigure 14E), whereas FOXP3+ Treg cells decreased (eFigure 14F). Several identical T-cell clones were noted in lung metastases sampled before and after treatment. More than 75% of normal T-cell clones in the post-treatment lung metastasis were new compared to those in the pre-treatment sample (eFigure 14G-I).

Exploratory studies of paired biopsy

Using single-cell RNA-sequencing and unsupervised analyses, 19 different cell clusters were identified from patient biopsies before and after CAR T-cell treatment (eFigure 15A). Each cell cluster was annotated by canonical markers, including T cells (CD3D, CD3G), macrophages (C1QA), DCs (CD1C), NK cells (KLRF1, KLRD1), neutrophils (CD16B), B cells (CD19, CD79A), fibroblasts (DCN, COL6A1, MMP2), and cancer cells (KRT8, KRT18, KRT19, EPCAM).[1,2]

Evaluating expression of known pathways in macrophage populations in solid cancers using gene set enrichment analysis (GSEA) revealed strong enrichment of those involved with antigen processing and presentation, cell adhesion, and natural killer (NK) cell-mediated cytotoxicity (eFigure 15D).

Differentially expressed genes of normal T-cells were detected in cancer sampled pre- and GCC19CART treatment. Increased levels of mRNA representing cell cycle- and cytotoxicity-associated signature genes were observed post-treatment, while exhaustion- and memory-associated genes were preferentially expressed in normal T-cells pre-treatment, suggesting that non-CAR T-cells were activated following GCCCART treatment (eFigure 15E). In NK cells in tumor sampled post-treatment, mRNA levels representing expression of JAK-STAT, cell adhesion and cytotoxicity-associated genes were noted, whereas TGFβ genes were preferentially expressed in NK cells before

© 2024 Chen N et al. *JAMA Oncology.*

treatment, suggesting that NK cells in cancer samples were also activated by GCC19CART (eFigure 15F). These results suggest the NK cells were activated after the infusion of GCC19CART.

© 2024 Chen N et al. *JAMA Oncology.*

eMethods

Lentiviral Vector Manufacturing

For lentiviral vectors manufacturing, suspension-adapted LV-MAX cells（Thermo Fisher, A35347) were grown in shaker flasks, transiently transfected with the transgene plasmid construct and three helper plasmids (third-generation lentiviral vector system).[3] Approximately 48 hours (h) after transfection, the vector containing supernatant was harvested, clarified, treated with benzonase, and concentrated. The concentrated material was diafiltered using hollow fiber tangential flow filtration to formulate the final vector product in T -cell compatible TexMacs media (Miltenyi Biotec 170-076-306) before freezing. [4]

CAR T-Cell Manufacturing

For GCC19CART manufacturing, peripheral blood mononuclear cells (PBMCs) were collected by apheresis and separated using CliniMACS Prodigy (Miltenyi Biotec 200-075-301). CD4+ and CD8+ T-cells were purified by CD4 (Miltenyi Biotec 200-070-132) and CD8 microbeads (Miltenyi Biotec 200-070-115), suspended in TexMacs media (Miltenyi Biotec 170-076-306) containing 12.5 ng/mL rhIL-2 (Miltenyi Biotec 170-076-147), and then stimulated with anti-CD3 and anti-CD28 microbeads (Miltenyi Biotec 130-111-160 or 170-076-156) followed by cultivation in 5% CO2 at 37°C for 24 h. T-cells were simultaneously transduced with a mixture of the four vectors encoding GCC-CD8tm-41BB-CD3ζ (GCCCAR), IFN-γ-CD19-CD8tm-41BB-CD3ζ (CD19CAR+IFN-γ), IL-6-CD19-CD8tm-41BB-CD3ζ (CD19CAR+IL-6), and IL-12-CD19-CD8tm-41BB-CD3ζ (CD19CAR+IL-12). The MOI values for transduction of target cells were 30, 10, 1, and 1, respectively.

© 2024 Chen N et al. *JAMA Oncology.*

The culture media was replaced after 24 h. Seven days after cell separation, the cells were washed, harvested, and resuspended in cryopreservation solution containing 7.5% DMSO, 25% 0.2 g/ml human serum albumin (HSA), 33.75% multiple electrolyte injection, and 33.75% dextran 40.

Flow Cytometry

T-cell marker panel

No more than 1 x 10^6 cells were washed with DPBS and centrifuged at 4℃. The resultant cell pellet was resuspended in stain buffer (BD 554656) to a concentration of 0.5 x 10^6 cells/100 µL. Next, both GCC (Acro GUC-H52H5) and CD19 (Acro CD9-H8259*)* in stain buffer were added. The cells were incubated at 2 to 8℃ in darkness for 30 min and then washed with DPBS. For the secondary antibody staining, a mixture of antibody containing anti-His tag AF488 (R&D IC0501G), PE streptavidin (BD 554061), hCD3 BV786 (BD 563800), hCD8-APC7 (Biolegend 344714), CD62L PE-CY7 (Biolegend 304822) was prepared and 100 µL was added to the cell pellet and incubated at 2 to 8℃ in darkness for 30 minutes. The cells were then centrifuged and resuspended in 100 µL DPBS. Prior to flow cytometry, DAPI BV421 (Thermo D21490) was added to the mixture. Gating positive population of GCC CAR-T and CD19 CAR-T from CD3+population. An analysis of all activation, memory, exhaustion markers from GCC CAR-T and CD19 CAR-T population indicated the presence of central memory T cells (CD45RA+CD62L+), effector memory T cell (CD45RA-CD62L-), and effector T cell (CD45RA+CD62L-).

CFSE panel

No more than 1 x 10^6 cells were washed with DPBS, centrifuged at 4℃,and the resultant cell pellet was resuspended in stain buffer to a concentration of 0.5 x 10^6cells per 100 µL, and diluted GCC

© 2024 Chen N et al. *JAMA Oncology.*

protein (Acro GUC-H52H5) and CD19 protein (Acro CD9-H8259*)* in stain buffer. The cells were then incubated at 2 to 8°C for 30 min in darkness, and washed with DPBS. The second staining antibody mixture, containing Anti-His tag AF488 (R&D IC0501G), PE streptavidin (BD 554061), hCD3 BV786 (BD 563800), and hCD8-APC7 (Biolegend 344714), was prepared in stain buffer, and 100 µL was added to the cell pellet and incubated at 2 to 8°C for 30 min in darkness. Next, the cells were centrifuged and resuspended in 100 µL DPBS. Gating positive population of GCC CAR-T and CD19 CAR-T from CD3+CD8+ population or CD3+CD4+ population. Gating Violet positive population (Cell Proliferation Dye eFluor450, eBioscience, 65-0842-85) of BV421 channel from GCC CAR-T or CD19 CAR-T population.

Vector copy numbers (VCN) Assay

This qPCR test was designed to quantify vector copy numbers in the transduced cells of GCC19CART or blood samples. Four sets of specific primers and probes were designed to distinguish among the viral vectors, each with its own standard curve. The details are shown below.

Reference Standard: p6701, p6213, p6239, p6277, 10^7 copy/µL.

Calibration Curve: 10 to 10^7 copies/5µL of each reference standard

Non-Target-Control (NTC): PCR-grade water

Sample Preparation: DNA was isolated with a Qiagen DNA preparation kit (Qiagen 51104, 51106). The DNA concentration was determined using a Qubit and the GCC19CART samples need further adjusted to 40 ng/µL in PCR-grade water.

qPCR Reaction: For each reaction mix 0.4 µL 10 mM forward/ reverse primer, 0.1 µL 10 mM probe (Thermo 431603401), 10 µL 2x Master mix (Thermo 4440038), 5 µL sample or standard solution

© 2024 Chen N et al. *JAMA Oncology.*

or NTC and 4.1 µL PCR-water (Thermo R0581). qPCR program: 50°C 2min; 95°C 10min; 40 cycles of 95°C 15s and 60°C 60s.

Assay Validity: 1) linear regression R^2 > 0.99; 2) CT value of lowest reference standard concentration < CT value of NTC

Copy Number Calculations: The results are reported as each VCN per 200 ng gDNA equivalent to 30000 diploid cells. Thus, the copy number of each vector is calculated as follows:

Copy per cell = CAR copies per 200 ng gDNA/30000.

VCN (Anti-GCC-ScFv) = Anti-GCC-ScFv copy per cell/GCC-CCAR%

VCN (Anti-CD19-ScFv) = [IFN-γ+IL-6 + IL-12] copy per cell/CD19CAR%

Single Cell mRNA Sequencing to Characterize GCC19CART Composition

Single-cell Dissociation

Single-cell RNA-seq studies were performed using NovelBio Bio-Pharm Technology Co.,Ltd. Tissue samples were surgically removed and stored in MACS Tissue Storage Solution (Miltenyi Biotec). The samples were then washed with PBS, minced into approximately 1mm^3 pieces on ice, enzymatically digested with 200 U/mL collagenase I (Worthington, LS004194), 100 U/mL collagenase IV (Worthington, LS004186) and 30 U/mL DNase I (Worthington, LS006330) for 20 min at 37°C with agitation. Next, samples were sieved through a 70μm cell strainer and centrifuged at 300g for 5 min. After removal of the supernatant, the pelleted cells were suspended in red blood cell lysis buffer (Miltenyi Biotec). Peripheral blood was also treated with red blood cell lysis buffer (Miltenyi Biotec, 130-094-183). After washing with PBS containing 0.04% BSA, the cell pellets were resuspended in PBS containing 0.04% BSA and re-filtered through a 35μm cell strainer. The

© 2024 Chen N et al. *JAMA Oncology.*

single-cells suspension was then stained with AO/PI for viability assessment using Countstar Fluorescence Cell Analyzer (???details).

Single-cell Sequencing

scRNA-Seq and V(D)J libraries were generated using a 10X Genomics Chromium Controller Instrument and Chromium Next GEM Single Cell 5' Reagent Kits v1.1, 16 rxns PN-1000165t, along with Chromium Single Cell V(D)J Enrichment Kit, Human T Cell, 96 rxns PN-1000005 according to the manufacturer's recommendations. Briefly, approximately 1000 cells/uL were loaded into each channel to generate single-cell Gel Bead-In-Emulsions (GEMs). Full-length cDNA was synthesized and barcoded within each GEM. Subsequently, GEMs were broken, and cDNA was pooled. Following cleanup, cDNA was amplified by PCR to construct both 5' gene expression and TCR-enriched libraries. The amplified product was fragmented prior to end-repair, A-tailing, and adaptor ligation. The final libraries were quantified using the Qubit High Sensitivity DNA assay (Thermo Fisher Scientific) and the size distribution of the libraries were determined using a High Sensitivity DNA chip on a Bioanalyzer 2200 (Agilent). All libraries were sequenced by an Illumina sequencer (Illumina, San Diego, CA) on a 150 bp paired-end run.

Single-cell RNA-Seq Data Processing

The second-generation high-throughput sequencing data were aligned and quantified using the Cell Ranger Single-Cell Software Suite (version 3.0.2, 10x Genomics) against the GRCh38 human reference genome. Unique molecular identifier (UMI) counts were summarized for each gene of each cell and converted into a Seurat object by the R package Seurat (version 4.0). Quality of cells were then assessed based on three metrics step-by-step: (1) The number of total UMI counts per cell

© 2024 Chen N et al. *JAMA Oncology.*

(200-4000); (2) The number of genes detected per cell (1,600-25,000); and (3) The proportion of mitochondrial gene counts (<25%). After excluding low-quality cells, 21,396 protein-coding genes across 12,245 single cells were available for downstream processing.

TCR analysis

The TCR-seq results were processed using Cell Ranger (version 3.0.2) against the human VDJ reference genome. In all TCR contigs assembled, if at least two cells had identical alpha–beta pairs, the alpha–beta pair were identified as clonal TCRs. To integrate TCR and gene expression results, the TCR-based analysis was performed only for cells identified as T cells.

Identification of Cell Types and Subtypes by Dimensional Reduction

After quality control assessments, raw UMI counts were log-normalized using a 10,000 scale. To cluster single cells by their expression, an unsupervised graph-based clustering algorithm implemented in Seurat v4 (version 4.0) was used. Single cells of four samples from each patient were integrated and embedded into a shared low-dimension space through integrated analysis (CCA) using Seurat v3 function IntegrateData. Genes with high variability were identified fitting the mean-variance relationship. Principal component analysis (PCA) was performed on approximately 2000 variable genes and the function FindClusters was used on 30 PCs with a resolution of 0.6 to perform the unsupervised clustering. Each cell cluster was annotated by the exceptionally high expression of canonical marker genes. For visualization, the dataset dimensionality was reduced using the Barnes-Hut t-Distributed Stochastic Neighbor Embedding (t-SNE).

© 2024 Chen N et al. *JAMA Oncology.*

Cytokine Release (CBA assay)

IL-2, IL-6, IFN-γ, IL-12, GZMB and TNF-α concentrations were measured using human cytokine kit (IL-2 (BD 558270), IL-6 (BD 558276), IFN-γ (BD 560111), IL-12 (BD 558283), GZMB (BD 560304) and TNF-α (BD 558273)).

Briefly, standard curves of the six cytokines ranging from 9.8 to 2500 pg/ml were prepared with standard samples provided in the kits. Cytokine analyses were performed using flow cytometry following a standardized protocol. Capture beads specific to each cytokine were mixed, and subsequently, the capture bead mixture and PE-labeled detection antibody were sequentially added to each sample, including the standard samples. The samples were then incubated for 3 hours to allow for optimal binding between the capture beads and cytokines. After incubation, centrifugation was performed, and the supernatant was carefully discarded. The resulting bead-cytokine complexes were resuspended in a wash buffer to remove any residual contaminants. The samples were then subjected to flow cytometry, wherein the cytokines were gated differently based on their fluorescence signal. To quantify the cytokine concentrations, calibration curves were generated using standard samples of known cytokine concentrations, and the fluorescence signals were compared to these curves using FCAP Array Software. Finally, the cytokine concentrations were normalized to pg/CART cells, enabling standardized comparisons across different samples.

Intracellular Cytokine Staining

Intracellular cytokine staining used a standard flow cytometry protocol. Briefly, cells were treated with BD GolgiPlug (BD 555029) at a concentration of 1 μl per ml of cell culture (approximately 1 x 10^6 cells per mL) and incubated for 4 h at 37°C with 5% CO^2. Following incubation, cells were

© 2024 Chen N et al. *JAMA Oncology.*

harvested by centrifugation and washed with ice-cold DPBS. The cell pellet was resuspended in staining buffer to achieve a final concentration of 0.5 x 10^6 cells per 100 µL. For surface staining, cells were incubated with the appropriate antibodies, including GCC (Acro GUC-H52H5) and CD19 (Acro CD9-H8259) proteins, diluted in staining buffer, for 30 min at 2 to 8°C in darkness. Subsequently, cells were washed twice with DPBS and incubated with a secondary staining mixture consisting of Anti-His tag AF488 (R&D IC0501G), PE streptavidin (BD 554061), hCD3 BV786 (BD 563800), and hCD8-APC7 (Biolegend 344714) for 30 minutes at 2 to 8°C in darkness. Following two additional washes with DPBS, the supernatant was discarded. To enable intracellular staining, cells were fixed and permeabilized using a commercially available fixation/permeabilization solution, incubating the cell pellet with 500 µL of the solution at 4°C for 30 minutes. After fixation, cells were washed twice with 1 ml of 1 x BD Perm/WashTM Buffer. Next, cells were resuspended in a mixture containing intracellular IFN-γ BV650 (BD 563416) and intracellular GZMB BV421 (BD 561151) antibodies diluted in 1 x BD Perm/WashTM Buffer. The cells were incubated for 30 minutes at 2 to 8°C in darkness. Following staining, the cells were washed twice with 1 ml of 1 x BD Perm/WashTM Buffer and finally resuspended in the same buffer for subsequent analysis. The intracellular staining aimed to detect IFN-γ and GZMB in the GCC CAR-T and CD19 CAR-T components. Flow cytometry analysis was performed using a suitable flow cytometer equipped with the requisite lasers and detectors to measure the fluorescence signals from the stained cells.

Cell Lines

The human chronic myelogenous leukemia cell line K562 [CCL-243], a GCC-positive colon tumor

© 2024 Chen N et al. *JAMA Oncology.*

cell line T84 [CCL-248] from a lung cancer metastasis, and a human GCC-negative embryonic kidney cell line HEK 293T [CRL-3216] were purchased from ATCC. The K562 cell line was maintained in RPMI 1640 (Gibco, 22400071) media containing 10% (v/v) fetal bovine serum (FBS). The T84 cell line was maintained in Dulbecco's Modified Eagle Medium/Nutrient Mixture F-12 (Gibco, 12400024) containing 10% (v/v) FBS. The HEK 293T cell line was maintained in Dulbecco's Modified Eagle Medium (Gibco, 11995040) containing 10% (v/v) FBS. The RK562 cell line is derived from K562 by transduction with a lentiviral vector expressing mCherry, a red fluorescent protein. Transduced K562 cells were single colony-selected and expanded to create the RK562 master cell bank (MCB). The RK562 MCB was qualified through flow cytometry analysis (positive for mCherry and negative for CD19), sterility testing, and mycoplasma testing. The RK19 cell line is derived from K562 by transduction with a lentiviral vector co-expressing mCherry and CD19 protein. This lentiviral vector confers both red fluorescence and CD19 expression. Transduced K562 cells were single colony selected and expanded to generate the RK19 MCB. The RK19 MCB was qualified through flow cytometry (positive for both mCherry and CD19), sterility testing, and mycoplasma testing.

In Vitro Study

At least 5 x 10^7 T cells were isolated from the PBMCs of three health donors using CD4/CD8 beads (Miltenyi Biotec 130-097-048 and 130-097-057) according to the vendor's standard protocol. Activated T-cells were isolated with TransAct (Miltenyi Biotec 130-111-160). After T-cell isolation, CD3, CD4, CD8 expression was assessed using flow cytometry. T cells were prepared for manufacturing GCC19CAR-T cells and control groups, including Control Group #1 (GCC CAR-T

© 2024 Chen N et al. *JAMA Oncology.*

cells without CD19 CAR and cytokines) and Control Group #2 (GCC CAR-T cells with CD19 CAR but without co-expression of the cytokines) were activated with TransAct. After 24 hours, the CAR-T cells were resuspended in TexMACS (Miltenyi Biotec 170-076-306) medium at a concentration of 5 x 10^6 cells per ml for flow cytometry analysis to confirm the absence of baseline activation and assessed by measuring CD25 and CD137 in the CAR-positive population. PBMCs (80 to100 mL) were collected from the same donor, and B cells were isolated using a pan B-cell isolation kit (Miltenyi Biotec 130-091-151). The transduced cells were divided into six sub-groups and co-cultured with or without B cells in TexMACS medium. This allowed for the evaluation of CAR-T cell biomarkers and intracellular cytokine release. Cell proliferation was assessed by labeling T cells with Violet-BV450 (Thermo 65-0842-85) dye and measuring the signal intensity after 96 hours of coculture. Supernatants from the coculture experiments were collected for cytokine analysis using a human cytokine kit (Bio-Rad). T-cell activation levels and efficacy were compared between the transduced cell groups in the presence or absence of B-cells. Additionally, the impact of the CoupledCAR approach on the anti-solid tumor efficacy of GCC CAR-T cells was investigated by co-culturing T84 cells with different combinations of NT cells, CD19 CAR-T cells, GCC CAR-T cells, and B-cells at 10:1 ratios, and luciferase activity was measured after 96 h of co-culture.

In Vivo Antitumor Studies

Animal studies were conducted in Shanghai Branch of Beijing Vital River Laboratory Animal Technologies Co. Ltd. using protocols compliant with the Institutional Animal Care and Use Committee (IACUC) of Shanghai Branch of Beijing Vital River Laboratory Animal Technologies Co. Ltd. A representative GCC expressing human colorectal carcinoma, T84, which was derived

© 2024 Chen N et al. *JAMA Oncology.*

from a lung metastasis, was obtained from ATCC (CCL-248) and implanted SC into female NOG mice.[5] When the tumor size reached 100 mm^3, the mice were randomly divided into the following groups: MOCK group (n = 4); NT group (n = 4); B cell group (n = 4); GCC19CART cell group (n = 4); GCC19CART cell plus B cell group (n = 4); cytokine CD19 CAR-T cell group (n = 4); and cytokine CD19 CAR-T cell plus B cell group (n = 4). B cells and test articles were administered to each group. Since the study mice did not have human B cells that are required for GCC19CART immune activity, they were periodically administered human B cells. To prevent graft-versus-host disease (GVHD), the human B cells and test articles/controls administered to each mouse came from the same donor. A B cell count of 6×10^6 was sufficient for complete depletion by CD19 CAR-T cells. Tumor volumes were calculated using the formula $V = \pi / 6 \times (\text{length} \times \text{width}^2)$. Peripheral blood was collected on days 7, 11, 14, and 17 to evaluate CAR-T cell expansion. Mice were treated with media, non-transduced T cells, low-dose GCC19CART cells, or high-dose GCC19CART cells, and were euthanized on day 29 following T-cell infusion. Various tissues were collected, fixed in formalin, embedded in paraffin, and stained with H&E. Animals were euthanized once tumors reached over 2000 mm^3.

Immunohistochemistry (IHC)

A rabbit polyclonal to GCC (Abcam (ab122404, 0.2 mg/mL) was qualified to be suitable for and used for IHC. The recombinant fragment corresponds to human GCC amino acid 982-1067 (EVRGETYLKGRGNETTYWLTGMKDQKFNLPTPPTVENQQRLQAEFSDMIANSLQKRQA AGIRSQKPRRVASYKKGTLEYLQLNTTD). After tumor sampling from study patients, 4-5 µm sections were de-waxed using xylene, washed with alcohol, and retained in water. A citric acid

© 2024 Chen N et al. *JAMA Oncology.*

antigen repair system was used for antigen retrieval. Next, the tissue sample and anti-GCC antibody (1 μg/mL) were incubated overnight, which was followed by incubation in biotin peroxidase detection system (CST, Inc., 8059S). GCC IHC staining was assessed using diaminobenzidine, and sections were counterstained with hematoxylin and eosin. Appropriate positive and negative control samples of both normal colorectal tissue and GCC-expressing colorectal cell lines were assayed concurrently (data not shown).

Study Design

Patient Eligibility

Eligibility included an Eastern Cooperative Oncology Group (ECOG) performance status of 0 to 1; histologically confirmed mCRC with ≥ 70% of cells expressing GCC. (eFigure 6); measurable by Response Evaluation Criteria in Solid Tumors Version1.1(RECIST); and at least two prior treatments for advanced disease, including oxaliplatin- and irinotecan-based regimens, and cetuximab if KRAS wild-type.

Definition of Dose-Limiting Toxicity

Dose-limiting toxicity (DLT) was defined as the following GCC19CART-related events that occurred in the 28-day period following GCC19CART treatment:

- Death
- Grade 3 neurotoxicity that does not resolve to ≤ grade 2 within 7 days or does not resolve to its pretreatment grade within 28 days.
- Grade 3 cytokine release syndrome (CRS) that does not resolve to ≤ grade 2 within 7 days.
- Any grade 4 CRS.
- Any grade 4 neurotoxicity.

- Any grade 3 seizure that does not resolve to ≤ grade 2 within 3 days.
- Any increase in aspartate aminotransferase (AST) or alanine aminotransferase (ALT) > 5 times the upper limit of normal (ULN) with a concurrent increase in total bilirubin > 3 times the ULN, which is not associated with CRS.
- Any new nonhematological AE of ≥ grade 3 or higher, which occurs within 28 day of treatment and fails to resolve to grade 2 or less within 14 days despite appropriate supportive measures.
- Infusion reactions of ≥ grade 2 lasting longer than 24 hours.
- Grade 4 neutropenia lasting longer than 21 days.
- Grade 4 thrombocytopenia lasting longer than 35 days.
- Any unexpected grade ≥ 3 event considered a DLT by the IRB.

Enrollment Stopping Rules

Patient enrollment was to be stopped for any of the following:

- ≥10% of patients experienced grade 5 CAR-T-related AEs within 30 days.
- The incidence of grade 4 CAR-T-related adverse events, including neurotoxicity, CRS, infection, and other nonhematological toxicities, lasting for 7 days was higher than 33%,

Statistical Analysis

The results are represented by mean values ± standard deviation of technical replicates or mean values ± standard error of biological replicates. The statistical methods for each experiment are described in corresponding figure legends. An unpaired two-tailed Student’s t-test was used to compare the results of two groups, whereas a one-way analysis of variance (ANOVA) was used to

compare more than two groups. A two-way ANOVA was used when comparing both time and treatments, and, if significant ($P < 0.05$), multiple comparisons were performed. All pre-clinical and clinical statistical analyses were performed using GraphPad PRISM 9.1.0. Notations for statistical significance include: ns, not significant; *, $P < 0.05$; **, $P < 0.01$; ***$P < 0.001$; and ****, $P < 0.0001$. Formal statistical methods were not used to determine sample size for animal and other preclinical studies. The results of single-cell RNA sequencing studies were analyzed using R software (R Foundation for Statistical Computing).[6] Gene set enrichment analysis (GSEA) was performed for the most significant kegg pathway genes.[7] The Kaplan-Meier (KM) method calculated the median PFS (mPFS) and median overall survival (mOS), as well as 95% confidence intervals (CI). The significance of the differences between survival curves was assessed using a log-rank test based on the Mantel-Cox method.

© 2024 Chen N et al. *JAMA Oncology.*

eTable 1. Characteristics of Patients at Baseline (n = 15) [a]

	No.(%)		
**Characteristic**	**Total (n=15)**	**Dose level 1 (1x10^6 cells/kg, n=8)**	**Dose level 2 (2x10^6 cells/kg, n=7)**
Age, mean(SD),y	43.1 (8.4)	43.8 (8.5)	42.3 (9.5)
Sex			
Female	9 (60.0)	5 (62.5)	4 (57.1)
Male	6 (40.0)	3 (37.5)	3 (42.9)
ECOG PS			
0	6 (40.0)	2 (25.0)	4 (57.1)
1	9 (60.0)	6 (75.0)	3 (42.9)
Location of primary lesion			
Colon	7 (46.7)	4 (50.0)	3 (42.9)
Rectum	8 (53.3)	4 (50.0)	4 (57.1)
Number of metastatic sites			
1	1 (6.7)	0	1 (14.3)
2	10 (66.7)	4 (50.0)	6 (85.7)
⩾3	4 (26.6)	4 (50.0)	0
Liver metastasis			
Yes	8 (53.3)	5 (62.5)	3 (42.9)
No	7 (46.7)	3 (37.5)	4 (57.1)
Number of prior therapy lines			
1	1 (6.7)[b]	0	1 (14.3)
2	5 (33.3)	2 (25.0)	3 (42.9)
⩾3	9 (60.0)	6 (75.0)	3 (42.9)
KRAS mutation			
Yes	9 (60.0)	5 (62.5)	4 (57.1)
No	6 (40.0)	3 (37.5)	3 (42.9)

[a]Baseline was the time of screening, before the infusion of GCC19CART.

[b]Subject did not tolerate standard chemotherapy and received unorthodox, albeit unknown, therapy.

© 2024 Chen N et al. *JAMA Oncology.*

eTable 2. Medical history of each patient prior to GCC19CART treatment

Patient Number	Date	Relevant Medical history	Efficacy/safety
**Patient 01**	January 2018	Diagnosed with locally advanced, CRC	
	January 2018	Radical resection via laparoscopy	
	February 2018	Treatment with seven courses of XELOX chemotherapy	
	May 2019	Resection of intraperitoneal metastases	
	May 2019	Treatment with three courses intraperitoneal hyperthermic perfusion of lobaplatin chemotherapy	Disease progression
	August 2019	Treatment with six courses of FOLFIRI chemotherapy and bevacizumab	Stable disease
	December 2019	Treatment with six courses of capecitabine chemotherapy and bevacizumab	Disease progression
	August 2020	Treatment with two courses of capecitabine chemotherapy and bevacizumab	Disease progression
	October 2020	Treatment with two courses of raltitrexed chemotherapy and bevacizumab	Disease progression
	December 2020	Informed consent for GCC19CART study obtained	
	December 2020	Leukapheresis	
	January 2021	GCC19CART treatment	
**Patient 02**	November 2019	Diagnosed with mCRC	
	December 2019	Resection of primary colorectal lesion.	Disease progression
	January 2020	Treatment with six courses of XELOX chemotherapy and bevacizumab for mCRC.	Stable disease
	September 2020	Treatment with three courses of FOLFIRI chemotherapy and bevacizumab. Discontinued because of intolerance	Intolerance
	October 2020	Palliative radiation therapy	Symptomatic Improvement
	December 2020	Treatment with one course of futibatinib, tegafur, gemcitabine and oxylatyne followed by disease progression.	Disease progression
	January 2021	Informed consent for GCC19CART study obtained	
	February 2021	Leukapheresis	
	March 2021	GCC19CART treatment	
**Patient 03**	September 2019	Diagnosed with mCRC	
	September 2019	Laparoscopic resection of primary rectal cancer lesion	
	September 2019	Treatment with eight courses of CAPOX chemotherapy followed by disease progression.	Disease progression
	October 2020	Liver recurrence resected	
	November 2020	Treatment with five courses of FOLFIRI chemotherapy and bevacizumab followed by disease progression	Disease progression
	March 2021	Informed consent for GCC19CART study obtained	
	March 2021	Leukapheresis	
	April 2021	GCC9CART treatment	
**Patient 04**	October 2018	Diagnosed with locally advanced CRC	
	October 2018	Resection of primary rectal lesion	
	November 2018	Treatment with traditional Chinese medicine	Disease progression
		Treated with various oxaliplatin- and irinotecan-based regimens after mCRC recurrence with treatment discontinuation due to intolerance	Intolerance
	June 2020	Treatment with regorafenib chemotherapy with disease progression	
	January 2021	Treatment with various targeted therapeutics, including sintilimab and fruquintinib, followed by disease progression.	Disease progression
	April 2021	Informed consent for GCC19CART study obtained	
	April 2021	Leukapheresis	
	May 2021	GCC19CART treatment	
**Patient 05**	February 2019	Diagnosed with locally advanced CRC	
	February 2019	Resection of primary sigmoid colon lesion	
		Adjuvant chemotherapy, including capecitabine, and radiation therapy followed by disease progression	
	November 2019	Treatment with two courses of irinotecan and raltitrexed chemotherapy and cetuximab; discontinued due to intolerance.	Intolerance
		Treatment with eight course of XELOX chemotherapy followed by disease progression.	Disease progression
	October 2020	Treatment of liver metastases with radioablation, followed by disease progression	Disease progression
		Treatment with six courses of irinotecan, pentafluorouracil, and leucovorin chemotherapy, and cetuximab; discontinued because of intolerance.	Intolerance
		Treatment with one course of capecitabine chemotherapy followed by two courses of irinotecan, pentafluoro uracil, and leucovorin chemotherapy, and cetuximab; discontinued because of intolerance.	Intolerance

© 2024 Chen N et al. *JAMA Oncology.*

		Treatment with one course of regorafenib	Disease progression
	May 2021	Informed consent for GCC19CART study obtained	
	June 2021	Leukapheresis	
	June 2021	GCC19CART treatment	
**Patient 06**	June 2019	Diagnosed with locally advanced CRC	
	June 2019	Neoadjuvant therapy with CAPOX chemotherapy	Intolerance
	June 2019	Laparoscopic resection of primary colon lesion.	
	July 2019	Adjuvant therapy with 11 courses of FOLFOX chemotherapy, followed by disease progression.	Disease progression
	July 2020	Treatment with four courses of FOLFIRI chemotherapy and bevacizumab; treatment discontinued because of intolerance	Intolerance
	January 2021	Mesenteric arterial perfusion therapy of carboplatin and etoposide and one course of CAPOX chemotherapy, followed by disease progression.	Disease progression
	March 2021	Treatment with three courses of trifluridine/tipiracil chemotherapy, followed by disease progression.	Disease progression
	June 2021	Informed consent for GCC19CART study obtained	
	June 2021	Leukapheresis	
	July 2021	GCC19CART treatment	
**Patient 07**	October 2017	Diagnosed with mCRC	
	October 2017	Radiofrequency ablation of lung metastases	
	October 2017	Neoadjuvant radiation to rectal primary lesion and capecitabine chemotherapy	
		Resection of primary rectal lesion	
	February 2018	Adjuvant therapy with three courses of capecitabine, then three courses of XELOX chemotherapy, followed by disease progression.	Disease progression
	October 2019	Treatment with once courses of raltitrexed and cisplatin chemotherapy; treatment discontinued because of intolerance.	Intolerance
		Treatment with seven courses of raltitrexed and nedaplatin chemotherapy, then raltitrexed, followed by disease progression.	Stable disease
	September 2020	Treatment with two courses of fruquintinib; treatment discontinued because of intolerance	Intolerance
		Treatment with XELIRI chemotherapy and cetuximab, followed by disease progression.	Disease progression
	July 2021	Informed consent for GCC19CART study obtained	
	July 2021	Leukapheresis	
	August 2021	GCC19CART treatment	
**Patient 08**	January 2018	Diagnosed with locally advanced CRC	
	January 2018	Resection of primary right lesion colon lesions	
	February 2018	Adjuvant chemotherapy with six courses of XELOX chemotherapy	Disease progression
	February 2019	Treatment of mCRC with one course of irinotecan, tegafur, gemcitabine, and oxylatyne potassium chemotherapy	
	March 2019	Treatment with two courses of irinotecan chemotherapy	
	April 2019	Resection of liver metastases	
	May 2019	Treatment with four courses of irinotecan, tegafur, gemcitabine, and oxylatyne, followed by disease progression.	Disease progression
	October 2019	Treatment with eight courses of FOLFIRI chemotherapy and bevacizumab, then seven courses of capecitabine chemotherapy and bevacizumab, followed by disease progression.	Disease progression
	June 2020	Laparoscopic resection of left lower lung cancer and metastatic lesions.	
	July 2020	Treatment with one course of capecitabine chemotherapy and bevacizumab, followed by disease progression.	Disease progression
	August 2020	Treatment with four course of FOLFOX chemotherapy plus trastuzumab, followed by disease progression.	Disease progression
	October 2020	Treatment with one course of trastuzumab and pyrotinib, followed by disease progression.	Disease progression
	December 2020	Treatment with one course of trastuzumab, irinotecan chemotherapy and a mAb targeting PD-1, followed by disease progression.	Disease progression
	February 2021	Microwave ablation of liver metastases	
	February 2021	Treatment of once course of trastuzimab, irinotecan chemotherapy, and a mAb targeting PD-1, followed by three courses of a mAb targeting PD-1.	
	May 2021	Microwave ablation of liver metastases	
	May 2021	Treatment with one course of irinotecan chemotherapy and a mAb targeting PD-1.	
	June 2021	Treatment with one course of a mAb targeting PD-1, followed by disease progression.	Disease progression
	June 2021	Informed consent for GCC19CART study obtained	
	July 2021	Leukapheresis	
	August 2021	GCC19CART treatment	
**Patient 09**	July 2018	Diagnosed with mCRC	
	July 2018	Resection of rectal primary lesion and liver metastasis.	

© 2024 Chen N et al. *JAMA Oncology.*

		Treatment with three courses of CAPOX chemotherapy, followed by disease progression.	Disease progression
		Treatment with two courses of XELIRI chemotherapy and bevacizumab, followed by disease progression.	Disease progression
	December 2018	Radiofrequency ablation of a dominant liver metastasis.	
		Treatment with one course of CAPOX chemotherapy and bevacizumab, followed by disease progression.	Disease progression
		Treatment with two courses of capecitabine chemotherapy, followed by disease progression.	Disease progression
	July 2019	Liver radiofrequency ablation of two liver metastases, followed by disease progression.	Disease progression
		Treatment with twelve courses of apatinib, Tegafur, gemcitabine, and oxylatyne chemotherapy, followed by disease progression	Disease progression
	July 2020	Radiofrequency ablation of lung and liver metastases, followed by disease progression.	Disease progression
		Treatment with three courses of apatinib, tegafur, gemcitabine, and oxylatyne chemotherapy; treatment discontinued due to intolerance.	Intolerance
	March 2021	Informed consent for GCC19CART study obtained	
	March 2021	Leukapheresis	
	April 2021	GCC19CART treatment	
**Patient 10**	January 2019	Diagnosed with locally advanced CRC.	
	January 2019	Resection of primary colon lesion.	
	January 2019	Adjuvant therapy with eight courses of XELOX chemotherapy, followed by disease progression.	Disease progression
	June 2019	Treatment with eight courses of XELIRI chemotherapy and bevacizumab, then capecitabine chemotherapy plus bevacizumab, followed by disease progression.	Disease progression
	March 2021	Informed consent for GCC19CART study obtained	
	March 2021	Leukapheresis	
	April 2021	GCC19CART treatment	
**Patient 11**	May 2018	Diagnosed with locally advanced CRC	
	May 2018	Resection of rectal primary lesion	
	May 2018	Adjuvant therapy with five courses of mFOLFOX chemotherapy, followed by disease progression	Disease progression
	October 2018	Resection of retroperitoneal metastases and radiofrequency ablation of liver metastases.	
	October 2018	Treatment with one course of FOLFIRI chemotherapy, then twelve courses of FOLFIRI chemotherapy and bevacizumab; treatment discontinued because of intolerance.	Intolerance
	June 2019	Treatment with seventeen course of capecitabine and bevacizumab, followed by disease progression.	Disease progression
	June 2020	Radiofrequency ablation of liver metastases.	
	July 2020	Treatment with two courses of FOLFIRINOX chemotherapy and cetuximab; treatment discontinued because of intolerance.	Intolerance
	September 2020	Treatment with three courses of cetuximab, then four course of oxaliplatin and cetuximab, followed by disease progression.	Disease progression
	December 2020	Radiofrequency ablation of liver metastases.	
	February 2021	Treatment with three courses of 5-flurouracil chemotherapy and bevacizumab, followed by disease progression.	Disease progression
	April 2021	Informed consent for GCC19CART study obtained	
	April 2021	Leukapheresis	
	May 2021	GCC19CART treatment	
**Patient 12**	February 2020	Diagnosed with locally advanced CRC	
	March 2020	Laparoscopic resection of primary rectal lesion	
	April 2020	Adjuvant therapy with eight courses of XELOX chemotherapy and one course of capecitabine, followed by disease progression.	Disease progression
	November 2020	Treatment with three courses of FOLFIRI, followed by disease progression.	Disease progression
		Treatment with two courses of trifluridine/tipiracil chemotherapy and bevacizumab, followed by disease progression.	Disease progression
	April 2021	Informed consent for GCC19CART study obtained	
	April 2021	Leukapheresis	
	May 2021	GCC19CART treatment	
**Pt.13**	August 2017	Diagnosed with locally advanced CRC.	
	August 2017	Laparoscopic resection of rectal primary lesion.	
	August 2017	Adjuvant therapy with six course of XELOX chemotherapy, followed by disease progression	Disease progression
	February 2018	Treatment with six courses of irinotecan and raltitrexed chemotherapy, and cetuximab; treatment discontinued because of disease progression.	Disease progression
	March 2018	Treatment with ten course of irinotecan and cetuximab, followed by disease progression.	Disease progression
	September 2018	Resection of liver metastases.	
	January 2019 – October 2019	Radiofrequency ablation of liver metastases.	

© 2024 Chen N et al. *JAMA Oncology.*

	November 2019	Microwave ablation of liver metastases, followed by disease progression.	Disease progression
	December 2019	Treatment with five courses of XELOX chemotherapy and bevacizumab, followed by disease progression.	Disease progression
		Treatment with one courses of irinotecan and raltitrexed chemotherapy; treatment discontinued because of intolerance.	Intolerance
	May 2020	Resection of liver metastases	
	May 2020	Treatment with fifteen course of capecitabine chemotherapy and bevacizumab, followed by disease progression.	Disease progression
	May 2021	Informed consent for GCC19CART study obtained	
	May 2021	Leukapheresis	
	June 2021	GCC19CART treatment	
**Patient 14**	October 2019	Diagnosed with locally advanced CRC	
	October 2019	Resection of primary rectal lesion, followed by disease progression.	Disease progression
	November 2019	Treatment with once course of CAPOX chemotherapy.	
	December 2019	Radiofrequency ablation of liver metastases	
		Treatment with six courses of CAPOX chemotherapy, followed by disease progression.	Disease progression
	June 2020	Treatment with four courses of FOLFIRI chemotherapy, with a partial response.	Partial response
	August 2020	Resection of liver metastases and radiofrequency ablation of liver metastases, followed by disease progression.	Disease progression
	October 2020	Treatment with eight courses of irinotecan and cetuximab, followed by disease progression.	Disease progression
	April 2021	Treatment with once course of bevacizumab, then two courses of capecitabine chemotherapy and bevacizumab, followed by disease progression	Disease progression
	July 2021	Informed consent for GCC19CART study obtained	
	July 2021	Leukapheresis	
	September 2021	GCC19CART treatment	
**Patient 15**	March 2019	Diagnosed with locally advanced CRC	
	March 2019	Resection of primary colon lesion.	
	March 2019	Adjuvant therapy with eight courses of oxaliplatin, tegafur, gemcitabine, and oxylatyne chemotherapy, followed by disease progression.	Disease progression
	July 2019	Treatment with two courses of irinotecan and capecitabine chemotherapy; treatment discontinued because of intolerance.	Intolerance
	January 2021	Treatment with traditional Chinese medicine, followed by disease progression.	Disease progression
	August 2021	Informed consent for GCC19CART study obtained	
	August 2021	Leukapheresis	
	September 2021	GCC19CART treatment	

Abbreviations: CAPOX, capecitabine and oxaliplatin; CRC, colorectal cancer; FOLFIRI , folinic acid, 5-fluorouracil, and irinotecan; FOLFOX, folinic acid, 5-fluorouracil, and oxaliplatin; XELIRI capecitabine and irinotecan; FOLFIRINOX, Folinic acid, 5-fluorouracil, oxaliplatin; mCRC, metastatic colorectal cancer; mFOLFOX - modified FOLFOX; mAb, monoclonal Antibody; PD-1, programmed cell death protein-1; XELOX, capecitabine and oxaliplatin.

© 2024 Chen N et al. *JAMA Oncology.*

eTable 3. CAR-T cell expansion post infusion for each patient

Studies	Infusion dose	Peak GCC CAR-T or CART19 cell number*10^3/mL in peripheral blood[a]	Mean peak GCC CAR-T or CART19 cell number*10^3/mL in peripheral blood	
Frey et al,.2020[8]	CART19 (1.08-50 x 10^7 cells)	110 (40-210)	110	
Neelapu et al,.2017[9]	CART19 (2 x 10^6 cells per kg)	90	NA[b]	
Current study	GCC19CART (1 x 10^6 cells per kg)	Patient 1 - 365	354 (51-1050)	537 (51-1302)
		Patient 2 - 51		
		Patient 3 - 358		
		Patient 4 - 1050		
		Patient 5 - 428		
		Patient 6 - 118		
		Patient 7 - 325		
		Patient 8 - 135		
	(2 x 10^6 cells per kg)	Patient 9 - 592	746 (376-1302)	
		Patient 10 - 602		
		Patient 11 - 1302		
		Patient 12 - 376		
		Patient 13 - 662		
		Patient 14 - 1233		
		Patient 15 - 452		

[a] The number of anti-GCC CAR-T cell levels were quantified by flow cytometry at peak expansion day in all 15 patients who treated with GCC19CART, as described in the article.[8,9] PB, peripheral blood.

[b] The mean cannot be estimated due to the incomplete data.

© 2024 Chen N et al. *JAMA Oncology.*

eFigure 1. Schematic diagram of the GCC19CAR constructs used in this study. Four different lentiviral vectors containing one anti-GCC CAR and three anti-CD19 CAR, simultaneously, were transduced to T cells from the patient. The figure is not drawn to scale.

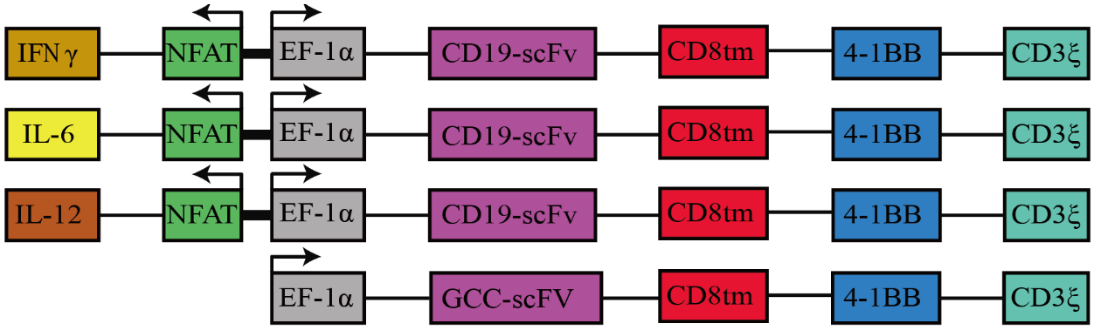

© 2024 Chen N et al. *JAMA Oncology.*

eFigure 2. GCC CAR-T and CD19 CAR-T cell proliferation. (A) The percentages of proliferating CD4+ GCC CAR-T cells (left) and proliferating CD4+ CD19 CAR-T cells (right) in the presence or absence of B cells. **(B)** The absolute numbers of proliferating CD4+ GCC CAR-T cells (left) and proliferating CD4+ CD19 CAR-T cells (right) in the presence or absence of B cells. **(C)** The percentages of proliferating CD8+ GCC CAR-T cells (left) and proliferating CD8+ CD19 CAR-T cells (right) in the presence or absence of B cells. **(D)** The absolute numbers of proliferating CD8+ GCC CAR T cells (left)) and proliferating CD8+ CD19 CAR-T cells (right) in the presence or absence of B cells. Effector to target (E:T) cell ratio was 1:1. Ns, not significant; *, P< 0.05; **, P< 0.01; ***, P< 0.001; and ****P< 0.0001.

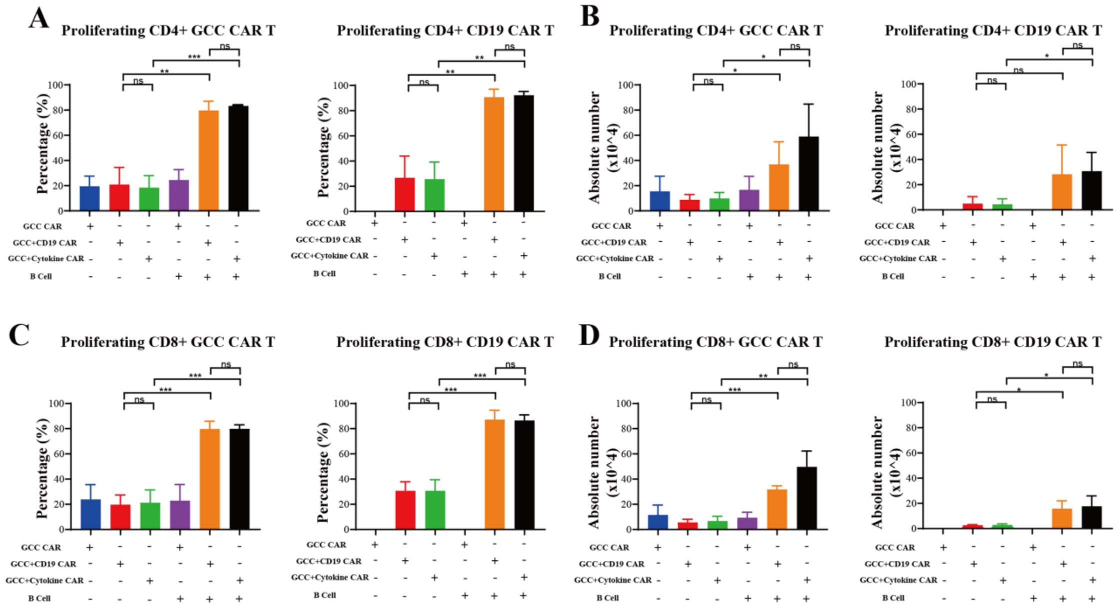

© 2024 Chen N et al. *JAMA Oncology.*

eFigure 3. CAR-T cell phenotypes. (A) The percentages of CD137+ T cells in GCC CAR-T (left) and CD19 CAR-T cells (right) in the six sub-groups. **(B)** The percentages of CD25+ T cells in GCC CAR-T (left) and CD19 CAR-T cells (right). **(C)** The percentages of PD-1+ T cells in GCC CAR-T (left) and CD19 CAR-T cells (right). **(D)** The percentages of CD45RA+ CD62L- T cells in GCC CAR T (left) and CD19 CAR-T cells (right). **(E)** The percentage of CD45RA- CD62L- T cells in GCC CAR-T (left) and CD19 CAR-T cells (right). **(F)** The percentage of CD45RA- CD62L+ T cells in GCC CAR-T (left) and CD19 CAR-T cells (right). Effector to target (E:T) cell ratio was 1:1. Ns, not significant; *, P< 0.05; **, P< 0.01; ***, P< 0.001; and ****P< 0.0001.

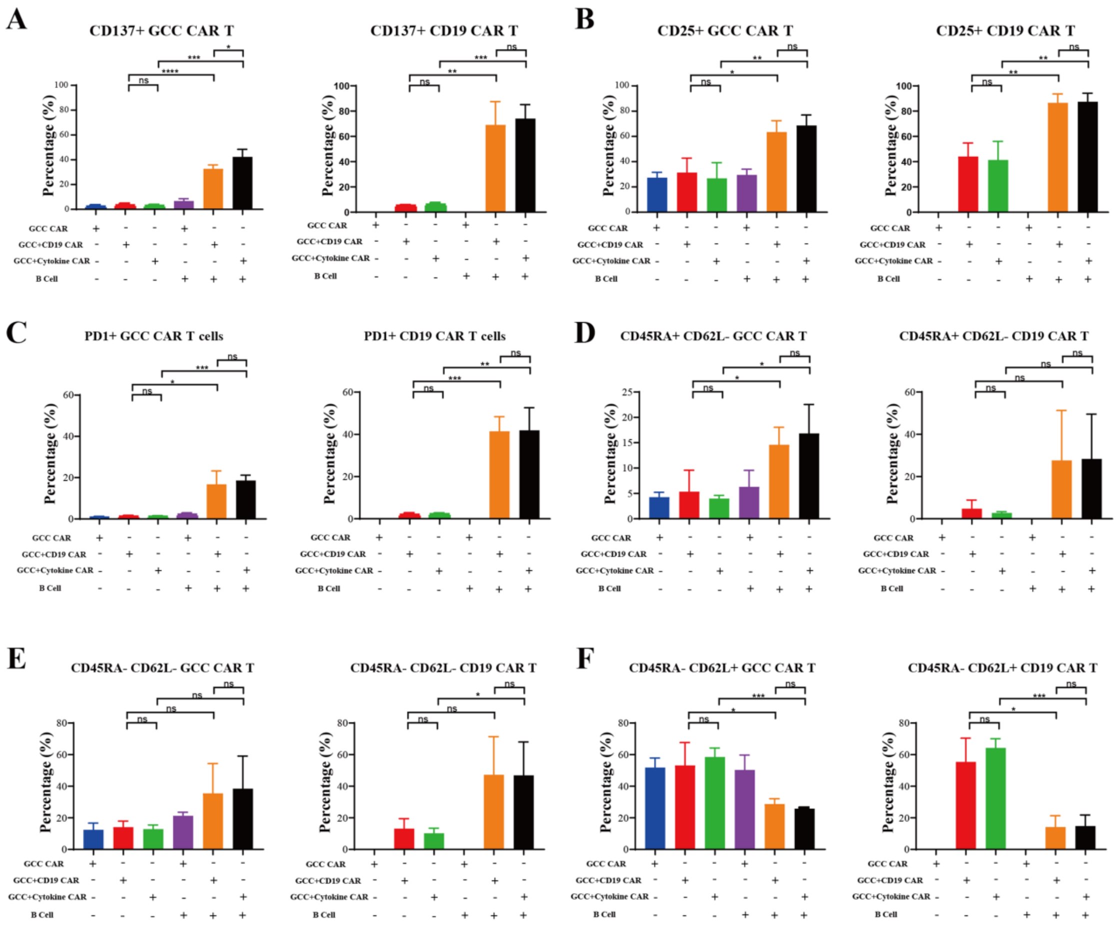

© 2024 Chen N et al. *JAMA Oncology.*

eFigure 4. CAR-T cell cytokines. (A) The mean values of IL-2, IL-6, IFN-γ, GZMB, IL-12, and tumor necrosis factor-α (TNF-α) from 6 sub-groups. **(B)** The proportions of intracellular IFN-γ+ GCC CAR T (left) and intracellular IFN-γ+ CD19 CAR T cells (right). **(C)** The proportions of intracellular GZMB+ GCC CAR T (left) and intracellular GZMB+ CD19 CAR T cells (right). Effector to target (E:T) cell ratio was 1:1. Ns, not significant; *, P< 0.05; **, P< 0.01; ***, P< 0.001; and ****, P< 0.0001.

A

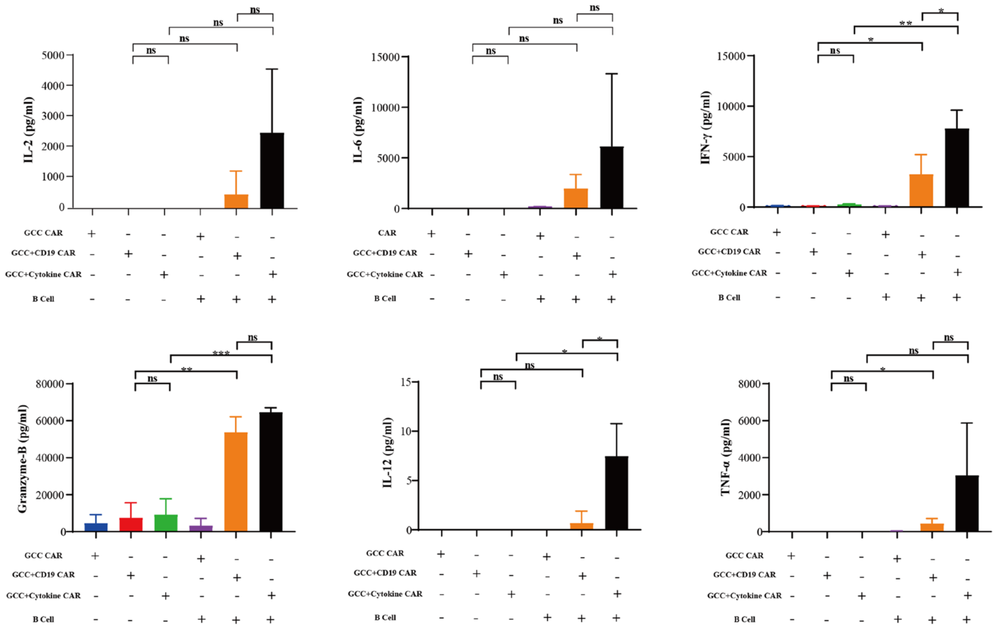

B

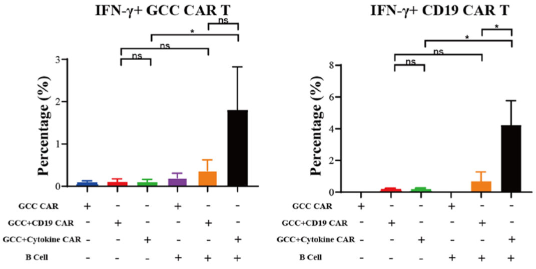

C

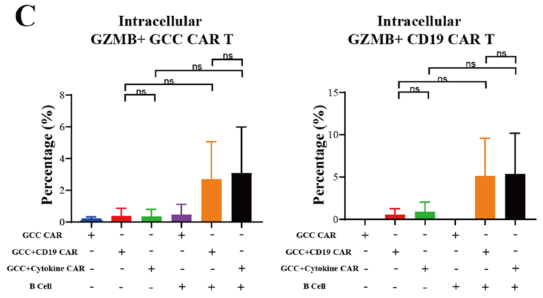

© 2024 Chen N et al. *JAMA Oncology.*

eFigure 5. CD19 CAR-T cells enhance the cytotoxicity of GCC CAR-T cells *in vivo.* **(A)** The scheme of blood sampling relative to the administration of immune and CAR cells in T84/B cell xenograft NOG mice. **(B)** The levels of IFN-γ (left) and GZMB (right panel) in blood sampled on day 14 and measured using a cytometric bead array assay. **(C)** The expansion of CD19 CAR-T cells in blood sampled on days 7, 11, 14, and 17 and assessed using flow cytometry. **(D)** The expansion of GCC CAR-T cells in blood sampled from each of the three groups on days 7, 11, 14, and 17, and analyzed using flow cytometry. **(E)** The tumor volumes of the mice at different time points. Ns, not significant; *, P< 0.05; **, P< 0.01; ***, P< 0.001; and ****P< 0.0001.

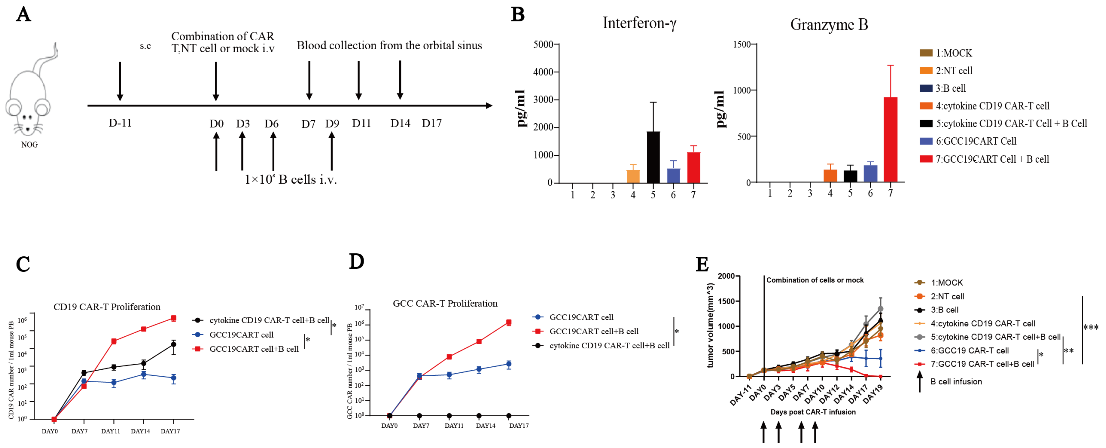

© 2024 Chen N et al. *JAMA Oncology.*

eFigure 6. GCC staining by immunohistochemistry of primary and metastatic colorectal carcinoma samples of the study patients. (A) Immunehistographs of GCC protein expression in tissues sampled from either primary or metastatic colorectal carcinoma lesions from each patient who enrolled in the study. **(B)** Immunolocalization and grades of GCC protein staining intensity, with grades ranging from negative to +3) in colorectal cancer tissue samples (magnification, x200).

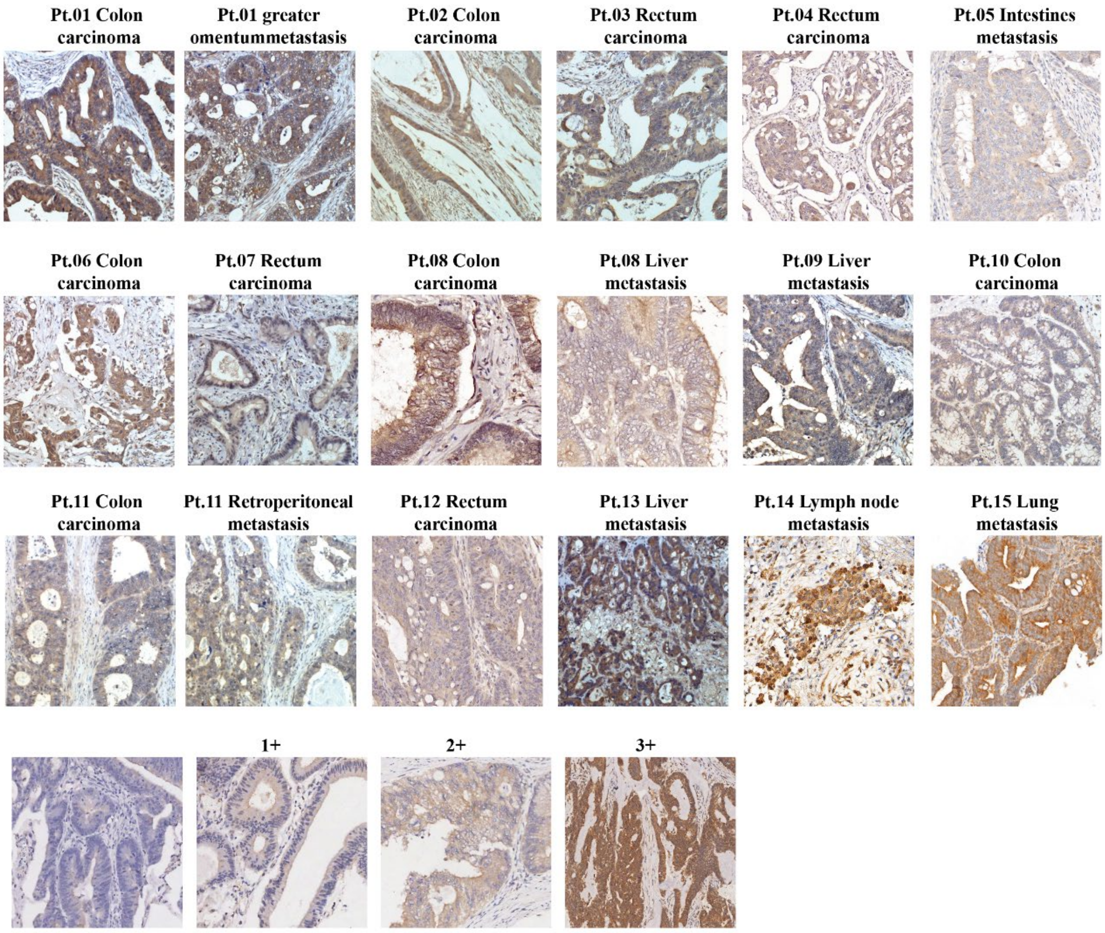

© 2024 Chen N et al. *JAMA Oncology.*

eFigure 7. Characterization of each infusion product by quantitative polymerase chain reaction (qPCR). **(A)** The frequencies of transduced T cells expressing different vectors for each product used to treat patients in the study. **(B)** The vector copy number per CAR-T cell in the infusion product.

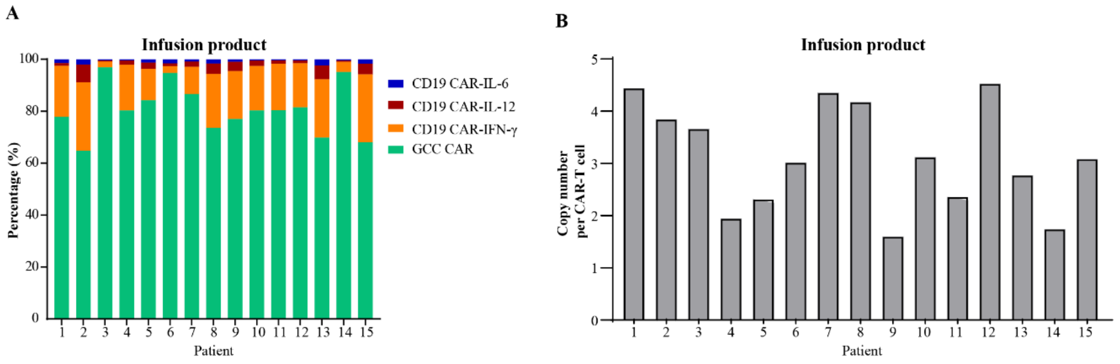

© 2024 Chen N et al. *JAMA Oncology.*

eFigure 8. Characterization of GCC19CART administered to each patient. (A) The percentages of CD3+ T-cells in infusion product administered to each study patient. **(B), (C)** The percentages of CD8+ and CD4+ cells in CD3+ T cells in each infusion product. **(D)** The percentage of CAR+ (chimeric antigen receptor positive) cells within the CD3+ T cell population for each infusion product.

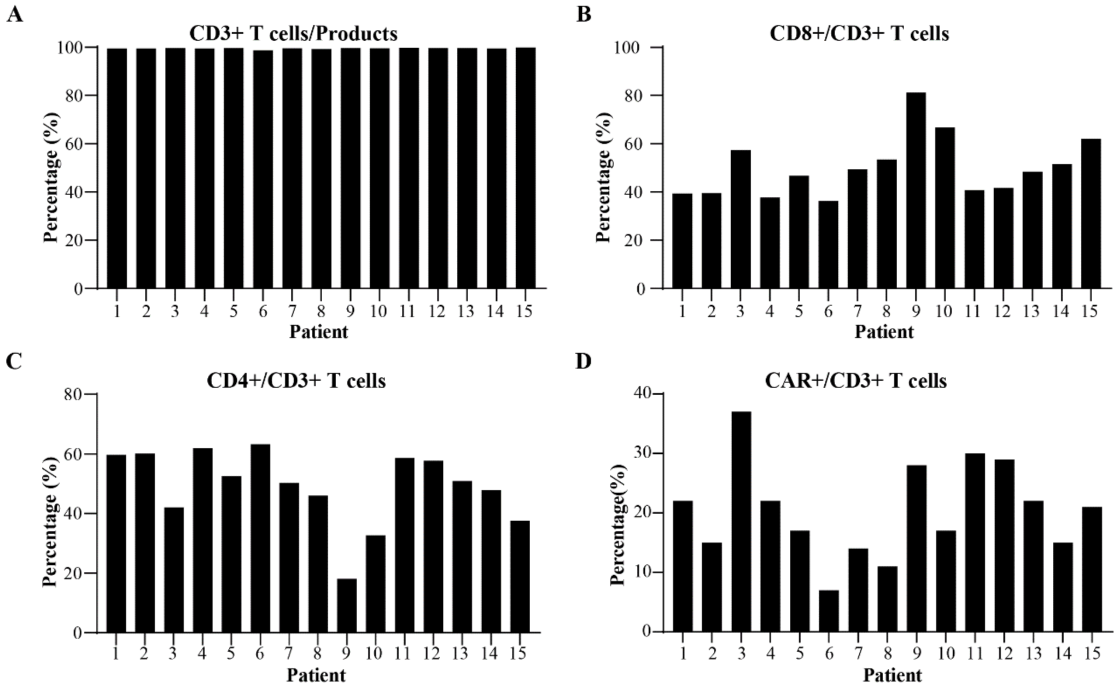

© 2024 Chen N et al. *JAMA Oncology.*

eFigure 9. CAR T-cell proliferation and cytokine release after GCC19CART treatment. (A-D) Measurements of CAR vector copy number in peripheral blood samples as assessed by quantitative polymerase chain reaction assay. Each point on the solid line indicates the median copy numbers at the various time points at each of the two dose levels. **(E-F)** Measurements of CAR-T cells in peripheral blood samples as assessed by flow cytometry. Each point on the solid line indicates the median cell numbers at the various time points for each dose level. **(G-O)** Serial measurements of the cytokines including interleukin-2 (IL2), interleukin-6 (IL6), interleukin-8 (IL10), interleukin-12 (IL12), interferon-γ, granzyme B, monocyte chemoattractant protein-1 (MCP-1) and tumor necrosis factor-α (TNF-α). The standard curve range for all the analytes was 0 to 5000 pg per milliliter. (ns, not significant; *; P < 0·05, **; P < 0·01; ***, P < 0·001, ****, P < 0·0001.

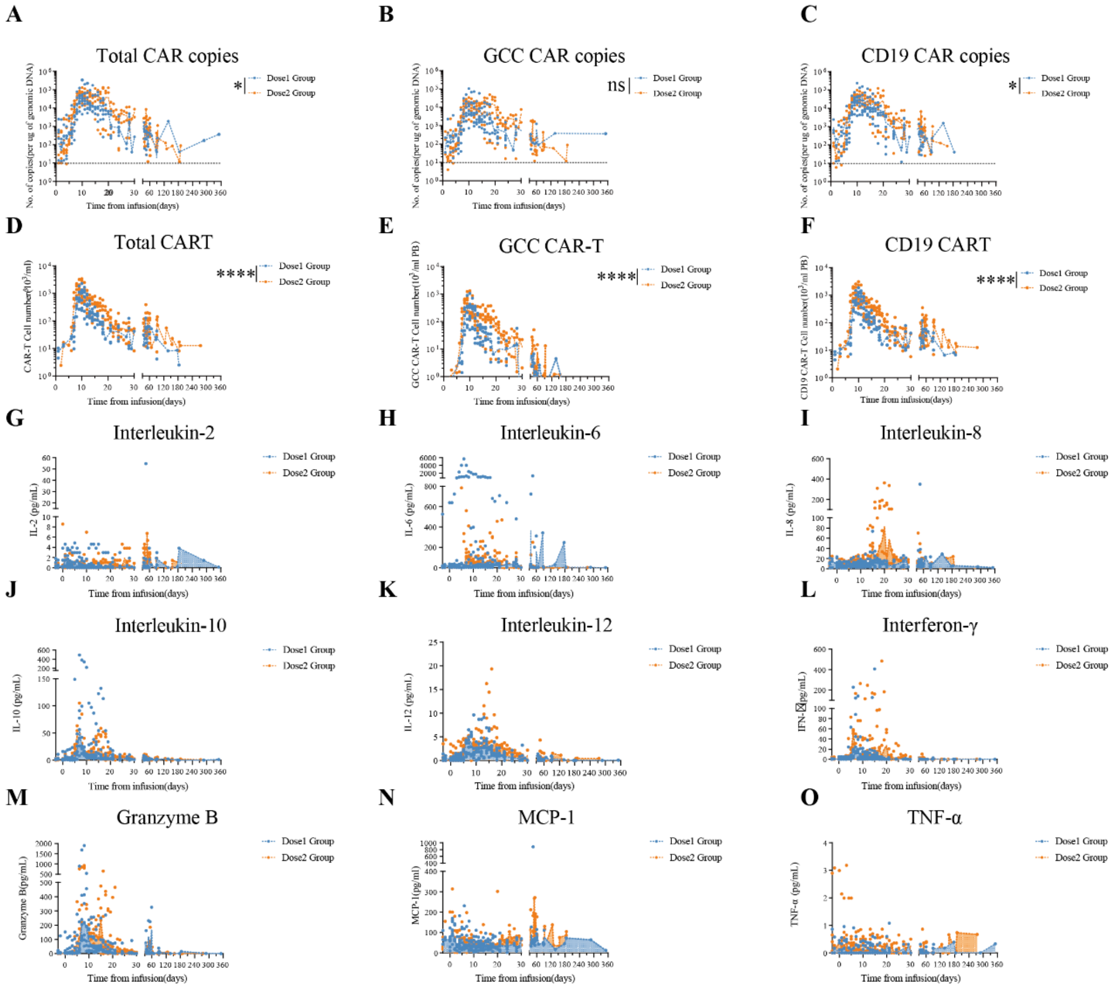

© 2024 Chen N et al. *JAMA Oncology.*

eFigure 10. CAR copy number in transduced T cells that express different transgenes in peripheral blood samples, as assessed by quantitative polymerase chain reaction (qPCR).

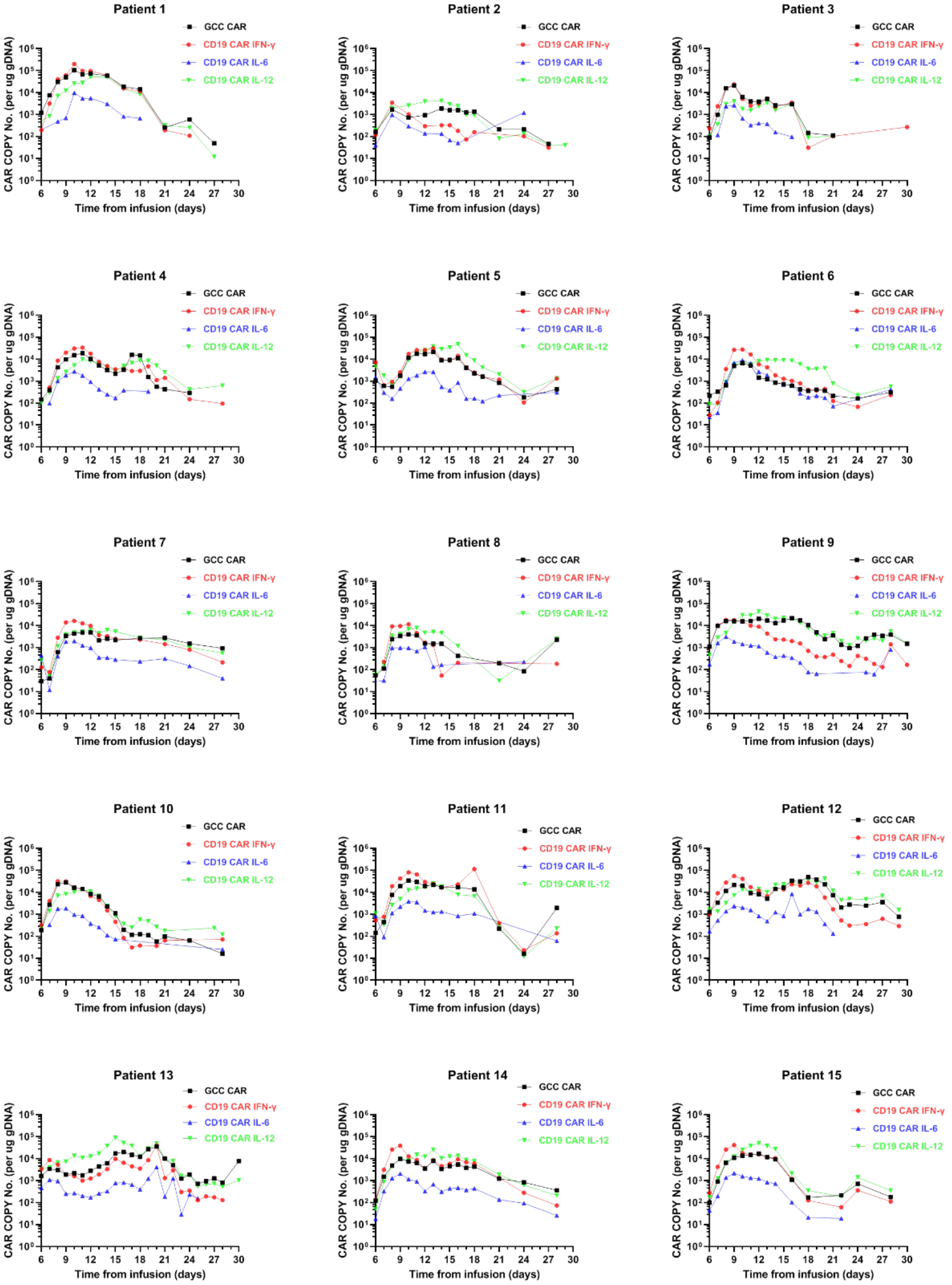

© 2024 Chen N et al. *JAMA Oncology.*

eFigure 11. GCC CAR-T cell numbers, as assessed by both flow cytometry and qPCR in each of the fifteen study patients.

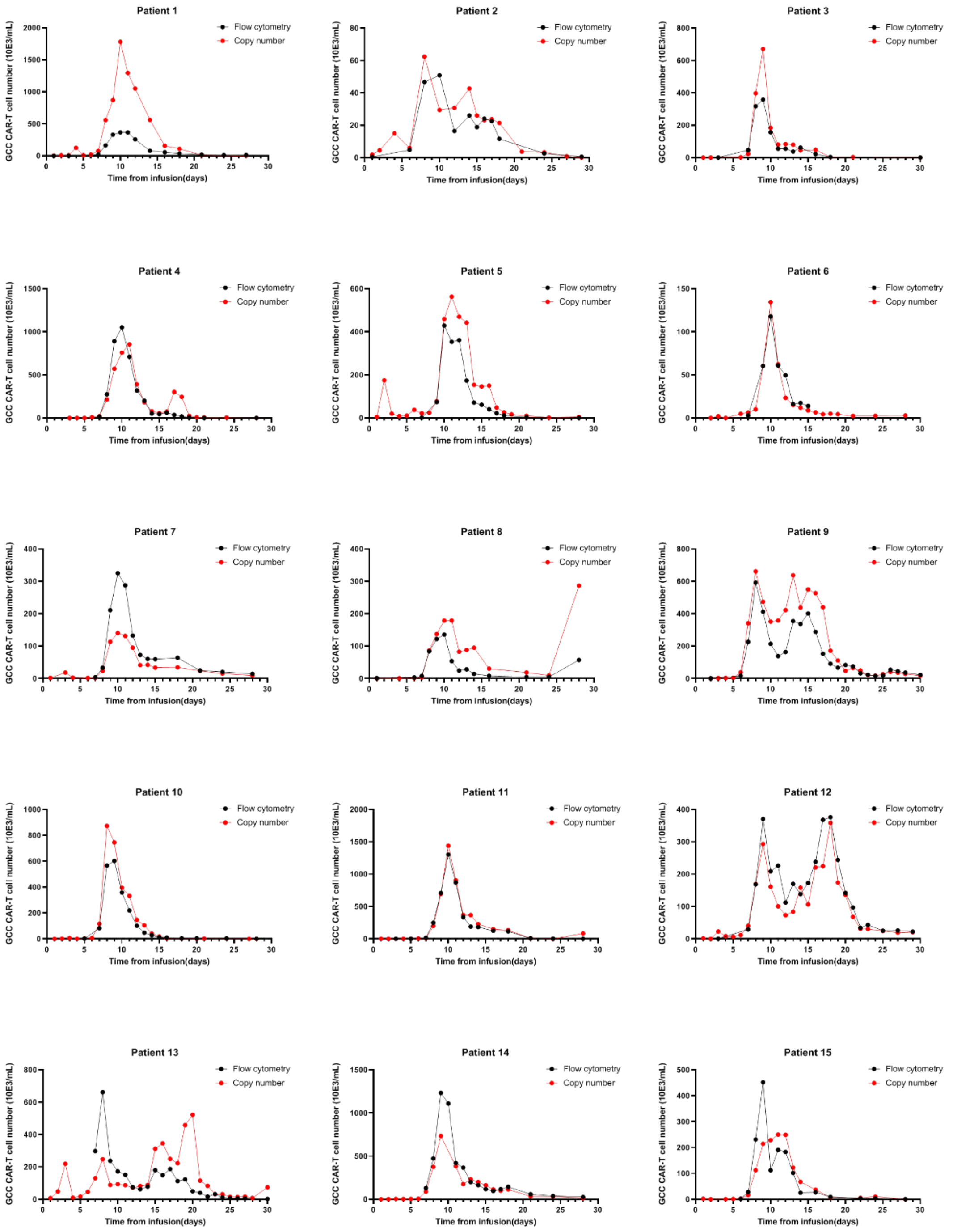

© 2024 Chen N et al. *JAMA Oncology.*

eFigure 12. CD19 CAR-T cell numbers as assessed by both flow cytometry and qPCR in the fifteen study patients.

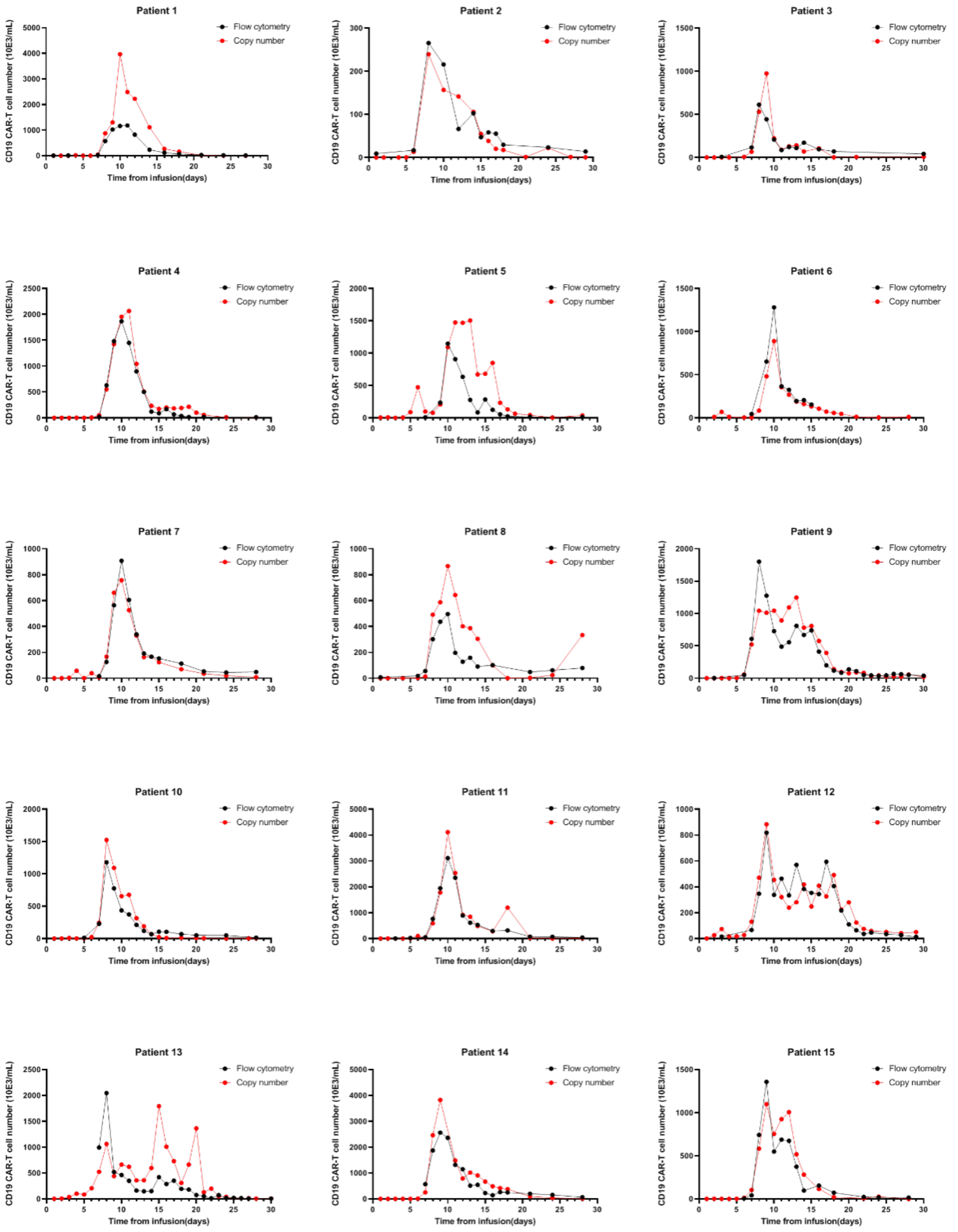

© 2024 Chen N et al. *JAMA Oncology.*

eFigure 13. Clinical Response in 15 Heavy-Pretreated Patients. (A) Spider plots demonstrating percent changes in tumor measurements from pretreatment to each time of CT imaging and RECIST 1.1. assessments. **(B)** Spider plots demonstrating percent changes in tumor measurements from pretreatment to each time of PET imaging and PERCIST 1.0 assessments. Red triangles indicate the first occurrence of progressive disease. The area between the dashed lines indicates stable disease.

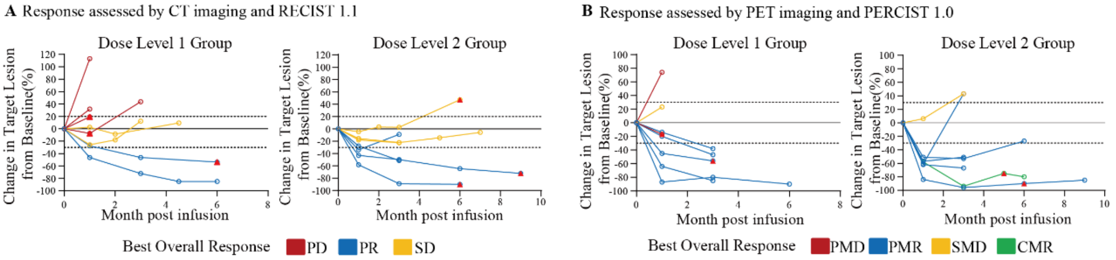

© 2024 Chen N et al. *JAMA Oncology.*

eFigure 14. Single-cell RNA-sequencing from colorectal cancer biopsies pre- and post-treatment with GCC19CART. (A) The proportions of CAR T-cell subsets relative to the total T-cell population. **(B)** The proportion of the GCC19CAR T-cell subset as relative to the total CAR T-cell population. **(C)** The proportion of cancer cell subsets relative to all cells. **(D)** The ratio of regulatory T cells to CD8+ T-cells in tumors as a function of time post-treatment. **(E)** Bar plot depicting the distribution of TGF-β- and TGF-β+ B-cells in different samples. **(F)** The frequency of M1/M2 macrophage subsets as a proportion of all macrophages. **(G)** The T-cell clones in the two tumor biopsies, which were divided into three groups. **(H-I)** The frequency of TCR clones of the three groups as a proportion of all TCR clones.

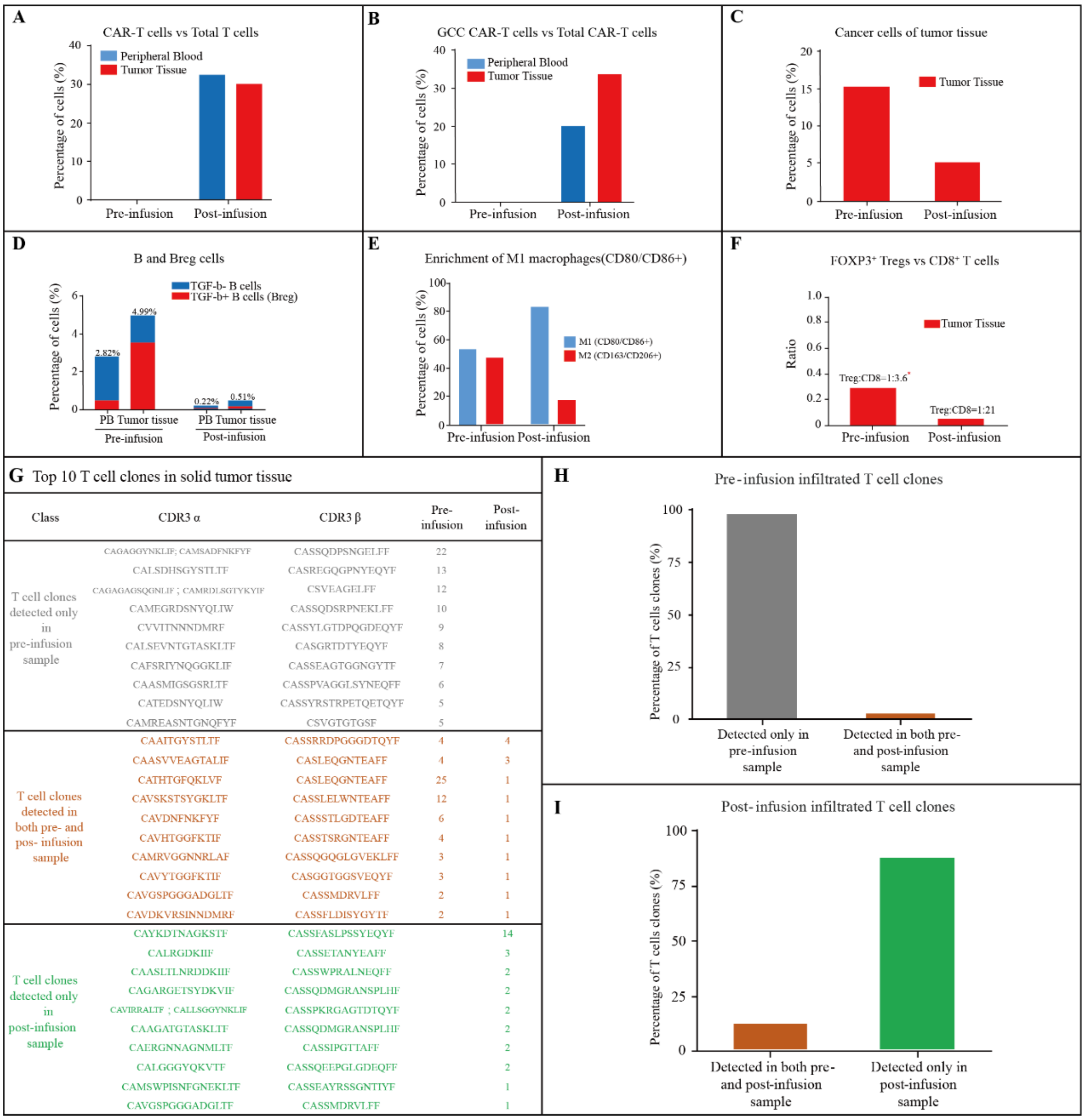

G Top 10 T cell clones in solid tumor tissue

Class	CDR3 α	CDR3 β	Pre-infusion	Post-infusion
T cell clones detected only in pre-infusion sample	CAGAGGYNKLIF; CAMSADFNKFYF	CASSQDPSNGELFF	22	
	CALSDHSGYSTLTF	CASREGQGPNYEQYF	13	
	CAGAGAGSQGNLIF ; CAMRDLSGTYKYIF	CSVEAGELFF	12	
	CAMEGRDSNYQLIW	CASSQDSRPNEKLFF	10	
	CVVITNNNDMRF	CASSYLGTDPQGDEQYF	9	
	CALSEVNTGTASKLTF	CASGRTDTYEQYF	8	
	CAFSRIYNQGGKLIF	CASSEAGTGGNGYTF	7	
	CAASMIGSGSRLTF	CASSPVAGGLSYNEQFF	6	
	CATEDSNYQLIW	CASSYRSTRPETQETQYF	5	
	CAMREASNTGNQFYF	CSVGTGTGSF	5	
T cell clones detected in both pre- and pos- infusion sample	CAAITGYSTLTF	CASSRRDPGGGDTQYF	4	4
	CAASVVEAGTALIF	CASLEQGNTEAFF	4	3
	CATHTGFQKLVF	CASLEQGNTEAFF	25	1
	CAVSKSTSYGKLTF	CASSLELWNTEAFF	12	1
	CAVDNFNKFYF	CASSSTLGDTEAFF	6	1
	CAVHTGGFKTIF	CASSTSRGNTEAFF	4	1
	CAMRVGGNNRLAF	CASSQGQGLGVEKLFF	3	1
	CAVYTGGFKTIF	CASGGTGGSVEQYF	3	1
	CAVGSPGGGADGLTF	CASSMDRVLFF	2	1
	CAVDKVRSINNDMRF	CASSFLDISYGYTF	2	1
T cell clones detected only in post-infusion sample	CAYKDTNAGKSTF	CASSFASLPSSYEQYF		14
	CALRGDKIIF	CASSETANYEAFF		3
	CAASLTLNRDDKIIF	CASSWPRALNEQFF		2
	CAGARGETSYDKVIF	CASSQDMGRANSPLHF		2
	CAVIRRALTF ; CALLSGGYNKLIF	CASSPKRGAGTDTQYF		2
	CAAGATGTASKLTF	CASSQDMGRANSPLHF		2
	CAERGNNAGNMLTF	CASSIPGTTAFF		2
	CALGGGYQKVTF	CASSQEEPGLGDEQFF		2
	CAMSWPISNFGNEKLTF	CASSEAYRSSGNTIYF		1
	CAVGSPGGGADGLTF	CASSMDRVLFF		1

eFigure 15. Single cell clustering and downstream analysis. (A) 19 different clusters from tumor biopsies of Patient 15 sampled before and after GCC19CART treatment using unsupervised analysis. **(B)** The percentages of cells in each cluster as a function of the total number of cells before and after GCC19CART treatment; **(C)** Volcano plot showing differentially expressed genes in solid tumor tissue macrophage (10X genomics scRNA-seq). P-value < 0.05, Two-sided unpaired limma moderated t-test; log2 (fold change) ≥ 1.5. **(D)** GSEA analyses for upregulated genes (top) and downregulated genes (bottom) with significant differences in tumor associated macrophages (TAMs) post-infusion and pre-infusion ($P < 0.05$). **(E)** Fold changes in upregulated genes (top) and downregulated genes (bottom) in non-CAR-T cells (NTs) post-infusion and pre-infusion ($P < 0.05$). **(F)** Fold changes in upregulated genes (top) and downregulated genes (bottom) in NK cells post-infusion and pre-infusion ($P < 0.05$).

© 2024 Chen N et al. *JAMA Oncology*.

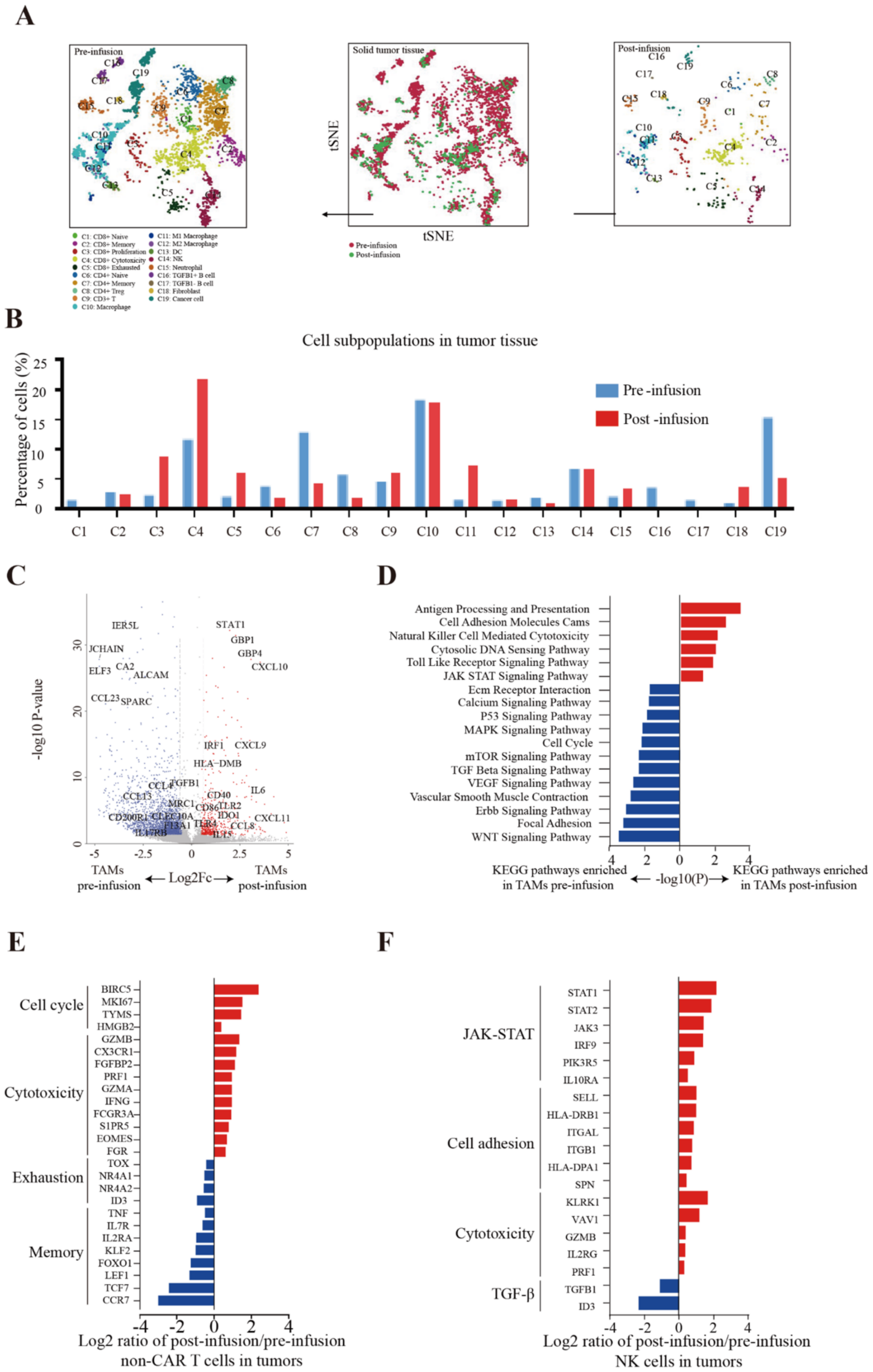

© 2024 Chen N et al. *JAMA Oncology.*

eFigure 16. Imaging from patient 1 after CAR-T cells infusion. These figures show sequential CT (top panel) and FDG-PET (bottom panel) images from patient 1 treated with GCC19CART 1 x 10^6 cells per kg of body weight. Imaging was performed pretreatment and 1-, 3-, and 6-months post-treatment. CT imaging demonstrates a partial response of target lesions in the abdomen and pelvis (red perpendicular lines), as well as regression of nontarget lesions (not shown), which was confirmed on sequential imaging to date. FDP-PET emission from liver and pelvic metastases (baseline, red circles) demonstrates a partial metabolic response on all imaging performed after treatment, with faint FDG uptake observed. The patient had a confirmed partial response (RECIST 1.1) and a confirmed partial metabolic response (PERCIST 1.0).

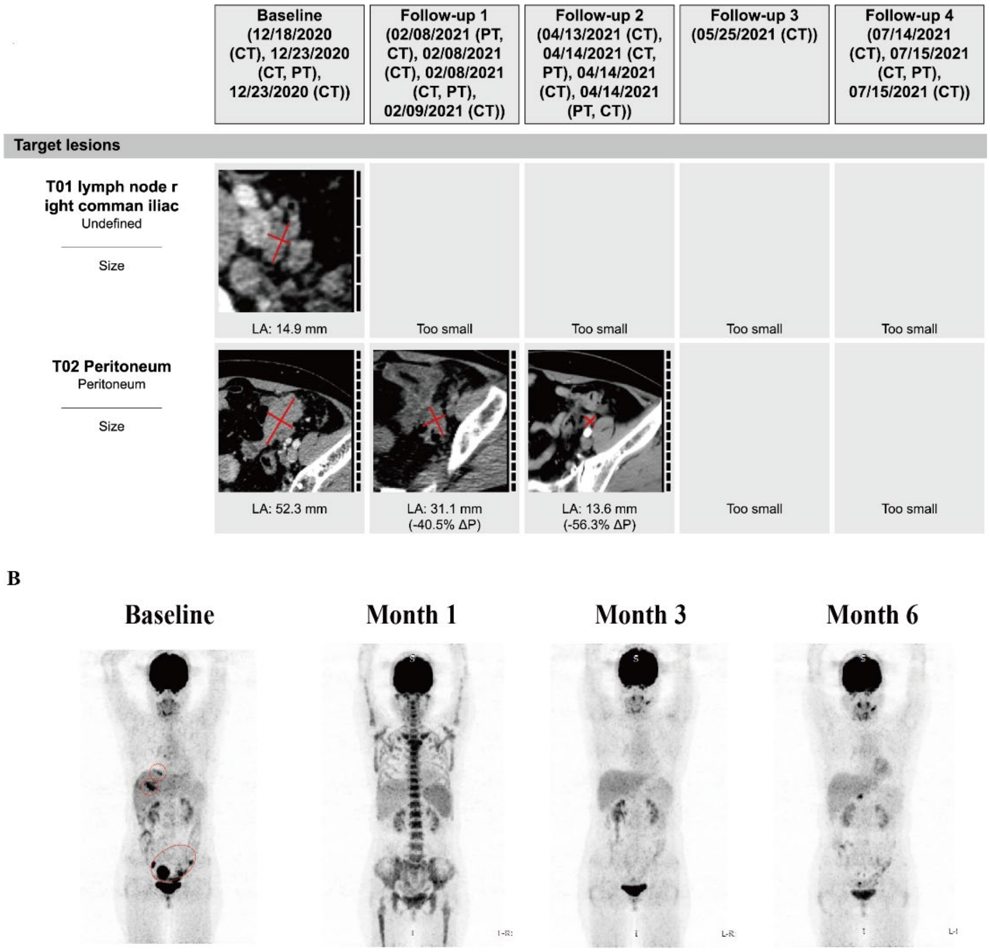

eFigure 17. Imaging results from patient 4. These figures shows sequential computerized tomographic (CT; left panel) and fluorodeoxyglucose positron emission tomographic (FDG-PET; right panel) images from patient 4 treated with GCC19CART 1 x 10^6 cells per kg of body weight. Imaging was performed pretreatment and 1- and 3-months post-treatment. The patient demonstrated a partial response, as evaluated by CT imaging of metastases to liver and pancreas (arrows) using RECIST 1.1 criteria. PET emission from metastases above and below diaphragm, as well as from liver (light arrows) and pancreas (dark arrows), progressively decrease on successive imaging following treatment. There is no FDG uptake in metastases above and below the diaphragm, including the liver and pancreas, 3 months post-treatment. The patient had a partial metabolic responses when assessed on PET imaging using PERCIST 1.0 criteria, which was an exploratory endpoint.

© 2024 Chen N et al. *JAMA Oncology.*

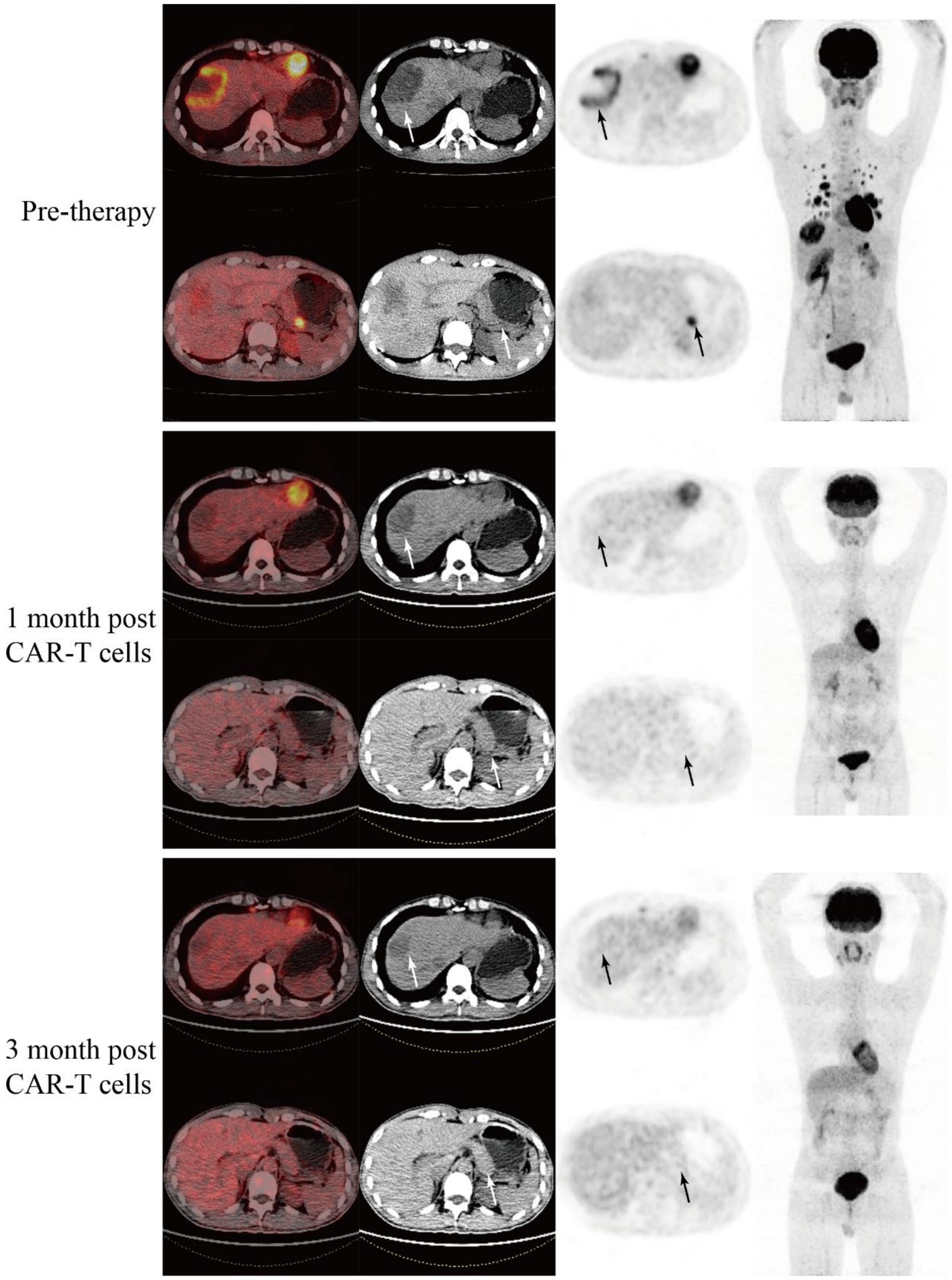

© 2024 Chen N et al. *JAMA Oncology.*

eFigure 18. Imaging results from patient 9. These figures shows sequential CT (left panel) and FDG-PET (right panel) images from patient 9 treated with GCC19CART 2 x 10^6 cells per kg of body weight. Imaging was performed pretreatment and 1- and 3-months post-treatment. Both the size of two target liver metastases (white arrows) and the uptake of FDG-PET of lesions above and below the diaphragm (black arrows) decrease successively from pretreatment to 3 months post-treatment. At 3 months post-treatment, there has been notable reduction in the size of liver metastases and no FDG uptake in the liver. The patient demonstrated a partial response as the best response (RECIST 1.1 criteria). Additionally, a partial metabolic response was observed as the best response using FDG-PET (PERCIST 1.0 criteria), which was an exploratory endpoint.

© 2024 Chen N et al. *JAMA Oncology.*

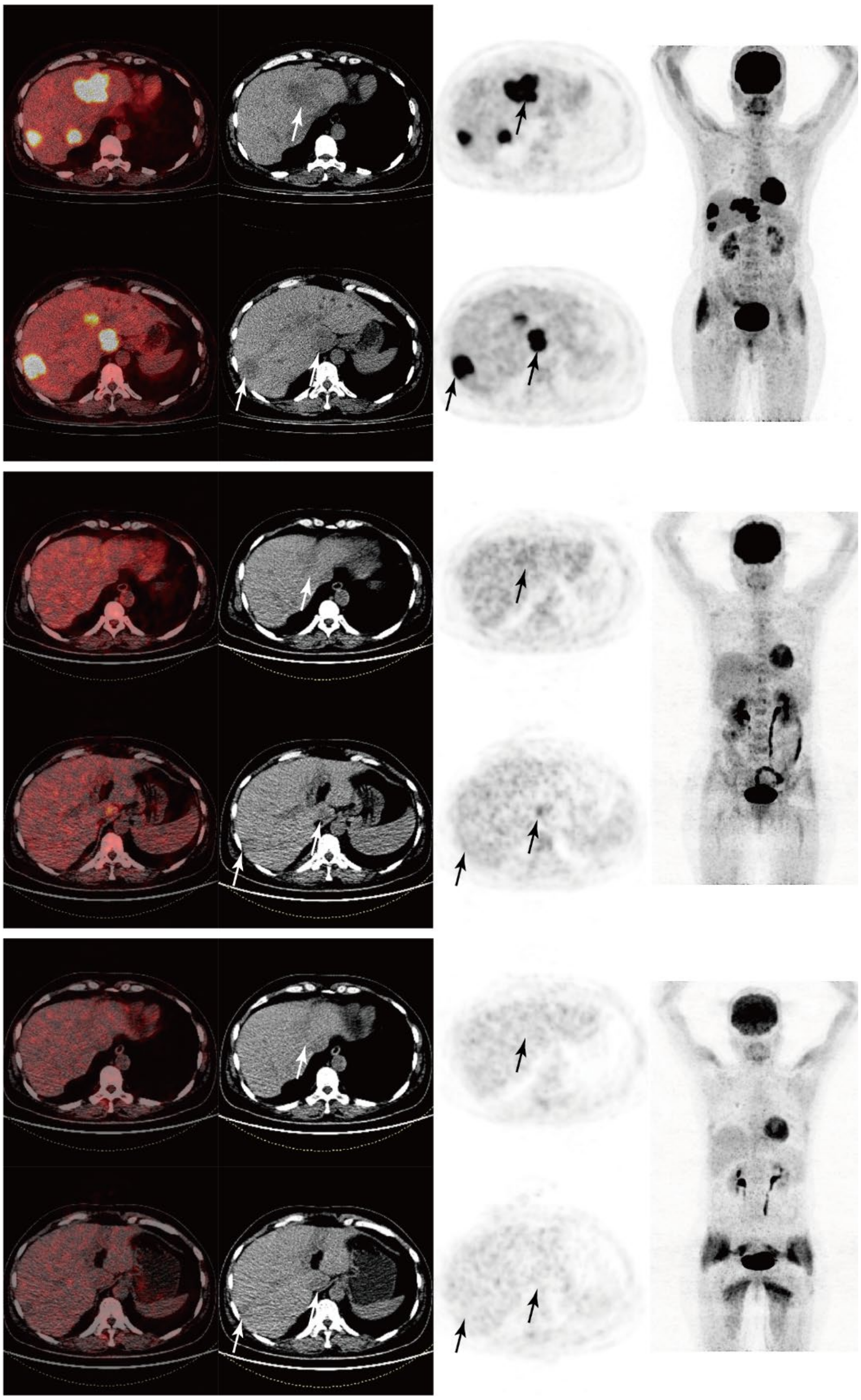

© 2024 Chen N et al. *JAMA Oncology.*

eFigure 19. Response rate in patients after CAR-T cells infusion. (A) The relative distribution of clinical response by phenotype. P-value determined by Fisher's exact test. **(B)** The maximum percentage changes for patients without and with liver metastasis. PD, progressive disease; SD, stable disease; PR, partial response.

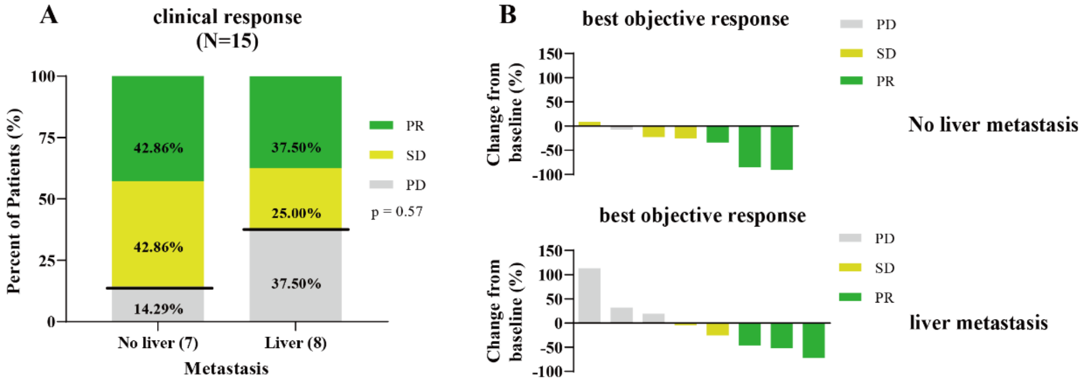

© 2024 Chen N et al. *JAMA Oncology.*

eReferences

1. Zhang L, Li Z, Skrzypczynska KM, et al. Single-Cell Analyses Inform Mechanisms of Myeloid-Targeted Therapies in Colon Cancer. *Cell.* 2020;181(2):442-459.e429. doi:10.1016/j.cell.2020.03.048.

2. Che LH, Liu JW, Huo JP, et al. A single-cell atlas of liver metastases of colorectal cancer reveals reprogramming of the tumor microenvironment in response to preoperative chemotherapy. *Cell Discov.* 2021;7(1):80. doi:10.1038/s41421-021-00312-y.

3. Dull T, Zufferey R, Kelly M, et al. A third-generation lentivirus vector with a conditional packaging system. *J Virol.* 1998;72(11):8463-8471. doi:10.1128/JVI.72.11.8463-8471.1998.

4. Perry C, Rayat A. Lentiviral Vector Bioprocessing. *Viruses.* 2021;13(2):268. doi:10.3390/v13020268.

5. Magee MS, Abraham TS, Baybutt TR, et al. Human GUCY2C-Targeted Chimeric Antigen Receptor (CAR)-Expressing T Cells Eliminate Colorectal Cancer Metastases. *Cancer Immunol Res.* 2018;6(5):509-516. doi:10.1158/2326-6066.CIR-16-0362.

6. R Core Team R. R: A language and environment for statistical computing. *The R Foundation.* 2013. https://www.r-project.org/.

7. Hänzelmann S, Castelo R, Guinney J. GSVA: gene set variation analysis for microarray and RNA-seq data. *BMC Bioinformatics.* 2013;14:7. doi:10.1186/1471-2105-14-7.

8. Frey NV, Gill S, Hexner EO, et al. Long-Term Outcomes From a Randomized Dose Optimization Study of Chimeric Antigen Receptor Modified T Cells in Relapsed Chronic Lymphocytic Leukemia. *J Clin Oncol.* 2020;38(25):2862-2871. doi:10.1200/jco.19.03237.

9. Neelapu SS, Locke FL, Bartlett NL, et al. Axicabtagene Ciloleucel CAR T-Cell Therapy in Refractory Large B-Cell Lymphoma. *N Engl J Med.* 2017;377(26):2531-2544. doi:10.1056/NEJMoa1707447.

© 2024 Chen N et al. *JAMA Oncology.*
