# Supplementary material for: Chimeric Antigen Receptor T Cells Targeting CD19 and GCC in Metastatic Colorectal Cancer: A Nonrandomized Clinical Trial
Source: JAMA Oncol. 2024 Sep 19;10(11):1532–6. doi: 10.1001/jamaoncol.2024.3891 (PMC11413756; doi:10.1001/jamaoncol.2024.3891)
Supplement: Supplement 2. — Trial protocol [file jamaoncol-e243891-s002.pdf]

Study Protocol

Safety and efficacy of a new generation of CAR-T cell therapy targeting colorectal cancer

**Protocol No.:**	ICT-CAR-CRC
**Sponsor:**	Innovative Cellular Therapeutics Co., Ltd.
**Sponsor's contact information**	Building 1, 6055 Jinhai Road, Fengxian District, Shanghai
**Postal code:**	201499
**Tel:**	86-21-58950719
**Fax:**	86-21-58950719

**Version:**	**1.3.1**
**Date:**	**March 18, 2020**

Study: In accordance with the ethical principles of *the Declaration of Helsinki,* and the ethical principles consistent with *the International Conference on Harmonization Good Clinical Practice* and the requirements of applicable regulations.

The statistical analysis plan are described in detail in Section 10 (Precautions for statistical analysis)

Informed Consent Page of Investigator Protocol

Signature page for the approval of the protocol

I have carefully read the protocol in detail and agree to conduct the study in accordance with the protocol, *the International Conference on Harmonization of Technical Requirements for Good Clinical Practice (ICH GCP),* and *the Helsinki Declaration* with the approval of the Ethics Committee and the written consent of the subjects. Signing this form indicates that the investigator has signed a written agreement with Innovative Cellular Therapeutics Co., Ltd. and its representatives.

Principle investigator

_ _

Name and title: Date:

Institution:

Sponsor representative

_ _

Name, title: Date

Innovative Cellular Therapeutics Co., Ltd.

Tel: 86-21-58950719

Fax: 86-21-58950719

Protocol review

**Title**	Safety and efficacy of a new generation of CAR-T cell therapy targeting colorectal cancer
**Indications**	Advanced colorectal cancer
**Study design**	The ICT-CAR-CRC study is an open-labeled and single-center study to evaluate the safety and efficacy of targeted CAR-T therapy in the patients with advanced colorectal cancer<br>Twelve adult subjects with advanced colorectal cancer will be enrolled. Each subject shall follow the treatment protocol and procedure requirements of this study. Each subject is required to complete the following study periods:<br>1. Screening period<br>2. Preconditioning chemotherapy period (as required)<br>3. Treatment period<br>4. Post-treatment evaluation period<br>5. Long-term follow-up period<br>The requirements for each period of the study are detailed in Section 7.
**Indications**	This study aims to evaluate the safety, tolerance and efficacy of targeted CAR-T therapy for the treatment of colorectal cancer.
**Primary endpoint**	The primary efficacy endpoint of this study is safety and the objective response rate (complete response [CR] rate + partial response [PR] rate) of targeted CAR-T therapy in the patients with advanced colorectal cancer. The efficacy evaluation shall refer to RECIST1.1.
**Secondary endpoints**	1. Duration of response<br>2. Progression-free survival time<br>3. Overall survival<br>4. Incidence rate of adverse events and changes in clinically significant laboratory safety indicators<br>5. Incidence rate of anti-targeted CAR-T antibodies<br>6. Anti-targeted CAR + T-cell level in blood<br>7. Cytokine levels in serum<br>8. VAS score < 4 points
**Exploratory**	The developments of potential biomarkers will be investigated based

**endpoint**	on the evaluations of the proposed effects of blood cells, tumor cells and investigational product
**Sample size**	Twelve adult subjects with advanced colorectal cancer
**Inclusion criteria of study subjects**	1. Patients aged between 18 and 70 years old;<br>2. Patients with GUCY2C expression level≥ 1+ as determined by the laboratory test which is approved by the sponsor using the IHC method;<br>3. Patients who suffer from colorectal cancer as confirmed by pathology test;<br>4. Patients for whom the surgery is not available or inapplicable, or patients with recurrence after surgery;<br>5. Patients with at least one extracranial measurable lesion as stipulated by the RECIST 1.1.;<br>6. Patients with an expected survival time≥ 90 days;<br>7. Patients with normal functions of main organs, i.e., meeting the following criteria:<br>1) ECOG score for the evaluation of physical conditions shall be 0-1 point, or KPS score >70;<br>2) Blood routine test shall meet the following criteria: HB≥90 g/L (no blood transfusion within 14 days), ANC≥ 1.5* 10^9/L, PLT≥80* 10^9/L, Alb ≥ 2.8 g/dL, serum lipase and amylase < 1.5* ULN (upper limit of normal range);<br>3) Biochemical test shall meet the following criteria: TBIL ≤ 1.5* ULN (upper limit of normal range); ALT and AST ≤2.5* ULN. In case of liver metastasis, ALT and AST ≤5* ULN; Serum Cr ≤1* ULN, endogenous creatinine clearance rate > 50 ml/min (Cockcroft-Gault formula);<br>4) Cardiac ejection fraction > 55%;<br>8. Patients without any bleeding diseases or coagulation dysfunctions;<br>9. Patients without an allergy to the contrast agent;<br>10. Women of childbearing age shall undergo a pregnancy test (serum or urine) within 7 days prior to the enrollment. The test result should be negative, and they are willing to use appropriate contraceptive methods

	during the trial and 8 weeks after the last administration of CAR-T cells (women who have received sterilization surgery or are at least 2 years post-menopause shall be considered as infertile);<br>11. The subjects shall voluntarily participate in the study and sign the informed consent forms, with good compliances and good cooperation during the follow-up visits.
**Exclusion criteria**	1. Patients with T-cell transduction efficiency $< 10\%$ or T-cell amplification less than 5 times after culture;<br>2. Pregnant or lactating women;<br>3. Patients who have participated in clinical trials of other drug within 4 weeks prior to the study;<br>4. Patients with hypertension which cannot be well controlled by a single antihypertensive drug (systolic blood pressure > 140 mmHg, diastolic blood pressure > 90 mmHg, as judged by the investigator appropriately), with myocardial ischemia or myocardial infarction above grade I, arrhythmias above grade I (including QT interval$\geq$ 440ms), or cardiac insufficiency;<br>5. Patients with unhealed wounds or fractures in the chest or other parts for a long time;<br>6. Patients who have a medical history of psychotropic drug abuses and can not give up such psychotropic drug or have a medical history of mental disorders;<br>7. Patients with objective evidence of previous and current history of pulmonary fibrosis, interstitial pneumonia, pneumoconiosis, radiation pneumonia, drug-related pneumonia, and severe pulmonary function impairment;<br>8. Patients infected with fungi, bacteria, viruses or others that cannot be controlled or are required to be treated with antibiotic. Simple urinary tract infection and uncomplicated bacterial pharyngitis are allowed subject to the approval of medical monitoring;<br>9. According to the NCI-CTCAE 4.0 standard, the subjects who have been previously treated with chemotherapy present hematological toxicity $\geq$ grade 2 or nonhematological toxicity $\geq$ grade 3;

	10. Patients with a known history of HIV, or those tested to be positive during nucleic acid tests of hepatitis B (HBsAg positive) or hepatitis C virus (anti HCV positive);<br>11. Patients indwelled with a catheter or a drainage tube (e.g., a bile drainage tube or a pleural/peritoneal/pericardial catheter). Special peripherally inserted central catheters (whether the fistulation, percutaneous nephrostomy tube and indwelled Foley catheter affect the patients with colorectal cancer shall be considered by the researcher) are allowed;<br>12. Patients with brain metastasis;<br>13. Patients with a history of CNS disease or those who suffer from CNS diseases, such as seizure disease, cerebral ischemia/hemorrhage, dementia, cerebellar disease, or any autoimmune disease related to CNS;<br>14. Patients with a major immune deficiency;<br>15. Patients with a history of severe hypersensitivity to main therapeutic drugs in this study (including fludarabine, cyclophosphamide, MESNA, tocilizumab for the prevention and treatment of CRS and anti-infective drugs used during preconditioning treatment);<br>16. Patients with a history of deep venous thrombosis or pulmonary embolism 6 months prior to the enrollment;<br>17. Patients with a history of autoimmune diseases (e.g., Crohn's disease, rheumatoid arthritis, and systemic lupus erythematosus) resulting in organ injury or requiring systemic immunosuppressive/systemic disease regulating drugs in the past 2 years;<br>18. Patients with any disease that may interfere with the safety or efficacy evaluation of the study therapy.<br>19. Female subjects who are unwilling to take contraceptive measures from the signing of the informed consent form to 6 months after the completion of CAR-T administration.
**Treatment protocol**	Investigational product:<br>CAR-transduced autologous T cells at a dose of 1* 10^5-1* 10^7/kg.<br>Remarks:

	Target + CD19 + IL6 + IL12 + IFN-r<br>Preconditioning chemotherapy:<br>Targeted CAR-T cells will be administered after the patient has been treated with the preconditioning chemotherapy regimen consisting of fludarabine 30 mg/m^2/d and cyclophosphamide 300 mg/m^2/d for 1 day. Refer to Section 6 for details of chemotherapy.<br>The feasibility of preconditioning chemotherapy is determined according to the patient's physical condition, nutrition and willingness, and the consultation opinions shall be followed. The chemotherapy regimen may be adjusted as appropriate.
**Study process**	At the specific time points stipulated in the subjective-objective-assessment-plan, the subjects will participate in the following procedures: collections of informed consent forms, general medical history (including previous treatment of colorectal cancer), physical examination (including vital signs and ECOG), neurological evaluation, blood collection for whole blood cell count (CBC), biochemical test, cytokine test, C-reactive protein test, lymphocyte subset test, and analysis of anti-targeted GUCY2CAR antibody and anti-GUCY2CAR + T cell.<br>Women of childbearing age will take a urine or serum pregnancy test. Subjects will also receive electrocardiogram (ECG), echocardiography (ECHO), magnetic resonance imaging (MRI) of the brain, and positron emission tomography computed tomography (PET-CT).<br>Throughout the study, the subjects are required to report concomitant medications and adverse events regularly, and their illness will be evaluated.
**Statistical analysis**	The statistical analysis of this study will mainly include the modified intention to treat (MIT) population, including all the subjects treated with the targeted CAR-T therapy at the target dose.<br>In general, continuous variables (such as age) will be expressed by the number of observations, mean, median, standard deviation, minimum and maximum; and categorical variables will be expressed by the

	frequency and percentage of each category.<br>Safety will be evaluated through a summary of adverse events, and changes in laboratory test results and vital signs. The MedDRA will be used to code adverse events. The number and frequency of adverse events will be summarized using the corresponding terms according to the system organ classification (SOC). The cytokine release syndrome (CRS) will be analyzed according to its severity and system organ classification. All the adverse events will be graded according to the NCI CTCAE (version 4.03). All the SAEs (including death) and DLT will be tabulated and summarized separately.

Terminology of the study

Abbreviation or term	Definition/interpretation
AE	Adverse event
ANC	Absolute neutrophil count
CAR	Chimeric antigen receptor
CAR+	Positive chimeric antigen receptor
CBC	Complete blood cell count
CMV	Cytomegalovirus
CNS	Central nervous system
CPF	Cell processing mechanism
CR	Complete remission
CRF	Case report form
CRS	Cytokine release syndrome
CSF	Cerebrospinal fluid
CTCAE	Common Terminology Criteria for Adverse Events
DLT	Dose limiting toxicity
DVT	Deep venous thrombosis
eACT™	Engineering autologous cell therapy
EBV	Ehrlich Barr virus
ECHO	Echocardiography
ECG	Electrocardiogram
ECOG	Electrocardiogram

Individual subject study	Defined as an individual subject to receive the evaluation at
End of the study (end of the preliminary research)	Defined as the last subject to receive the evaluation or intervention to achieve the final data collection purpose, the primary endpoint at month 6
End of study (end of the trial)	Defined as the last subject to receive the evaluation or intervention to complete the study evaluation, including survival evaluation
FAS	Full analysis set
HABA	Human anti-bovine antibody
HAMA	Human anti-mouse antibody

Abbreviations or terms	Definition/interpretation
HLH	Hemophilic lymphohistiocytosis
ICF	Informed consent form
ICU	Intensive care unit
IP	Investigational product
IRB/IEC	Institutional Review Committee/Independent Ethics Committee
IWG	International Working Group
Targeted GUCY2C CAR-T	Autologous T cells containing GUCY2C chimeric antigen receptor which are transfected with virus vector
LMWH	Low molecular weight heparin
LTFU	Long-term follow-up
mITT	Modified intention to treat
MMSE	Mini-mental state examination
MRI	Magnetic resonance imaging
MSGV1	Murine stem cell virus based retroviral vector 1
NaCl	Sodium chloride
OS	Overall survival
PET-CT	Positron emission tomography- computed tomography
PBMC	Peripheral blood mononuclear cells
PD	Disease progression

PR	Partial response
scFv	Single chain antibody variable region fragments
SOA	Subjective-objective-assessment-plan
SD	Stable disease
SUSAR	Suspected unexpected serious adverse reactions
Day 0 of the study	Defined as the day when the subject is transfused with targeted CAR-T
TEAE	Treatment emergent adverse event
GUCY2C	Guanylate cyclase C
ULN	Upper limit of normal range

Study protocol

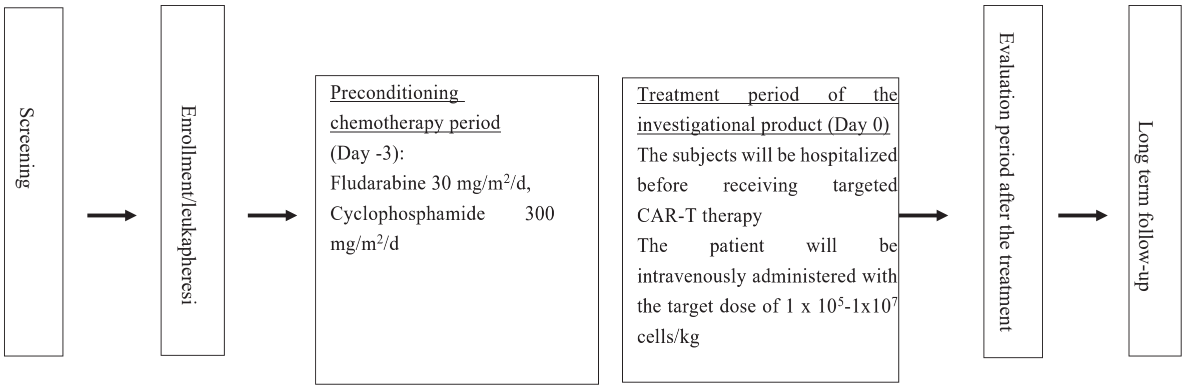

Each subject will abide by the same study protocol and the requirements for procedures. Each subject will be followed up in the following periods of the study: screening period, enrollment/leukapheresis period, preconditioning chemotherapy period, investigational product treatment period, posttreatment evaluation period and long-term follow-up period.

Contents

Protocol review 3
Terminology of the study 8
Study protocol 11
1 Objective 14
2 Background of the illness and basis 14
2.1 Epidemiological characteristics 14
2.2 Advanced colorectal cancer 14
2.3 Basis for the study 14
3 Study design 19
3.1 Overall study design 19
3.2 Participating center 19
3.3 Number of subjects 19
3.4 Replacements of the subjects 19
3.5 Duration of the study 19
4. Screening and enrollment of the subjects 20
5 Eligibility of the subjects 20
5.1 Diagnosis criteria 20
5.2 Inclusion criteria 20
5.3 Exclusion criteria 21
6 Study therapy regimen 22
6.1 Therapeutic terminology 22
6.2 Study therapy 23
6.3 Study therapy plan 25
6.4 Toxicity management 28
7 Study procedures 38
7.1 Informed consent form 38
7.2 Demographic data 39
7.3 Medication history and medical history 39
7.4 Physical examination, vital signs and physical conditions 39
7.5 Nervous system evaluation 40

7.6 Heart function 40
7.7 Magnetic resonance imaging 40
7.8 Bone marrow biopsy 40
7.9 Disease remission evaluation 41
7.10 Laboratory 42
7.11 Biomarkers 43
7.12 Descriptions of study periods 43
8 Withdrawal of the subjects 55
8.1 Reasons for discontinuation of the treatment 55
8.2 Reasons for exclusion from the study 56
9 Safety report 56
9.1 Adverse events 56
9.2 Reporting of adverse events 57
9.3 Definition of serious adverse events 58
9.4 Reporting of severe adverse events and ≥ grade 3 non severe CRS events and above 58
9.5 Pregnancy and lactation 59
9.6 Criteria for the discontinuation of enrollment 59
10 Precautions for statistical analysis 60
10.1 General principles 60
10.2 Analysis dataset 60
10.3 Study endpoint 60
10.4 Statistical analysis method 61
11 Regulatory obligations 62
11.1 Confidentiality for the subjects 62
11.2 Responsibilities of subjects to affix their signatures 63
12 Amendments and termination of the protocol 63
13 Study documents and retentions 63
14 Study monitoring and data collection 64
15 Publication 64
References 64

1 Objective

This study aims to evaluate the safety, tolerance and efficacy of targeted CAR-T therapy for the treatment of colorectal cancer.

2 Background of the illness and basis

2.1 Epidemiological characteristics

Colorectal cancer (CRC) is the third most common malignant tumor worldwide. The incidence rate of colorectal cancer varies greatly in different regions of the world, with the highest incidence rate in North America and Oceania, followed by Europe, and the lowest incidence rate in Asia and Africa. Monitoring data have shown that the incidence rates of CRC in males and females in the US have decreased in recent decades. From 1976 to 2005, the incidence rates of malignant tumors in the proximal colon, terminal colon and rectum decreased from 22.5/100,000 and 18.8/100,000 19.2/100,000 to 46.4/100,000, 11.7/100,000 and 13.6/100,000, respectively. Statistics in 2009 showed that approximately 106,100 patients were newly diagnosed with colon cancer in the United States. The incidence rate and mortality rate of CRC in men and women accounted for 10% and 9% of the total patients with common tumors in that year, respectively, ranking third in terms of the incidence rate and mortality rate of common tumors in that year.

2.2 Advanced colorectal cancer

The incidence rate and mortality rate of colorectal cancer in China are on the rise. The incidence rate and mortality rate of colorectal cancer were 23.03/100,000 and 11.11/100,000 in 2011, respectively. Cancer statistics in China in 2015 showed that the incidence rate and mortality rate of colorectal cancer in China ranked fifth among all malignant tumors, including 376,000 new patients and 191,000 deaths. The incidence and mortality rates of patients with colon cancer in urban areas are far higher than those in rural areas, and the incidence rate of colorectal cancer is increasing significantly. Most patients are found to be in the middle and late stages of colorectal cancer when diagnosed.

2.3 Basis for the study

Most advanced cancers will eventually progress to refractory diseases that have poor responses to conventional therapy, so they require new modes of treatment. Immunotherapy is a very promising method for the treatment of many types of cancers by enhancing antitumor-based immune responses. T cells play an important role in the destruction of diseased cells throughout the body. Studies on immune checkpoint inhibitors and tumor-infiltrating lymphocytes have shown the potential of T cells for the treatment of cancers. T cells are effective only if they are tumor specific, sufficient in number, and overcome any local immunosuppressive factors. Programmed T cells are a very promising therapy for the treatment of cancers

(Kershaw 2013).

Engineering autologous cell therapy (eACT™) is a process in which the patients' own T cells aggregate and are changed genetically later and then recognize and target the antigens expressed on the surfaces of specific malignant tumor cells (Kochenderfer, 2013). Human T cells will be genetically programmed to mediate the treatment of tumors in patients, which has been proven to be effective in many studies and makes it possible to treat various types of cancers.

2.3.1 Introduction of targets

2.3.1.1 Expression of GUCY2C

Guanylate cyclase C (GUCY2C) is a membrane-bound receptor that produces the second messenger cGMP after the activation of the hormone ligand guanylate or uroguanosine and regulates intestinal homeostasis, tumorigenesis and obesity. GUCY2C is mainly expressed in the apical membrane of intestinal epithelial cells and is separated from the systemic compartment through the intestinal barrier. Similarly, it is also expressed in hypothalamic neurons, mediates a new intestinal endocrine axis regulating appetite and is separated from the systemic compartment through the blood–brain barrier. More significantly, GUCY2C is a cancer mucosal antigen that is generally overexpressed in primary and metastatic human colorectal cancers.

Studies have shown that CAR-T cells targeting mouse GUCY2C are effective against colorectal cancer, which has metastasized to the lungs, while producing no intestinal toxicity in syngeneic immunoactive mouse models (Magee MS, 2016). Similarly, other GUCY2C-targeted therapeutic agents, including antibody–drug conjugates (Marszalowicz GP, 2014) and vaccines (Snook AE, 2009; Snook AE, 2014), are safe in preclinical animal models, and the therapeutic regimens using these models are now used for metastatic esophageal, gastric, pancreatic and colorectal cancers (NCT02202759, NCT02202785, NCT01972737). The safety of these treatment regimens, against the background of GUCY2C expression across the intestinal root-caudal axis, reflects the compartmentalized expression of GUCY2C, which is most commonly expressed in the apical membrane but demonstrates limited expression in the basolateral membranes of epithelial cells. Systemic radiolabeled imaging agents bound to GUCY2C ligands target metastasis expressing GUCY2C but do not recognize intestinal epithelial cells, confirming the mucosal compartmentalization of the receptor. These characteristics suggest that GUCY2C may be an effective therapeutic target for metastatic colorectal cancer.

2.3.1.2 CD19 and its expression

CD19 is a 95 kD transmembrane protein expressed only in the B-cell line. CD19 is expressed in all normal B cells, from the previous B-cell stage to the final differentiation stage, and is not expressed in pluripotent hematopoietic stem cells or most plasma cells. CD19 expression types are found in B-cell malignancies, including all subtypes of B-cell NHL, chronic lymphoblastic leukemia (CLL), and non-T-cell acute lymphoblastic leukemia (ALL) (Blanc 2011), except multiple myeloma.

2.3.2 Introduction to IL12

IL-12 is one of the important cytokines triggering the cell-mediated immune response. It is mainly produced by Toll-like receptor-activated antigen-presenting cells and interacts with CD4+ T cells 1 (Dorman SE, 2000).

IL-12 stimulates the growth and functions of activated NK cells and CD8+ and CD4+ T cells, changes the differentiation of CD4+ Th0 cells into the Th1 phenotype, enhances antibody-dependent cytotoxicity (ADCC) against tumor cells, induces IgG and inhibits the production of IgE by B cells, stimulates T cells and natural killer (NK) cells to produce interferon-γ (IFN-γ) and tumor necrosis factor-α (TNF-α), and alleviates IL-4-mediated IFN-γ inhibition. In addition, IL-12 enhances the cytotoxic activities of NK cells and CD8+ T cells.

IL-12 also has antiangiogenic activity, which means that it prevents the formation of new blood vessels by increasing the production of IFN-γ, which, in turn, increases the production of chemokines, known as inducible protein-10 (IP-10 or CXCL10), to mediate this antiangiogenic effect 2 (Voest e, 1995).

In conclusion, IL-12 has various biological characteristics and controls the effects of immune effectors against a variety of malignant tumors, so it is recognized as the center of anticancer immunotherapeutic agents.

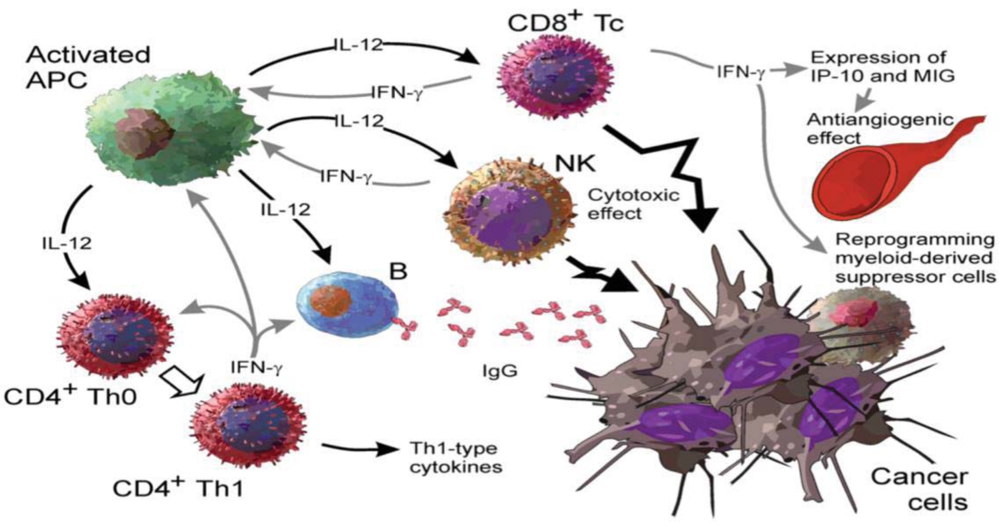

Figure 1 Overview of the antitumor biological characteristics of IL-12 (LASEK W, 2014)

2.3.3 Introductions to IL6 and IFNγ

IL-6 is a soluble mediator with pleiotropic effects against inflammation, immune response and hematopoiesis. IL-6 promotes the synthesis of acute phase reactive proteins, such as CRP and serum amyloid A, and stimulates the production of antibodies and the development of effector T cells.

In addition, IL-6 promotes the specific differentiation of naïve CD4+ T cells, which bind to transforming growth factor (TGF)-β to promote naïve CD4+ T-cell differentiation into Th17+ cells, but IL-6 also inhibits TGF-β-induced Treg differentiation (Bettelli et al. 2006). Upregulation of Th17/Treg is considered to disrupt immune tolerance, thus resulting in pathologically autoimmune and chronic inflammatory diseases (Kimura and Kishimoto 2010).

Further studies have shown that IL-6 promotes the differentiation of T follicular helper cells and the production of IL-21 (MA et al. 2012). IL-21 specifically regulates the synthesis of immunoglobulin (Ig) and the production of IgG4. IL-6 also induces CD8+ T cells to differentiate into cytotoxic T cells. BSF-2 (B-cell stimulation factor 2) induces the differentiation of activated B cells into plasma cells that produce antibodies and thereby produce antibodies continuously.

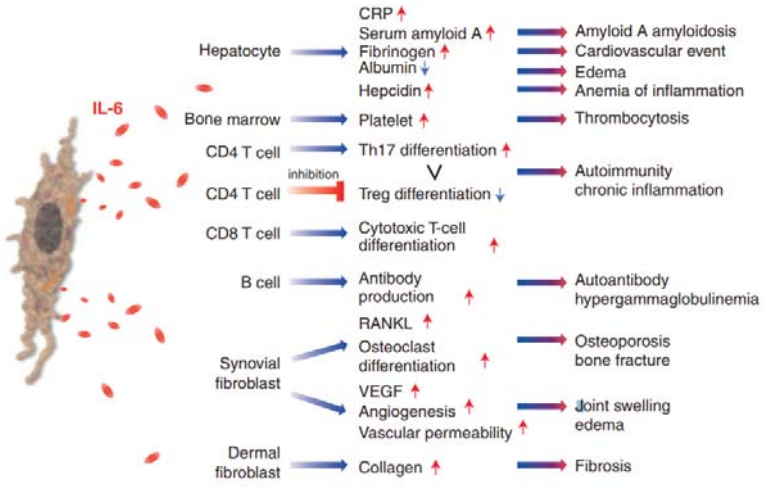

Figure 2 Roles of IL-6 in inflammation, immunity and diseases (Heinrich et al. 1990)

IFNγ or type II interferon is a cytokine essential for innate and adaptive immunity against viruses, some bacteria and protozoan infection 3 (Schroder et al., 2004). IFNγ is an important activator of inducers of the expression of macrophages and class II major histocompatibility complex (MHC) molecules. IFN-γ is secreted by NK cells and T cells and has antiviral, immunoregulatory and antitumor effects. IFNγ is the most effective mediator of IL-12.

Many studies have proven that IFNγ inhibits cell growth and triggers apoptosis, induces the autophagy of macrophages and tumor cells 4-6 (Li et al., 2012; Matsuzawa et al., 2012, 2014), and induces more tumor antigen-presenting cells, thereby enhancing the immune response to the tumor microenvironment. In turn, IFNγ causes tumor cells to be more sensitive to effectors, such as T cells and NK cells, resulting in the death of tumor cells; promotes the polarization of T cells and macrophages 7 (Biswas and Mantovani, 2012); and induces the expression of CD40 in other antigen-presenting cells, i.e., DCs, which will migrate to the nearest lymph node to present antigen to naïve T cells, thus activating the adaptive immune system 8 (Delves, 2011).

2.3.4 Targeted CAR-T-cell products

CAR-T cells responding to GUCY2C will be prepared using mature T-cell induction and transformation technology and will be recognized as targeted GUCY2C CAR+ cells by flow cytometry.

CAR molecules will be constructed with human-derived CD19 antibody to avoid the influence of

human anti-mouse antibody response on the function of mouse antibody-derived CAR-T cells, enabling them to better perform their functions and to prolong their duration.

3 Study design

3.1 Overall study design

Adult subjects with advanced colorectal cancers will be enrolled in this study. Each subject will abide by the requirements of the same trial therapy protocol and procedures. Each subject is required to complete the following stages of the study:

Screening period

Enrollment/leukapheresis period

Conditioning chemotherapy period

Treatment period with the investigational product (IP)

Post-treatment evaluation period

Long-term follow-up period

The study requirements for each stage are detailed in Section 7.

3.2 Participating center

This is a single-center clinical trial.

3.3 Number of subjects

The participants enrolled in this trial are referred to as the "subjects". Please note that the sponsor may choose to terminate the enrollment at any time.

3.4 Replacements of the subjects

The subjects will continue to be enrolled in the study until the number of subjects included in the mITT set has been met. The subjects who have not been administered CAR-T cells at the target dose will be included in the safety analysis for disposals as appropriate (refer to Section 10.5).

3.5 Duration of the study

3.5.1 Study duration of individual subjects

The study durations of individual subjects will differ. It will take approximately five years to complete the study protocol, from the signing of the informed consent form to the end of the long-term follow-up period. However, the study duration of individuals may vary depending on the screening requirements, responses to the treatment, and survival time of the subjects.

3.5.2 Completion of the study

Completion of the study is defined as the date on which the last subject has completed the long-term follow-up visit, has been lost to the follow-up visit, has withdrawn the informed consent or dies. When all the subjects enrolled in the study complete the 6-month disease response evaluation (except for those who were lost to follow-up, withdrew informed consent and died), the primary efficacy evaluation will be performed.

4. Screening and enrollment of the subjects

All the subjects are required to sign the informed consent forms approved by the IRB/IEC and indicate the date prior to any specific study procedure or activity that is not a part of the daily care.

Refer to Section 7 for details.

Each subject included in the screening period will be assigned a unique subject identification number prior to any specific study procedure or activity. This number is used to distinguish the subject throughout the study and will be recorded in all the study documents related to the subject. In addition, the subject identification number will remain unchanged throughout the clinical study and will not be changed after enrollment, rescreening or retreatment.

5 Eligibility of the subjects

5.1 Diagnosis criteria

The diagnosis is made according to *the Tumor Diagnostics (6th Edition)* and *the Internal Medicine (6th Edition)* published by the People's Health Publishing House of the People's Republic of China.

5.2 Inclusion criteria

1. Patients aged between 18 and 70 years old;
2. Patients with GUCY2C expression levels ≥ 1+ as determined by the laboratory test, which is approved by the sponsor using the IHC method;
3. Patients who suffer from colorectal cancer as confirmed by pathology test;
4. Patients for whom surgery is unable or inapplicable to perform or patients with recurrence after surgery;
5. Patients with at least one extracranial measurable lesion as stipulated by RECIST 1.1.
6. Patients with an expected survival time ≥ 90 days;
7. The patients with normal functions of the main organs met the following criteria:

1) ECOG score for the evaluation of physical conditions shall be 0-1 points, or KPS score >70;

2) Routine blood tests met the following criteria: HB≥90 g/L (no blood transfusion within 14 days), ANC≥ 1.5* 10^9/L, PLT≥80* 10^9/L, Alb ≥ 2.8 g/dL, serum lipase and amylase < 1.5* ULN (upper limit of normal range);

3) Biochemical tests met the following criteria: TBIL ≤ 1.5* ULN (upper limit of normal range); ALT and AST ≤2.5* ULN. In cases of liver metastasis, ALT and AST ≤5* ULN; Serum Cr ≤1* ULN, endogenous creatinine clearance rate > 50 ml/min (Cockcroft-Gault formula);

4) Cardiac ejection fraction > 55%;

8. Patients without any bleeding diseases or coagulation dysfunctions;

9. Patients without allergies to the contrast agent;

10. Women of childbearing age shall undergo a pregnancy test (serum or urine) within 7 days prior to enrollment. The test result shall be negative, and they are willing to use appropriate contraceptive methods during the trial and 8 weeks after the last administration with CAR-T cells (the women who have received sterilization surgery or are at least 2 years postmenopause shall be considered infertile);

11. The subjects voluntarily participate in the study and sign the informed consent forms, with good compliance and good cooperation during the follow-up visits.

5.3 Exclusion criteria

1. Patients with T-cell transduction efficiency < 10% or T-cell amplification less than 5 times after culture;

2. Pregnant or lactating women;

3. Patients who participated in the clinical trials of other drugs within 4 weeks prior to the study;

4. Patients with hypertension that cannot be well controlled by a single antihypertensive drug (systolic blood pressure > 140 mmHg, diastolic blood pressure > 90 mmHg, as judged by the investigator appropriately), with myocardial ischemia or myocardial infarction above grade I, arrhythmias above grade I (including QT interval ≥ 440 ms), or cardiac insufficiency;

5. Patients with unhealed wounds or fractures in the chest or other parts for a long time;

6. Patients who have a medical history of psychotropic drug abuse and cannot give up such psychotropic drugs or have a medical history of mental disorders;

7. Patients with objective evidence of previous and current history of pulmonary fibrosis, interstitial pneumonia, pneumoconiosis, radiation pneumonia, drug-related pneumonia, and severe pulmonary function impairment;

8. Patients infected with fungi, bacteria, viruses or others that cannot be controlled or are required to be

treated with antibiotics. Simple urinary tract infection and uncomplicated bacterial pharyngitis are allowed subject to the approval of medical monitoring;

9. According to the NCI-CTCAE 4.0 standard, subjects who have been previously treated with chemotherapy present hematological toxicity ≥ grade 2 or nonhematological toxicity ≥ grade 3;

10. Patients with a known history of HIV or those who tested positive during nucleic acid tests for hepatitis B (HBsAg positive) or hepatitis C virus (anti-HCV positive);

11. Patients indwelled with a catheter or a drainage tube (e.g., a bile drainage tube or a pleural/peritoneal/pericardial catheter). Special peripherally inserted central catheters (whether the fistulation, percutaneous nephrostomy tube and indwelling Foley catheter affect patients with colorectal cancer should be considered by the researcher) are allowed;

12. Patients with brain metastasis;

13. Patients with a history of CNS disease or those who suffer from CNS diseases, such as seizure disease, cerebral ischemia/hemorrhage, dementia, cerebellar disease, or any autoimmune disease related to CNS;

14. Patients with a major immune deficiency;

15. Patients with a history of severe hypersensitivity to the main therapeutic drugs in this study (including fludarabine, cyclophosphamide, MESNA, tocilizumab for the prevention and treatment of CRS and anti-infective drugs used during preconditioning treatment);

16. Patients with a history of deep venous thrombosis or pulmonary embolism 6 months prior to enrollment;

17. Patients with a history of autoimmune diseases (e.g., Crohn's disease, rheumatoid arthritis, and systemic lupus erythematosus) resulting in organ injury or requiring systemic immunosuppressive/systemic disease regulating drugs in the past 2 years;

18. Patients with any disease that may interfere with the safety or efficacy evaluation of the study therapy.

19. Female subjects who are unwilling to take contraceptive measures from the signing of the informed consent form to 6 months after the completion of CAR-T administration.

6 Study therapy regimen

6.1 Therapeutic terminology

The following terms are used to describe and define the treatment regimen:

- The drugs used in the conditioning chemotherapy regimen of this study were fludarabine and cyclophosphamide.
- The investigational product used in this study is known as targeted CAR + T.

- Trial therapy refers to the therapy required by the protocol.

6.2 Study therapy

6.2.1 Preconditioning chemotherapy

Unless otherwise specified, conditioning chemotherapy will be conducted by the study center. For the packaging, storage, preparation, administration and toxicity management of the chemotherapy drugs, please refer to the current label.

6.2.1.1 Fludarabine

Fludarabine phosphate is a synthetic purine nucleoside that is different from physiological nucleotides. It is made up of arabinose as the sugar molecule instead of ribose or deoxyribose. Fludarabine is an antimetabolite of purine antagonists.

For detailed use of fludarabine, please refer to the latest version of the packaging inserts.

6.2.1.2 Cyclophosphamide

Cyclophosphamide is a nitrogen shallard-derived alkylating agent. Cyclophosphamide acts as an active metabolite after it is converted into active metabolites in the liver, and the drug also has strong immunosuppressive activity. The serum half-life of the drug administered intravenously ranges from 3 to 12 hours. The drug and/or its metabolites will still be detected in the serum 72 hours after administration.

For detailed use of cyclophosphamide, please refer to the latest version of the packaging inserts.

6.2.1.3 Mesna

Mesna is an antidote to treat chemotherapy-induced hemorrhagic cystitis. The active constituent of mesna is a synthetic sulfhydryl compound known as sodium-2-mercaptoethane sulfonic acid, and its molecular formula is $C2H5NaO3S2$.

Mesna shall be administered according to the medication guidelines. For detailed use of mesna, please refer to the latest version of the packaging inserts.

6.2.2 Targeted CAR-T

Targeted CAR-T therapy may be administered with fresh CAR-T cells or cryopreserved CAR-T cells.

In the case of infusion with fresh CAR-T cells, targeted CAR-T cells will be stored in a special bag. The product stored in the bag is slightly turbid and is milky white to yellow. It will be transported to the hospital by a professional third-party company.

In the case of infusion with cryopreserved CAR-T cells, targeted CAR-T cells will be cryopreserved in a cryopreservation bag. The product in the bag will be slightly turbid, in milky to yellow colors. The cryopreservation bags containing targeted CAR-T cells shall be stored in a dry liquid nitrogen conveyer and cryopreserved when they arrived. The bags shall be stored in the gas phase of liquid nitrogen, and the product will be kept cryopreserved until it is ready for treatment to guarantee that the living autologous cells will be used in the subjects. Several inactive substances will be added to the cryopreserved product to guarantee the survival rates and stability of living cells during cryopreservation, thawing and transfusion.

Targeted CAR-T product is a subject-specific product, and we will identify the subject by his or her unique ID number. It is of great importance to confirm that the product and the subject-specific label match the subject's information (e.g., the subject's initials and ID number) upon receipt. If the subject-specific label does not match the expected subject, the targeted CAR-T should not be transfused. The handover of targeted CAR-T cells, the start/end time of thawing, and the start/end time of administration shall be recorded in the subjects' medical records. The product should not be thawed before the subject is ready for transfusion.

6.2.3 Concomitant therapy

During the study period, the investigator may prescribe any concomitant drugs or therapy he or she deems necessary to provide adequate supportive care, except for those listed in the medication table of Section 6.2.4.

From the date of the informed consent form to 3 months after targeted CAR-T therapy, all concomitant therapies, including drugs, intubation, dialysis and blood products, will be recorded. All the targeted drugs, including gamma globulin, immunosuppressive drugs, anti-infective drugs and vaccination, will be recorded from the end of the 3-month follow-up period to 2 years after disease progression. For the subjects who have been enrolled in the study but have not been administered targeted CAR-T therapy, the

concomitant therapies (e.g., leukapheresis and conditioning chemotherapy) shall be recorded from the date of the informed consent forms to 30 days after the last procedure. For the subjects who have not been enrolled in the study (e.g., those who have failed to meet the screening criteria or have not undergone leukapheresis), concomitant therapy associated with any serious adverse events shall be recorded.

6.2.4 Prohibited drugs

Corticosteroids at the pharmacological dose (prednisone at a dose ≥5 mg/d or other corticosteroids at equivalent doses) and other immunosuppressive drugs should be prohibited 7 days prior to leukapheresis and 5 days prior to targeted CAR-T therapy.

Corticosteroids and other immunosuppressive drugs should also be prohibited 3 months after targeted CAR-T therapy unless they are used to manage the toxicity associated with targeted CAR-T therapy. Other drugs that may interfere with the evaluations of the investigational product, such as nonsteroidal anti-inflammatory drugs, should also be prohibited during the same period, unless medically necessary.

The therapies for the treatment of colorectal cancer, such as chemotherapy, immunotherapy, targeted therapeutic drugs, radiotherapy, high-dose corticosteroids (except those defined/permitted in this protocol) and other experimental preparations, are prohibited, except those required for the treatment of disease progression after the transfusion with targeted CAR T cells.

If you have any questions about the use of a particular drug/therapy, please consult the medical monitor.

6.2.5 Follow-up treatment

The follow-up treatments after the transfusion with targeted CAR T cells for the treatment of the subject's illness, such as nonstudy-specific chemotherapy, immunotherapy, targeted therapeutic drugs, stem cell transplantation, and radiotherapy, will be recorded until the subject completes long-term follow-up visits, is considered lost to follow-up, withdraws informed consent or dies.

6.3 Study therapy plan

6.3.1 Leukapheresis (within 5 days after the confirmation of inclusion criteria)

The subjects will undergo leukapheresis to collect white blood cells that target CAR T cells. The target CAR T cells collected by the participating center using the leukapheresis method will be transported

to the cell processing facility (CPF) overnight. Once a subject has undergone leukapheresis, they will be considered for inclusion in this study.

Monocytes will be collected using the leukapheresis method (12-15 L of peripheral blood will be collected by the machine, aiming to collect approximately 5-10x109 monocytes). The cells collected by the leukapheresis method will be packaged and then transported to the cell processing facility.

T cells, a product of leukapheresis that exists in each subject, will be purified to eliminate the remaining PBMCs after they have been sent to the cell processing facility. Subsequently, T cells will be activated and amplified, transduced with viral vector, introduced with CAR genes T cells, and then further amplified and cryopreserved for the preparation of the investigational product. Once the product has passed the specific test release, it will be transported back to the processing facility. After conditioning chemotherapy, each subject will be administered with his or her own targeted CAR T cells.

6.3.2 Instructions on study therapy and chemotherapy

The subjects will be treated with the nonmyeloablative preconditioning chemotherapy regimen with cyclophosphamide and fludarabine to promote lymphocyte depletion and to create an optimal environment for the amplification of targeted CAR-T cells in vivo. The subjects will start preconditioning chemotherapy with cyclophosphamide and fludarabine on Day -3. Preconditioning chemotherapy may be performed in the clinic.

The subjects will be advised to drink more water during and 24 hours after chemotherapy. Generally, the subjects should drink enough water but should also be closely monitored at the same time to prevent the production of excessive body fluid.

Instructions on CAR-T therapy

All the subjects will be hospitalized for targeted CAR-T therapy and then observed for a cycle. The subjects will be hospitalized until Day 7 after the start of targeted CAR-T therapy. The subjects shall not be discharged from the hospital before the targeted CAR-T therapy-related nonhematological toxicity has been alleviated to ≤ grade 1 or baseline level. If the investigator believes appropriate, the subject may be discharged from the hospital even if he or she develops noncritical and clinically stable toxicity or the toxicity > grade 1 has been alleviated gradually (e.g., renal insufficiency).

If the subject develops persistent targeted CAR-T-related fever, hypotension, hypoxia, and persistent > grade 1 central neurotoxicity, or if the treatment investigator considers necessary, he or she will continue to be hospitalized.

6.3.3 Basis for administration of the study therapy drug and basis for the dose of the preconditioning chemotherapy

Conditioning chemotherapy at an increased dose is associated with the subjects' clinical responses to cell therapy (Dudley, 2008). Specifically, adequate lymphocyte depletion in a preclinical model is associated with the amplification and functioning of transiently transfected T cells. The severity and duration of lymphocyte depletion in a preclinical model are associated with the antitumor activity of adoptive tumor-specific CD8+ T cells (Gattinoni 2005). Lymphocyte depletion may play a role by removing the deposition of cytokines in transfected cells, eliminating regulatory T cells or enhancing the activation of antigens (Klebanoff 2005). The combined use of cyclophosphamide and fludarabine is an effective cell depletion regimen. Optimizing the doses of cyclophosphamide and fludarabine and alleviating the severity and shortening the duration of cell depletion may enhance the activity of targeted CAR-T cells.

6.3.4 Study therapy

In this study, some patients with good physical conditions and nutritional status underwent a 1-day preconditioning chemotherapy after the evaluation following MDT discussion and consultation:

- Intravenous infusion of 1 L of 0.9% NaCl before the administration of cyclophosphamide on the same day, and the following treatments will be performed;
- Cyclophosphamide 300 mg/m^2 will be administered intravenously for 60 minutes on Day -3, followed by the following therapy:
- Fludarabine 30 mg/m^2 will be administered intravenously for 30 minutes on Day -3, followed by the following therapy:
- Another 1 L of 0.9% NaCl will be administered when the intravenous infusion of fludarabine ends;
- MESNA (sodium 2-mercaptoethanesulfonate) will be coadministered according to the medication guidelines.

Investigational product:

CAR-transduced autologous T cells at a dose of 1* 10^5-1* 10^7/kg.

Notes:

Target + CD19 + IL6 + IL12 + IFN-r

Preconditioning chemotherapy:

Targeted CAR-T cells will be administered after the patient has been treated with the preconditioning chemotherapy regimen consisting of fludarabine 30 mg/m^2/d and cyclophosphamide 300 mg/m^2/d for 1 day. Refer to Section 6 for the details of chemotherapy.

The feasibility of preconditioning chemotherapy will be determined according to the patient's physical constitution, nutrition and willingness, and the consultation opinions should be followed. The chemotherapy regimen may be adjusted as appropriate.

To avoid the risk of increased infection induced by lower white blood cells following preconditioning chemotherapy, Human Granulocyte Colony-stimulating Factor Injection (Xinruibai, PEG-rhG-CSF) manufactured by Qilu Pharmaceutical should be used 24 hours after CAR-T cells infusion.

6.4 Toxicity management

6.4.1 Cytokine release syndrome

Cytokine release syndrome (CRS) is a syndrome associated with anti-clonal antibodies and adoptive cell therapy that are used to activate lymphocytes. This illness is due to the release of cytokines from antibody-targeted cells, the recruitment of immune effector cells to the tumor, and the activation of immune cells in this process. Various clinical signs and symptoms associated with CRS will be present during the release of cytokines, including abnormalities in the heart, gastrointestinal tract and laboratory parameters (coagulation, kidneys and liver), respiratory system, skin, blood vessels (hypotension), and systemic signs and symptoms (fever, chills, headache, discomfort, fatigue, joint pain, nausea and vomiting).

The objective for CRS management of anti-CAR-T cell therapy is to prevent adverse life-threatening lesions while maintaining the benefits of antitumor effects. For CRS grading, refer to the CRS severity scale related to antibody therapy published by NCI investigators. As this scale is also adopted in other therapies to define mild, moderate, severe and life-threatening events caused by overlapping symptoms and to provide guidelines on treatment recommendations, Lee et al. revised the CRS grading system, which is shown below (Lee 2014). The grading scale shown in Table 2 and the treatment guidelines shown in Table 3 will be used in this study.

Table 2 Cytokine release syndrome grading scale (excluding neurotoxicity)

Grade 1	Symptoms that are not life-threatening and only require symptomatic treatments (e.g., fever, nausea, fatigue, headache, myalgia, and discomfort).
Grade 2	Symptoms that require moderate intervention, and result in responses and require the inhalation of less than 40% oxygen or result in the responses to fluids or low-dose vasopressin, or grade 2 organ toxicity.
Grade 3	Symptoms that require active intervention, and result in responses and the inhalation of less than 40% oxygen, or hypotension which requires higher dose or multiple vasopressins, or grade 3 organ toxicity, or grade 4 elevated transaminase.
Grade 4	Symptoms that are not life-threatening and require ventilator support, or grade 4 organ toxicity (excluding elevated transaminase).
Grade 5	Death

The algorithms shown in Table 3 below use the CRS grading system detailed in Table 2 and are recommended to provide guidelines on CRS management related to targeted GUCY2C CAR-T therapy. The CRS management strategy is based on the experience acquired from the current use of anti-CD19 CAR+ T-cell products (Lee 2014).

Table 3 Treatment guidelines for cytokine release syndrome

Grading evaluations of cytokine release syndrome	Extensive comorbidity or older age?<br>No/Yes	Treatment
Grade 1：<br>Fever (defined as≥ 38.3℃)<br>Systematic symptoms	N/A	Supportive nursing requiring caution<br>Infection evaluation<br>Treatments of fever and neutropenia, if any, monitoring of fluid balance, and the uses of antipyretics and analgesics as required
Grade 2：<br>Hypotension: responses to fluids or a low dose of vasopressin<br>Hypoxia: response rate < 40% O2<br>Organ toxicity: grade 2	No	As described in grade 1 illness<br>Close monitoring of the functions of the heart and other organs
Grade 2：<br>Hypotension: responses to fluids or a low dose of vasopressin<br>Hypoxia: response rate < 40% O2<br>Organ toxicity: Grade 2	Yes	As described in grade 2 illness<br>Tocilizumab (8 mg/kg) ± corticosteroid (e.g., methylprednisolone 1 mg/kg, bid) or dexamethasone 10 mg, q6 hrs) shall be considered
Grade 3：<br>Hypotension: requiring multiple vasopressins or a high dose of vasopressin a<br>Hypoxia: response rate ≥40% O2<br>Organic toxicity: grade 3 or 4 elevated transaminase	N/A	

Grade 4<br>Mechanical ventilation<br>Organic toxicity: grade 4 toxicity, excluding elevated transaminase	N/A	As described in grade 2/3 illness<br>Corticosteroids (e.g., methylprednisolone 1 g/d x 3, followed by rapid decreased dosage, including 250 mg, bid, x 2 days, 125 mg, bid x 2 days, then 60 mg, bid x 2 days)

[a] Refer to Table 4

Table 4 Vasopressor

Definition of a high-dose vasopressin (all doses shall be administered ≥ 3 hours)

Vasopressin	Dosage
Norepinephrine monotherapy	≥20 μg/min
Dopamine monotherapy	≥10 μg/kg/min
Phenylephrine monotherapy	≥200 μg/min
Epinephrine monotherapy	≥10 μg/min
In case of a vasopressin	Vasopressin + norepinephrine equivalent to ≥10 μg/min
In case of no compound vasopressin (non vasopressin)	Norepinephrine equivalent to ≥20 μg/min*

[a] Equivalent equation of VASST vasopressin: equivalent dose of norepinephrine =[norepinephrine (μg/min)] + [dopamineμg/kg/min] ÷2] + [epinephrine (μg/min)] + [phenylephrine (μg/min)÷10]

6.4.1.1 Hypotension and renal insufficiency

Hypotension and renal insufficiency should be treated as shown in this section or according to medical judgment and medical guidelines. Progressive intravenous (IV) rehydration may be required to manage hypotension and vascular leakage caused by CRS. The subjects should be closely monitored to prevent excessive fluids, and continuous veno-venous hemodialysis may be required in some cases.

Invasive hemodynamic monitoring, if pulmonary catheters are used, may be conducive to the concurrent development of severe capillary leakage, aggressive use of IVF, and/or optimal fluid management of pulmonary edema. Antihypertensive drugs should be discontinued when blood pressure drops below baseline levels.

Baseline blood pressure is defined as the mean of all blood pressures acquired within 24 hours prior to transfusion with targeted CAR T cells. The first step for the treatment of hypotension is intravenous injection of normal saline.

Subjects with systolic, diastolic or mean arterial pressure ≤80% of the baseline level or lower than the lower limit of the normal range shall be injected with 1L1 L of normal saline.

If hypotension has no adequate response within 1 hour, a second injection of appropriate volume will be administered as the investigator deems appropriate. If hypotension still persists after two injections, monitoring in the intensive care unit (ICU) should be considered, and vasopressin support should be

administered.

Note that these guidelines may be modified based on the medication guidelines and clinical characteristics of individual subjects, such as lung conditions, cardiac functions, and other factors.

6.4.1.2 Cardio-toxicity

CRS-induced cardiac manifestations may include arrhythmia, decreased ejection fraction/heart failure, myocardial ischemia, and cardiac arrest. Tachycardia is a common symptom of CRS, and drugs to slow sinus tachycardia should be prohibited. Hypotension shall be managed according to Section 6.4.1.1.

Patients with persistent hypotension who do not respond to rehydration therapy should be evaluated by echocardiography for decreased ejection fraction/heart failure. These toxicities should be managed in a timely manner based on medical judgment. Based on the clinical indications, patients with grade 2 cardiotoxicity or above should be monitored in the intensive care unit. Tocilizumab and corticosteroids should be administered according to Section 6.4.1. ECG and echocardiography are recommended for follow-up to monitor the progression from toxicity to potential remission.

6.4.2 Hemophilic lymphohistiocytosis

Hemophilic lymphohistiocytosis (HLH) is a clinical syndrome that includes an extremely severe systemic inflammatory response, cytokine release syndrome (CRS) and multiple organ dysfunction (Jordan 2011, La Rosee 2015), and its symptoms include fever, hemocytopenia, liver dysfunction with hyperbilirubinemia, coagulopathy, tissue hemophilia, and significantly elevated ferritin, C-reactive protein, and soluble interleukin-2 receptor (sIL-2R) (Jordan 2011, La Rosee 2015, and Porter 2015). Abnormalities of the nervous system are observed in approximately one-third of such patients.

Infections, abnormal neoplasms, autoimmunity and immunotherapy may trigger severe HLH (Abe 2002 Ferreria 2006, Lackner 2008, and La Rosee 2015). CRS and HLH may have similar clinical syndromes, with some of the same clinical and pathophysiological characteristics. The production of cytokines in activated T cells may result in the overactivation of macrophages and HLH.

HLH should be considered in cases of elevated liver function parameters or evidence of hemocytopenia with or without other CRS for no reason. Monitoring of C-reactive protein, ferritin and soluble IL-2R may be conducive to the diagnosis and definition of the clinical process. Bone marrow biopsy should be adopted to evaluate the role of erythrophagocytes. In view of the same symptoms as CRS,

the patients should be managed according to *the CRS Treatment Guidelines* (Table 3). Suspected HLH cases should also be discussed with the medical monitor.

6.4.3 Neurotoxicity

Neurotoxicity (e.g., encephalopathy, drowsiness, and aphasia) has been observed in patients treated with CAR T-cell therapy. The evaluations of new neurotoxicity ≥ grade 2 shall include nervous system examination (including MMSE), MRI of the brain, EEG, and cerebrospinal fluid (CSF) test based on clinical indications. In cases of severe neurotoxicity, endotracheal intubation may be required to protect the airway.

The neurotoxicity shall be managed with tocilizumab and corticosteroids according to Table 5. Tocilizumab 8 mg/kg and high-dose corticosteroids (e.g., methylprednisolone 1 g/d x 3, followed by rapid decreased dosage, including 250 mg, bid, x 2 days, 125 mg, bid x 2 days, then 60 mg, bid x 2 days) should be considered for the treatment of life-threatening neurotoxicity.

In the case of grade 2 neurotoxicity, levetiracetam at the initial dose of 500 mg, bid, may be administered to prevent seizures. In some cases, multiple antiepileptic drugs may be required to control seizures. If possible, sedatives should be prohibited unless seizures are required to be managed.

Leukoencephalopathy has been observed on MRIs of patients with neurotoxicity. However, the subjects shall be managed based on their clinical symptoms. Follow-up MRI is recommended to monitor the progression from leukoencephalopathy to potential remission.

Delayed neurotoxicity has been observed in some patients (approximately 1 month after discharge from the hospital when targeted dCAR-T is administered). The patients and their family members/caregivers should be warned of the risk at discharge, and the patient should be advised to seek immediate medical treatments if new symptoms of neurotoxicity develop. In addition, patients should be advised to avoid driving or operating heavy machinery within 1 month after discharge from the hospital and/or complete neurotoxicity remission.

Table 5 Neurotoxicity management guidelines

Neurotoxicity grading evaluation (CTCAE 4.03)	Treatment	Evaluation
Grade 1:<br>Symptoms:<br>Drowsiness- mild drowsiness or sleepiness;<br>Confusion- mild disorientation;<br>Encephalopathy - mild restricted ADL;<br>Maldevelopment- not affecting communication;<br>Simple partial seizures;<br>No loss of consciousness.	Supportive nursing requiring cautions	Neurological examination<br>Additional examinations based on clinical indications
Grade 2:<br>Symptoms:<br>Severe drowsiness and restricted instrumental ADL<br>Confusion - moderate disorientation;<br>Restricted instrument ADL<br>Encephalopathy- restricted instrumental ADL<br>Maldevelopment- moderately affecting active	Supportive nursing requiring cautions<br>Prophylactic antiepileptic drugs (e.g., levetiracetam 500 mg bid) are considered. In case of comorbidity (e.g., CRS of grade 2 or higher grade), tocilizumab 8 mg/kg with an interval of 1 hour (no more than 800 mg) is considered	In addition to nervous system examination, MRI of the brain and cerebrospinal fluid test shall be included<br>ECG is considered

Grade 3:<br>Symptoms:<br>Drowsiness- dullness or stiffness<br>Confusion- severe disorientation; restricted self-care ADL<br>Encephalopathy- restricted ADL<br>Maldevelopment- severe expression impairment that affects the ability to read, write, or communicate clearly<br>Multiple seizures, fatigue and restricted self-care ADL although drug intervention is administered;<br>Disability- complete fecal/bladder incontinences	Tocilizumab 8 mg/k, iv (no more than 800 mg), is administered within 1 hour<br>); if the symptoms are unstable or do not improve, tocilizumab shall be administered every 4-6 hours. If the symptoms are still aggravated after the administration with tocilizumab, corticosteroids (e.g., dexamethasone 10 mg, iv, q6 h, and methylprednisolone 1 mg/kg, bid) shall be considered<br>Prophylactic antiepileptic drugs shall be considered	as a clinical indication
Grade 4:<br>The consequence is life-threatening and requires emergency measures and mechanical ventilation<br>Life-threatening symptoms; long-term recurrent seizures	Corticosteroids (e.g., methylprednisolone 1 g/d x 3, followed by rapid decreased dosage, including 250 mg, bid, x 2 days, 125 mg, bid x 2 days, then 60 mg, bid x 2 days) shall be considered; in case of no previous history of medication, tocilizumab shall be administered according to grade 3 neurotoxicity guideline.<br>In case of no previous history of medication, prophylactic antiepileptic therapy shall be considered	

Refer to Section 6.4.3 for medication recommendations and details.

6.4.4 Fever and neutropenia

The sources of infections should be evaluated according to the medication guidelines. Acetaminophen and comfort care measures should be adopted to treat a fever. NSAIDs and corticosteroids should be used with caution. Subjects with neutropenia and fever should be treated with broad-spectrum antibiotics. Most subjects with high fevers should be continuously treated with intravenous rehydration (normal saline) therapy, especially in subjects with less liquid intake or tachycardia. Filgrastim shall be administered according to the published guidelines (e.g., American Association of Infectious Diseases).

6.4.5 Preventions against infections

The subjects should be treated with prophylactic drugs according to the NCCN guidelines to prevent Pneumocystis jiroveci pneumonia, herpesvirus and fungal infection.

6.4.6 Blood support products for thrombocytopenia and anemia

All blood products shall be irradiated to remove white blood cells. With complete blood cell count as the standard, the subjects should be transfused with platelets and concentrated red blood cells as needed. Hemoglobin shall be maintained > 8.0 mg/dL and platelet count > 20,000/mm3. Sufficient platelet support (e.g., platelet count > 50,000/mm3) shall be provided prior to a lumbar puncture. Leukocyte filters shall be used for all blood and platelet transfusions to lower the risks of sensitivity to WBCs and CMV infection.

6.4.7 Tumor lysis syndrome

All subjects with extremely high malignant tumor burdens but without allergies to chemotherapy should receive preventive treatment (e.g., allopurinol) according to the medication guidelines prior to preconditioning chemotherapy. Preventive treatment should be discontinued when the risk of tumor lysis disappears.

6.4.8 Prevention against deep venous thrombosis (DVT)

Deep venous thrombosis (DVT) should be prevented in all patients with reduced activity during hospitalization according to medication guidelines. According to the benefit/risk, low molecular weight heparin (LMWH) is recommended as long as the patients have no contraindications (e.g., recent surgery, bleeding, or platelet count <50,000/μL). A noninvasive mechanical intermittent pneumatic compression device should be used in patients for whom anticoagulants are unable to be administered due to bleeding risk or other concerns for the prevention of DVT (Lyman 2015).

6.4.9 Instructions on the uses of ACTEMRA, dasatinib, rituximab and ruxolitinib

If IL6 > = 200 or IFNg > = 150 is observed on Days 1-5 after CAR-T infusion, ACTEMRA should be administered on the same day;

If IL6 > = 500 or IFNg > 260 is observed after CAR-T infusion, ACTEMRA + dasatinib should be administered on the same day.

If CAR T cells have amplified to over 8*10^9/L or skin symptoms are present, dasatinib should be administered on the same day.

It is recommended that dasatinib be administered at a dosage of 50 mg/day and ACTEMRA be administered at half the dosage as recommended in the Instruction for Use.

7 Study procedures

The investigator shall refer to the evaluation plan for an overview of the procedures needed. The visit plan should be calculated from Day 0 of targeted CAR T-cell transfusion.

A summary of the study evaluation/procedures is shown below. The description of each stage of the study is provided in Section 7.12.

7.1 Informed consent form

Before the subjects participate in the clinical study, the investigator will be responsible for obtaining written informed consent forms from them after explaining the study design, expected benefits and potential risks. All the subjects are required to sign the updated informed consent forms (ICF) approved by the IRB/IEC and indicate the date prior to any specific study procedure or activity.

The informed consent process and the subjects' consent or refusal to participate in

the study will be recorded in the subjects' medical records. If the subjects agree to participate in the study, the subjects and the personnel evaluating the informed consent forms shall sign the informed consent forms and indicate the date. The signed originals of ICFs shall be kept according to the policy of the institution and the requirements of IRB/IEC, and the signed photocopies of informed consent forms shall be handed over to the subjects.

All the subjects enrolled in the study shall provide their consent again after the IRB/IEC have approved any updates of the ICFs if they participate in the study.

7.2 Demographic data

Demographic data, including sex, date of birth, race and ethnicity group, will be collected to investigate the correlation between safety and treatment efficacy in the remaining subjects.

7.3 Medication history and medical history

The relevant medical history prior to the reporting of any adverse event will be collected. The relevant medical history is defined as concurrent medical conditions usually recorded in a patient's referral records. All the findings will be recorded in the CRFs.

In addition to the medical history, all the histories related to a subject's illness, treatments, and responses to treatments will be collected and traced back to the original diagnosis.

The copies of case record forms of the subjects referred from other clinics or institutions to the participating study center shall be obtained.

7.4 Physical examination, vital signs and physical conditions

A physical examination will be performed during the screening period and at the date stipulated in the evaluation plan. Compared with baseline, all the changes identified during the follow-up examinations will be reported as adverse events.

Vital signs, including blood pressure, heart rate, oxygen saturation and body temperature, will be monitored before and after the targeted CAR-T transfusion during treatment with the investigational product/hospitalization, and routine monitoring (every 4-6 hours) will then be conducted during the hospitalization period. If a subject has a fever (body temperature of 38.3 °C or above) at any time during the hospitalization period, their vital signs will be monitored based on the clinical indications.

The ECOG scale will be used to measure physical conditions and to quantify the overall health and ADLs of the subjects.

7.5 Nervous system evaluation

The Mini Mental State Examination (MMSE) (Standard Version 2.0) will be adopted to standardize the nervous system evaluation. The MMSE is a 5- to 10-minute questionnaire composed of 11 questions that examine various cognitive functions: orientation, attention, immediate recall, short-term recall, language, and the ability to follow simple language and written instructions.

The Mini Mental State Examination is divided into two parts. The first part requires the subjects to give verbal answers to the examiner's questions. The second part of the examination requires the subjects to follow oral and written instructions, to write a sentence naturally and to draw a geometric shape.

A complete neurological evaluation will be performed to determine the baseline level during the screening period. Follow-up evaluations will be performed on Day 0 and Day 1 prior to targeted CAR-T therapy and then every other day during hospitalization and during visits at week 4 and at month 3. The same investigator shall be familiar with or be trained on the use of the MMSE to minimize the difference between different examiners.

7.6 Heart function

The heart function of each subject will be measured based on left ventricular ejection fraction (LVEF) to confirm his or her eligibility to be enrolled in the study during the screening period. Evidence of no pericardial effusion will be confirmed according to the inclusion criteria. Echocardiography will also be performed to assess LVEF and pericardial effusion before a subject participates in the study. Echocardiography may be performed before the last chemotherapy and within 28 days before the signing of formed consent forms.

A 12-lead ECG will also be performed during the screening period to determine the baseline level.

7.7 Magnetic resonance imaging

Each subject will undergo a screening MRI of the brain to exclude metastasis to the CNS during the screening period of the study.

7.8 Bone marrow biopsy

For the patients who may have achieved complete remission following the targeted CAR-T product, follow-up bone marrow puncture and biopsy will be performed in those who have bone marrow involvement before the therapy or in those with new abnormalities in peripheral blood cell count or clinically suspicious posttreatment bone

marrow involvement combined with colorectal cancer caused by blood smear. Complete remission will be confirmed only if bone marrow puncture and biopsy indicate morphological evidence free of disease or, if not determined morphologically, the immunohistochemical examination findings are negative. For the evaluation requirements of treatment responses based on *the Response Evaluation Criteria in Solid Tumors (RECIST) (Version 1.1)*, refer to Section 7.9 and Appendix 1 (Cheson 2007).

7.9 Disease remission evaluation

The disease remission of the subjects will be evaluated by the investigator of the central laboratory according to the schedule stipulated in the evaluation plan. The disease will be evaluated according to *the Revised Response Evaluation Criteria in Solid Tumors RECIST (Version 1.1*). Flow cytometry and molecular or cytogenetic studies conducted for the experiment will be used as supplementary examinations but will not be solely used to determine the remission.

Baseline PET-CT scans of the neck, chest, abdomen, and pelvis are required, as well as appropriate imaging of all the other diseased sites. The subjects will undergo the first scheduled PET-CT tumor evaluation for the targeted CAR-T transfusion 4 weeks after the targeted CAR-T transfusion. In the post-treatment and long-term follow-up periods of the study, the evaluation will be performed on a regular basis according to the evaluation plan.

Disease evaluation following the targeted CAR-T therapy will be performed to determine the disease progression time. The subjects with symptoms indicating disease progression should be evaluated for disease progression based on the incidences of such symptoms, even if the evaluation does not fall on the scheduled time as stipulated in the evaluation plan.

Bone marrow puncture and biopsy will be performed in the subjects who are evaluated as complete remission. According to *the Response Evaluation Criteria in Solid Tumors RECIST (Version 1.1*) (Eisenhauer, 2008), follow-up bone marrow puncture and biopsy will be performed only in the subjects who have bone marrow involvement combined with lymphoma before the therapy or in those with new abnormalities in peripheral blood cell count or clinically suspicious posttreatment bone marrow involvement combined with lymphoma caused by blood smear. Complete remission will be confirmed only if bone marrow puncture and biopsy indicate morphological evidence free of disease or, if not determined morphologically, the immunohistochemical examination findings are negative.

If the subject is eligible for another targeted CAR-T therapy, the last scan prior to another therapy will be considered the baseline for the evaluation of posttreatment remission.

7.10 Laboratory

The following samples will be collected at the time points stipulated in the evaluation plan. Additional samples (e.g., blood, urine, cerebrospinal fluids, tissues, etc.) may be collected as required for further safety and efficacy tests.

Local laboratory analysis:

Sodium (NA), potassium (K), chloride (Cl), total CO2 (bicarbonate), creatinine, glucose, blood urea nitrogen (BUN), albumin, total calcium, total magnesium (Mg), inorganic phosphorus, alkaline phosphatase, ALT/GPT, AST/GOT, total bilirubin, direct bilirubin, LDH, and uric acid

C-reactive protein (CRP)

Complete blood cell count and differential counting

The urine or serum sample of a woman of childbearing age must be evaluated, and if the screening pregnancy test is positive, she should not be included in the study. If standard care pregnancy test findings are collected during the study and the results are positive, the investigator shall contact the medical monitor for guidance. If the female partner of a male subject is pregnant during the study, the subject should report this to the medical monitor to obtain further instructions.

Central laboratory analysis:

Blood will be collected for lymphocyte subset, cytokine and anti-CAR+ T-cell analysis at the interval as stipulated in the evaluation plan.

Serum samples will also be collected for the evaluation of anti-targeted CAR-T antibodies, which will be used for central laboratory analysis.

o If any serum sample shows an increase in anti-targeted CAR-T human bovine (HABA) antibody relative to the baseline level at the 3-month visit, additional serum samples will be collected and tested approximately every 3 months until the antibody restores to baseline level (or becomes negative) or 1 year has passed after the treatment, whichever is earlier.

Fresh tumor samples will be collected from the subjects whose tumor tissues have been stored and those who have signed the optional part of the informed consent forms for central reviews to evaluate the specific prognostic markers associated with advanced

colorectal cancer and the tumor immune environment. Additional analysis may include CAR-T-cell expression analysis, gene expression profiling analysis and DNA change analysis. The remaining tumor samples may be preserved for further exploratory analysis of DNA (somatic mutation), RNA or protein markers.

7.11 Biomarkers

The biomarkers in blood and tumor samples will be analyzed to evaluate the predictability of the targeted CAR-T therapy and pharmacodynamic markers. The specific prognostic markers associated with the tumor immune environment may also be evaluated during biopsies of preserved and fresh tumors.

7.12 Descriptions of study periods

The study center will retain the log recording of all the subjects who have passed the screening and have undergone the reviews and evaluations during the study. The information in the screening log shall include limited information, such as the date of screening, the date of enrollment or the reasons for failures of screening.

7.12.1 Screening

The screening period starts from the date when the subjects sign the ICFs approved by the IRB/IEC and ends when all the subjects have been confirmed to be enrolled. The informed consent form will be obtained prior to any study-specific procedure for nonstandard care. A procedure that is part of standard care but is not considered a study-specific procedure shall be performed prior to the signing of the informed consent form and should be used to confirm the inclusion criteria. These data shall be verified within the period shown below and stipulated in the evaluation plan.

The subjects will participate in the screening to confirm if they meet the inclusion criteria and will participate in the study after signing the written informed consent. Only the subjects who meet the inclusion criteria listed in Section 5 and have undergone leukapheresis will be enrolled. If a subject fails to meet the inclusion criteria at any time before enrollment, he or she will be deemed to fail the screening, the failure of the screening will be recorded in the subject screening log, and the reason for the failure will be indicated.

The following evaluations/procedures will be performed at the time points stipulated in the evaluation plan during the screening period:

Medical history and disease evaluation

Physical examinations including height and body weight

Subjects with symptoms of central nervous system malignancy, such as new onset of severe headache, neck stiffness, or any local nervous system findings during the physical examination, will undergo lumbar puncture and cerebrospinal fluid test.

Vital signs including blood pressure, heart rate, oxygen saturation and body temperature

ECOG for the evaluation of physical conditions

Neurological evaluation including the MMSE

ECG

LVEF and echocardiography for the evaluation of pericardial effusion

o Echocardiography may be performed within 28 days prior to the last chemotherapy and the signing of the informed consent form to confirm the eligibility for the study.

Imaging examinations

o MRI of the brain

o Baseline PET CT of the neck, chest, abdomen and pelvis,

PET-CT may be performed before and after the final treatment and prior to the signing of the informed consent form to confirm eligibility for the study.

If PET CT was performed more than 28 days prior to the conditioning chemotherapy or if the subject received any anticancer therapy between the screening period and the conditioning chemotherapy, the scan shall be repeated to determine a new baseline level. PET CT should be performed as close as possible to the time of enrollment.

Laboratory tests

o Chemical test

o Complete blood cell count and differential counting

o Virology tests (HIV, HBV, HCV, syphilis, EBV, and CMV)

o β- HCG pregnancy test for all women of childbearing age (serum or urine pregnancy test)

Reporting of serious adverse events (refer to Section 9).

Records of concomitant medication and previous history of cancer treatments

Preserved tumor samples as well as fresh tumor samples and cerebrospinal fluid samples will be collected from the subjects (applicable to the subjects signing the optional part of the informed consent form) once their eligibility for the study has been confirmed.

7.12.2 Rescreening

The subjects who fail to meet the inclusion criteria are not allowed to be rescreened. The subjects will undergo the evaluation that initially results in their failures of the screening, including any other procedures (i.e., laboratory evaluations or PET-CT scan) outside the designated screening window.

7.12.3 Enrollment/leukapheresis

Prior to leukapheresis, the subjects should be confirmed to be free of clinically significant infection. If a subject develops a clinically significant infection immediately before leukapheresis, cell collections should be delayed until the event is resolved. If the period of delay exceeds 5 days, the complete blood cell count and differential counting and chemical test at baseline should be repeated to confirm eligibility for the study. A subject who has undergone leukapheresis will be considered to be included in this study.

The following procedures/requirements will be met on the same day of leukapheresis according to the evaluation plan:

Vital signs including blood pressure, heart rate, oxygen saturation and body temperature

Body weight and height

Laboratory tests (blood will be collected before and on the same day of leukapheresis or the day before leukapheresis)

- o Chemical test
  - o Complete blood cell count and differential counting
  - o C-reactive protein (CRP); if CRP is ≥ 100 mg/dL, the medical monitor should be informed of before chemotherapy.
  - o Anti-CAR+ T cells
  - o Lymphocyte subset test
  - o Cytokines
  - o Anti-targeted CAR-T antibody

Leukapheresis period

Reporting of adverse events/serious adverse events

Recording of concomitant medication

7.12.4 Preconditioning chemotherapy period

If any screening evaluation or procedure is repeated between the screening period and the conditioning chemotherapy and the results do not meet the inclusion criteria (Section 5), please contact the medical monitor before the conditional chemotherapy.

Prior to conditioning chemotherapy, the subjects shall be free of clinically significant infection, free of clinically significant cardiac dysfunction, or free of acute neurotoxicity of > 1 grade (except peripheral sensory neuropathy), with serum creatinine < 2x the upper limit of the normal range. If an event not meeting these criteria happens immediately before the conditioning chemotherapy, the conditioning chemotherapy shall be postponed until the event is relieved. In addition, if the C-reactive protein (CRP) collected on the same day of leukapheresis is higher than 100 mg/dL or if the body temperature is higher than 38.0 °C within 48 hours prior to chemotherapy, please contact the medical monitor before continuing chemotherapy.

Any day from Day -5 to Day-3 from the time points as stipulated in the evaluation plan will be selected to perform the following procedures:

Vital signs including blood pressure, heart rate, oxygen saturation and body temperature

Laboratory tests (blood collection prior to chemotherapy)

- o Chemical test
- o Complete blood cell count and differential counting

Administrations with fludarabine and cyclophosphamide

Reporting of adverse events/serious adverse events

Recording of concomitant medication

7.12.5 Treatment period of the investigational product

The subjects will be hospitalized for targeted CAR-T therapy, and then observed for a cycle. Prior to the targeted CAR-T transfusion, the subjects should be free of clinically significant infection or clinically significant cardiac dysfunction, with serum creatinine < 2 x upper limit of the normal range, and they should be free of > grade 1 acute neurotoxicity (except peripheral sensory neuropathy). Moreover, the subjects should not be administered systemic antimicrobial drugs for the treatment of active infection (prophylactic use of antimicrobial drugs is allowed) within 48 hours before the

administration of the targeted GUCY2C CAR-T product. If the subjects fail to meet these criteria prior to targeted CAR-T therapy, the targeted CAR-T transfusion should be delayed until the event is resolved. If the target CAR-T transfusion was delayed for more than 2 days, the conditioning chemotherapy was repeated. In all cases of delayed transfusions with targeted CAR T cells, the medical monitor should be informed of and provide guidance.

In addition to the above criteria, if a subject's body temperature is ≥ 38.0°C within 48 hours prior to the targeted CAR-T transfusion, the medical monitor should be informed of before the targeted CAR-T transfusion is continued.

The subjects will be hospitalized for 7 days after the targeted CAR-T therapy ends. The subjects should not be discharged from the hospital until the target CAR T-cell-related nonhematological toxicity is restored to ≤ grade 1 or the baseline level. If the investigator believes appropriate, the subject may be discharged from the hospital even if he or she develops noncritical and clinically stable toxicity or the toxicity > grade 1 has been improved gradually (e.g., renal insufficiency). If the subject develops persistent targeted CAR-T therapy-related fever, hypotension, hypoxia or persistent central neurotoxicity of > grade 1 or if the treatment investigator believes appropriate, he or she may continue to be hospitalized.

In view of the possibility of CRS or neurotoxicity after their discharge from the hospital, a subject should be diagnosed for any potential symptoms, such as fever, dyspnea, unconsciousness, aphasia, dysplasia, drowsiness, encephalopathy, ataxia or tremor, and their family members/caregivers should be informed of this. If the subject develops these symptoms, they should be informed to promptly contact the principal investigator or to immediately seek medical advice.

During this period, the following procedures should be completed at the time points stipulated in the evaluation plan:

Mini Mental State Examination (MMSE)

o The MMSE will be performed prior to the targeted CAR-T therapy on Day 0, then on Day 1 and every other day during hospitalization.

Vital signs, including blood pressure, heart rate, oxygen saturation and body temperature, will be checked every 4-6 hours during hospitalization.

Laboratory tests (prior to the targeted CAR-T transfusion, as described in the evaluation plan)

- o Chemical test

- Complete blood cell count and differential counting, lymphocyte subset test, and cytokine test
- Anti-CAR+ T cells

Targeted CAR-T transfusion

If applicable, the subjects with ≥ 2 new neurological symptoms after the targeted CAR-T transfusion or those who have signed the optional part of the informed consent forms shall undergo lumbar puncture to test cerebrospinal fluid.

Cancer samples will be collected from the subjects who have signed the optional part of the informed consent forms (at any time between Day 7 and Day 14)

Reporting of adverse events/serious adverse events (refer to Section 9)

Recording of concomitant medication

The monitoring of C-reactive protein, ferritin, and LDH levels (only when LDH levels are elevated at baseline) may be conducive to diagnosing and defining CRS/neurotoxicity-related clinical processes. Therefore, daily monitoring of C-reactive protein, ferritin and LDH levels (if elevated at baseline) from Day 0 to the end of hospitalization is recommended. In addition, lactate levels should be monitored based on the clinical indications.

7.12.6 Post- treatment evaluation period

All the subjects will be followed up in the post-treatment evaluation period after the targeted CAR-T-cell transfusion and discharge from the hospital. The subjects will return to the clinic at the following intervals from Day 0 (the day on which the targeted CAR-T transfusion is performed).

Week 2 (± 2 days)

Week 4 (± 3 days)

Month 2 (± 1 week)

Month 3 (± 1 week)

The subjects will allow the contact person of the primary sponsor to continue to access the medical records to obtain information about their health status and initial treatment responses. The subjects will undergo the following procedures according to the evaluation plan:

MMSE

PET-CT scan for disease evaluation: if the resolution of the PET-CT scan is not

high enough, the scan should be repeated.

Physical examination

Vital signs including blood pressure, heart rate, oxygen saturation and body temperature

Laboratory tests

- o Biochemical test
- o Complete blood cell count and differential counting
- o β- HCG pregnancy test for all women of childbearing age (serum or urine pregnancy test)
- o Anti-targeted GUCY2C CAR-T antibody
- o Cytokine levels
- o Lymphocyte subset test
- o Anti-CAR+ T cells

Reporting of adverse events/serious adverse events (refer to Section 9)

Recording of concomitant medication

If the subjects are subsequently rehospitalized because of any targeted CAR-T therapy-related adverse events, they will undergo the following procedures according to the evaluation plan:

Laboratory tests

o Anti-CAR + T cells will be performed upon admission, once a week, and upon discharge.

o Cytokine levels will be tested upon admission, then once a week, and upon discharge.

If the subjects do not respond to the treatment at any time during the post-treatment evaluation period (i.e., they have achieved CR or PR) or their illnesses do not progress after the responses, the subjects will be directly included in the 3-month visit, and their survival time and the long-term follow-up outcomes of their illnesses will be followed up.

7.12.7 Long-term follow-up period

All the subjects enrolled in the study will be followed up for survival time and illness status (if applicable) during the long-term follow-up period. The subjects will be included in the long-term follow-up period (when the patients respond to the treatment or

are directly included in the 3-month visit due to disease progression) at the 3-month visit during the posttreatment evaluation period.

Every 3 months (± 2 weeks) until month 18

Every 6 months (± 1 month) from month 24 to month 60

The subjects enrolled in the study who have received the targeted CAR-T therapy will undergo the following procedures at the time points stipulated in the evaluation plan:

Physical examination

PET-CT/disease evaluation until month 24 or until disease progression, whichever is earlier. Twenty-four months later, the results of routine disease evaluations of the subjects whose illnesses have not progressed in the first 24 months will be collected according to standard care.

Survival status

Laboratory tests

- o Complete blood cell count and differential counting
- o Anti-targeted CAR-T antibody (refer to Section 7.10)
- o Lymphocyte subset test
- o Anti CAR + T cells

Follow-up treatment of advanced colorectal cancer

Reporting of the targeted adverse events/serious adverse events (until month 24 or until disease progression, whichever is earlier), including nervous diseases, blood diseases, infections, autoimmune diseases and secondary tumors, until disease progression.

Records of targeted concomitant medication (2 years after disease progression), including gamma globulin, immunosuppressive drugs, anti-infective drugs and vaccination

The subjects may also be contacted via phone to confirm their survival status, and they may report the uses of targeted concomitant drugs. If laboratory samples are required to be collected from the subjects, the laboratory samples may be collected in the clinic or an external institution to reduce the inconvenience caused to the subjects.

The patients enrolled in the study who have received the targeted CAR-T therapy will undergo the following procedures/evaluations at the time points stipulated in the evaluation plan:

Follow-up treatment of advanced colorectal cancer

Survival status

Disease evaluation according to standard treatment

Reporting of adverse events/serious adverse events until 30 days after the last procedure (e.g., leukapheresis and conditioning chemotherapy).

If a subject fails to return to the clinic for any scheduled protocol-specific visit, the center will try to contact him or her via phone and e-mails twice. The center shall record the two attempts to contact the subjects. If the subject does not respond within 1 month after the second contact, the subject shall be considered lost to follow-up, and no additional contact will be needed.

7.12.8 Retreatment

Subjects who have achieved PR or CR at the first time may choose to receive the second course of pretreatment chemotherapy and targeted CAR-T therapy, if their diseases progressed 3 months after administration with targeted CAR-T cells and malignant tumor cells recur as determined by the treatment investigator, the medical monitor should be consulted and the subject should give their consent. Prior to any study-related procedures or treatments, the benefits and risks of another treatment shall be discussed with the subjects, including the possibility of a second time of leukapheresis, which shall also be recorded in a subject's original archive.

The subjects eligible for the second course of treatment shall be re-evaluated, and they are required to continue to meet the original inclusion criteria of the study (except for the previous use of targeted CAR-T cells in this study) and shall not receive the follow-up treatment for colorectal cancer. Moreover, any fludarabine- or cyclophosphamide-related toxicity shall be lowered to ≤1 grade 1 or restored to baseline level prior to the retreatment, except for hair loss. Each subject shall undergo retreatment for as long as 1 course. The subjects who receive retreatment should follow the same treatment plan and meet the procedure requirements according to the initial treatment.

The subjects enrolled in the study will follow the same targeted CAR-T regimen at the original target dose.

Subjects with ≥ grade 3 toxicity in the study will not be eligible for retreatment. Moreover, if a subject presents known neutralizing antibodies, the subject will not be eligible for retreatment.

However, in the case of nonneutralizing HABA antibodies, the subject may receive retreatment if the inclusion eligibility criteria are met.

Evaluation plan (1-day preconditioning therapy)

Procedures	Screeni	Enrollment/leukapheresis period	Preconditionin					Treatment		Follow up visit after the treatment (each visit will be calculated from Day 0)			
	Within 28 days after the enrollment	Within 5 days after confirmation of the inclusion criteria	-5	-4	-3	-2	-1	0	1-7	Week 2 (±2 day)	Week 4 (±3 days)	Month 2 (±1 week)	Month 3 (±1 week)
Medical history & disease evaluation	X												
ECOG score for the evaluation of physical	X												
Mini Mental State Examination (MMSE)	X							X	QOD [4]		X		X
Electrocardiogram	X												
Echocardiography	X												
Preserved/fresh tumor stored at the central		X							Between Day 7 and Day 14				
MRI of the brain	X												
PET-CT/disease evaluation[2]	X										X		X
Physical examination	X										X	X	X
Vital signs (BP, HR, O2 saturation, body temperature)	X	X	X	X	X			X	X	X	X	X	X
Height and body weight	X	X											
Pregnancy test (serum or urine)	X												
Blood collection for CD 19 test	X												
Blood collection for biochemical test	X	X	X	X	X			X	X	X	X	X	X
Blood collection for complete blood cell count and differential counting	X	X	X	X	X			X	X	X	X	X	X
Blood collection for virology tests (HIV, HBV, HCV, syphilis, EBV, and CMV)	X												
Blood collection for CD 19 test	X												
Blood collection for C-reactive protein (CRP)		X											
Blood collection for anti-targeted GUCY2C CAR-T antibody test[3]		X									X		X
Blood collection for lymphocyte subsets test		X						X			X		X
Blood collection for cytokine test[4]		X						X	QOD [4]		X		
Blood collection for CAR +T-cell test[6]		X						X	Day 7		X		X
Leukapheresis period		X											
Fludarabine/cyclophosphamide			X	X	X								
Intravenous infusion of targeted CAR-T cells								X					
Adverse events/combined use of drugs	X												

Evaluation plan (notes to the 1-day conditioning chemotherapy)

[1] Preserved tumor samples: FFPE masses or 20 unstained sections of fresh tumor samples collected from the subjects who signed optional informed consent forms, refer to Section 7.11. Preserved and fresh tumor samples (if applicable) will be submitted to the central laboratory after the eligibility of the subjects has been confirmed and prior to conditioning chemotherapy. Fresh post-treatment tumor samples will be collected/submitted (if applicable) at any time during the period between Day 7 and Day 14.

[2] PET-CT scans (neck, chest, abdomen and pelvis): the baseline scan shall be repeated if the PET-CT scan was performed > 28 days before the conditioning chemotherapy or if the subject received any anticancer treatment between the screening period and the conditioning chemotherapy. PET-CT scans for screening should be performed as close as possible to enrollment.

[3] Blood collection for the anti-targeted CAR-T antibody test: baseline antibody samples will be collected prior to leukapheresis. Antibody samples treated with targeted CAR T cells will be collected at week 4 and at month 3. Refer to Section 7.10 for more details.

[4] MMSE and cytokines: performed prior to the targeted CAR-T transfusion on Day 0 and then on Day 1 and every other day during hospitalization.

[5] Lumbar puncture: Subjects with symptoms of CNS malignancy (e.g., new onset of severe headache, neck stiffness, or local nervous system findings) will undergo lumbar puncture at screening to evaluate cerebrospinal fluid for any possible CNS involvement. After the targeted CAR-T transfusion, the subjects with new neurological symptoms ≥ grade 2 will undergo lumbar puncture to evaluate cerebrospinal fluid. In addition, the subjects who signed the optional part of the informed consent forms will undergo lumbar puncture to collect cerebrospinal fluid before and after the targeted CAR-T transfusion (Day 5 ± 3).

[6] If the subjects are subsequently rehospitalized and develop any targeted CAR-T therapy-related adverse events, blood samples will be collected for the collection of anti-CAR+ T cells and cytokines upon admission and then once a week and upon discharge.

Evaluation schedule (long-term follow-up period)

Procedures	Long term follow-up period (each visit calculated from Day 0)											
Visit frequency	Month 6	Month9	Month12	Month15	Month18	Month24	Month30	Month36	Month42	Month48	Month54	Month60
Physical examination[1]	X	X	X	X	X	X						
PET-CT (neck- chest- abdomen- pelvis)[2]	X	X*	X*	X*	X*	X*						
Illness evaluation	X	X	X	X	X	X	X	X	X	X	X	X
Survival Status	X	X	X	X	X	X	X	X	X	X	X	X
Blood collection for whole blood cell count/differential counting[3]	X	X	X	X	X	X						
Blood collection anti-targeted CAR-T antibody test[4]												
Blood collection for lymphocyte subsets test[3]	X	X	X	X	X	X						
Blood collection for anti CAR + T-cell test[3]	X		X			X						
Specific AE/SAE[5]	X	X	X	X	X	X						
Targeted concomitant medication[6]	X	X	X	X	X	X	X	X	X	X	X	X
Follow up treatment of advanced colorectal cancer[7]	X	X	X	X	X	X	X	X	X	X	X	X

1 Physical examination will continue until month 24

2 PET-CT scan will continue until month 2 or until disease progression, whichever is earlier

3 Subjects will continue to provide samples for complete blood count, white blood cell count/differential counting, lymphocyte subsets test and anti-CAR + T-cell test until month 24

4 Anti-targeted CAR-T antibody samples, refer to Section 7.10

5 Targeted adverse events/serious adverse events will be collected and reported until month 24 or until disease progression (whichever is earlier)

6 Data on targeted concomitant medication will be collected until 2 years after disease progression

7 The data on the follow-up treatments following the targeted CAR-T transfusion for the treatment of the subject's disease will be collected, such as nonstudy-specific chemotherapy, immunotherapy,

8 Withdrawal of the subjects

The subjects have the right to withdraw from the study at any time for any reason, and their visits to the doctor or institution for drug therapy in the future will not be affected.

The subjects may refuse to continue the therapy required by the study and/or other procedures required by the protocol at any time during the study period, but they may continue to participate in the study, which is referred to as a partial withdrawal from the study.

In case of any partial withdrawal of the informed consent form, the investigator shall discuss with the subjects the appropriate processes for discontinuing the investigational product, trial therapy, or other procedures required by the protocol and the options for continuing the study, completing the process, and collecting the relevant data as stipulated in the evaluation plan. The investigator shall also discuss the follow-up visits and communication methods with the subjects, which shall be recorded in the original files.

A complete withdrawal of the informed consent form to participate in the study means that the subjects are not willing to receive further treatments or procedures required by the protocol and are not willing to continue further follow-up visits of the study. The subjects' data collected before the withdrawal of the informed consent form will be retained and included in the study analysis, and the withdrawal of the informed consent form will be included in the publicly available data (death records), if possible. The investigator shall also discuss the appropriate procedures for withdrawals from the study with the subjects.

Some study centers may be required to retrieve publicly available records to determine survival status, so the data of any subject whose survival status is unknown will be collected, if available. Autopsy reports issued during the study may also be required to confirm the illness conditions at the time of death.

The investigator and/or the sponsor may also decide to withdraw any subject from the treatment with the investigational product and/or other procedures stipulated in the protocol or the whole study at any time before the completion of the study.

8.1 Reasons for discontinuation of the treatment

The reasons for being asked to withdraw from the treatments with the investigational product or procedures required by the protocol are as follows:

Adverse events

Required by the subjects/noncompliances of the subjects

Nonapplicability of the product

Lost to follow-up

Death

Decided by the sponsor

8.2 Reasons for exclusion from the study

The reasons for excluding the subjects from the study are as follows:

Withdrawals of informed consent forms for further follow-up visits by the subjects

Decided by the investigator

Lost to follow-up

Death

9 Safety report

9.1 Adverse events

An adverse event is defined as a medical adverse event that happens when the subjects enrolled in the clinical trial have been administered the investigational product at any dose. The event is not necessarily related to the trial therapy. The investigator has the responsibility of guaranteeing that any adverse events observed by him or her or reported by a subject are recorded in the subject's medical records.

Adverse events included aggravation of the existing medical conditions. Aggravation refers to increased severity, shortened frequency, or prolonged duration of the existing medical conditions or is associated with worsened outcomes. No aggravation or intervention may occur during the study period; for example, an elective plastic surgery or a medical procedure during the study period will not be considered an adverse event.

An intervention prior to the treatment (e.g., an elective cosmetic surgery) or a scheduled medical procedure prior to participation in the study will not be considered an adverse event. Hospitalization for trial therapy or for preventive measures according to the policy of the institution will not be considered an adverse event.

The term "disease progression" determined and evaluated by imaging or other methods for cancer lesions shall not be reported as an adverse event. Death induced by disease progression without signs and symptoms shall be reported as an event of primary tumor (e.g., colorectal cancer).

In case of any symptoms and signs of the adverse events induced by the illness stipulated in the study, the deterioration of cancer symptoms and signs that happen during the study shall also be reported as an adverse event and recorded in the corresponding part of the case report form.

Whether the treatment will be discontinued due to any adverse event will be determined according to the clinical judgment of the investigator. If a subject requests to withdraw from the treatment as required by the protocol or from the study due to an adverse event, the subject shall undergo the procedures of the 3-month visit as stipulated in the evaluation plan.

If a subject starts a new anticancer therapy, the reporting of nonserious adverse events will be terminated upon the new anticancer therapy.

9.2 Reporting of adverse events

The investigator has the responsibility of guaranteeing that all the adverse events observed by him or her or reported by the subject from enrollment (i.e., start of leukocytosis) to 3 months after the targeted CAR-T transfusion are monitored and reported. The investigator will be required to monitor and report specific adverse events 3 months later, including nervous system diseases, blood diseases, infections, autoimmune diseases, and secondary malignant tumors, until month 24 or disease progression, whichever is earlier.

The reporting period of the subjects who have been enrolled but have not received the targeted CAR-T therapy will end 30 days after the last procedure (e.g., leukocytosis and conditioning chemotherapy).

The investigator shall determine the following information about the adverse event:

Adverse events or syndromes (signs or symptoms, if unknown)

Date of onset and remission

Severity

Correlation with the investigational product, conditioning chemotherapy or study procedures

Measures taken

The NCI Common Terminology Criteria for Adverse Events (CTCAE) (Version 4.03) will be used as the adverse event grading scale. Cytokine release syndrome will be reported using the grading scale stipulated in Table 2 of Section 6.4.1.

During the reviews of the adverse events, the investigator shall assess whether the adverse events may be related to the following circumstances:

1) The investigational product (targeted CAR-T therapy); 2) conditioning chemotherapy; or 3) any study procedures required by the protocol. The relationship will be indicated by the answer “yes” or “no” and recorded in the CRFs. The answer "yes" indicates that a causal relationship has been evidenced between the trial therapy or the procedure with the adverse event. Other relevant data on the description of adverse events will be collected and recorded into the CRFs.

The investigator is responsible for reviewing the laboratory test results to determine whether the outliers of an individual study subject represent clinically significant changes relative to the baseline. Abnormal laboratory findings of no clinical significance (based on the investigator's evaluation) will not be recorded as adverse events. Where applicable, clinical sequelae (not laboratory abnormalities) will be

recorded as adverse events. The investigator shall follow up the adverse events reported until they become stable or are relieved.

9.3 Definition of serious adverse events

The investigator will be responsible for reporting all the serious adverse events observed by him or her or reported by the subjects from enrollment to 3 months after the targeted CAR-T transfusion. The investigator will only report the serious adverse events observed by him or her or reported by the subjects 3 months later until month 24 or until disease progression, whichever is earlier. The reporting period of the subjects who have been enrolled but have not received the targeted CAR-T therapy will end 30 days after the last procedure (e.g., screening procedure and conditioning chemotherapy).

A serious adverse event is defined as an adverse event that meets at least one of the following criteria:

Death

Life-threatening events (resulting in direct risks of death)

Events requiring hospitalization or prolonged length of stay

Events resulting in permanent or severe physical disability/disability

Congenital malformations/birth defects

Other medically significantly serious events (if the event requires hospitalization at a medical institution, the adverse event will be deemed to meet the standard of "the events requiring hospitalization" (e.g., staying overnight)).

If the subject has been hospitalized but needs additional care, the event shall be recorded as a serious adverse event. The transfers from a routine ward to the ICU of the hospital or the incident resulting in prolonged length of stay will also be recorded as serious adverse events.

If the investigator believes that the event is clinically significant but does not meet the severity criteria, the event shall be classified as a serious adverse event according to the criteria of "other clinically significant serious adverse events".

9.4 Reporting of severe adverse events and ≥ grade 3 non severe CRS events and above

All serious adverse events and nonserious CRS events of ≥ grade 3 (Lee 2014; Table 2) shall be submitted to the safety department within 24 hours after the investigator has been informed of such events.

The progression of malignancy during the study period shall not be reported as a serious adverse event. Disease progression-related adverse events may be reported as serious adverse events. If the malignant tumor results in death within 3 months after the last conditioning chemotherapy or the targeted CAR-T therapy, the events resulting in death shall be recorded as CTC5 serious adverse events.

If the death happens during the reporting period of a serious adverse event, the death shall be reported

regardless of any intervention.

Any death within 3 months after the targeted CAR-T transfusion following the first conditioning chemotherapy, whether due to the therapy or not, will be reported within 24 hours. Any death occurring 3 months after the targeted CAR-T transfusion shall be reported within 24 hours only if it is considered to be related to such therapy.

9.5 Pregnancy and lactation

There is no relevant clinical experience on targeted CAR-T therapy in pregnant or lactating women, or no animal reproductive study has been conducted. Due to the potentially dangerous impacts of preconditioning chemotherapy on the fetus, women of childbearing age should test negative for pregnancy prior to enrollment. This experimental treatment should not be applicable to pregnant or lactating women.

If a female subject is pregnant or the female partner of a male subject enrolled in the study is pregnant within 6 months after the targeted CAR-T transfusion, the pregnancy report shall be submitted to the contact person of the primary sponsor. The primary sponsor may request information on the pregnancy and/or outcome.

In addition to the reporting of the pregnancy event during the study period, the investigator shall monitor female subjects and the pregnant female partner of the male subjects from the last dose of the targeted CAR-T therapy to month 6.

The pregnancy event shall be reported to the contact person of the primary sponsor within 24 hours after the investigator has been aware of the event. If a female subject breastfeeds during the treatment required by the protocol, the breastfeeding event will be reported to the contact person of the primary sponsor. In addition to the reported lactation event during the study period, the investigator shall monitor the lactation event after the last medication required by the protocol until month 6.

All breastfeeding events shall be reported to the contact person of the primary sponsor within 24 hours of the incidences of such events.

9.6 Criteria for the discontinuation of enrollment

As the review of the study, the sponsor will organize a meeting with the PI to evaluate the follow-up enrollment after 3 or 6 subjects have been followed up with the CAR-T therapy for 30 days. Enrollment will be suspended if the following conditions are met:

1) More than 10% of grade 5 CAR-T-related adverse events have occurred within 30 days; or

2) The incidence rate of grade 4 CAR-T-related adverse events lasting for 7 days is higher than 33%

Neurotoxicity

CRS (Lee 2014; standard)

Other serious nonhematological adverse events

Infection (treatment-related)

3) Preliminary results indicate that the therapy may be better or worse than the predesigned clinical hypothesis (agreed upon by the majority of attendees of the meeting).

10 Precautions for statistical analysis

10.1 General principles

Continuous variables will be expressed by the number of observations, mean, median, standard deviation, minimum and maximum, and categorical variables will be expressed by the frequency and percentage of each category. RECIST 1.1 will be adopted to evaluate the efficacy after CAR-T therapy. Safety evaluation will mainly consider the incidence rates of adverse events and serious adverse events, dose limiting toxicity (DLT) and cytokine release syndrome (CRS).

10.2 Analysis dataset

Modified intention to treat (merit): The modified intention to treat set includes all the subjects who have been enrolled in the study and have received the CAR-T targeted therapy at the target dose. The mITT set is the main set for efficacy analysis.

Safety set (SS): The safety set includes all the subjects who received the CAR-T targeted therapy and received at least one safety evaluation. The SS set is the main analysis set of safety evaluation.

Full analysis set (FAS): The full analysis set includes all the subjects enrolled in the study, which will be used to describe the characteristics of the subjects and to summarize the completion of the study and the conditioning chemotherapy. If necessary, the FAS set will also be used for sensitivity analysis of efficacy evaluation.

10.3 Study endpoint

10.3.1 Primary endpoints

The primary endpoint of this study is safety and the objective response rate (CR + PR) of the targeted CAR-T therapy for colorectal cancer. The efficacy evaluation will refer to RECIST 1.1.

10.3.2 Secondary endpoints

- Duration of overall response: the duration of sustained remission in subjects with objective responses is defined as the period from the first objective response (after the confirmation) to disease progression or recurrence. As of the end of data analysis, the data of the subjects who do

not meet the criteria of disease progression or death will be deleted on the last evaluable date of disease evaluation, and their remission status will be marked as sustained remission;

- Progression-free survival (PFS): Progression-free survival (PFS) is defined as the period from targeted CAR-T-cell transfusion to disease progression or death from any cause. As of the end of data analysis, the data of the subjects who do not meet the criteria of disease progression or death will be deleted on the last evaluable date of disease evaluation;
- Overall survival: Overall survival is defined as the period from CAR T-cell transfusion to death. As of the end of data analysis, the data of the subjects who are alive will be deleted on the last contact date;

Incidence rates of adverse events and serious adverse events;

Vital signs, physical examination, and laboratory tests;

Anti-targeted GUCY2C + CAR T antibody and anti- GUCY2C + CAR T cells in blood will be summarized;

Serum cytokines (IL-2, IL-6, TNF-α, IFN-γ, etc.) and the incidence rate of CRS;

VAS score < 4 points.

10.3.3 Exploratory endpoints

The developments of potential biomarkers will be investigated based on the evaluations of the proposed effects of blood cells, tumor cells and investigational products.

10.4 Statistical analysis method

10.4.1 Objective response rate

Point estimates and accurate two-sided 95% confidence intervals of the objective response rate (ORR) will be calculated.

10.4.2 Duration of sustained remission

The Kaplan–Meier method will be adopted to estimate the cumulative response rate and its two-sided 95% confidence interval.

10.4.3 Progression-free survival

The Kaplan–Meier method will be adopted to estimate the median progression-free survival and its two-sided 95% confidence interval. The proportion of surviving subjects and the estimated progression-free survival rates at months 3, 6 and 12 will be provided.

10.4.4 Overall survival

The Kaplan–Meier method will be applied to estimate the median overall survival and its two-sided 95% confidence interval. The estimated proportion of surviving subjects at months 3, 6 and 12 will be provided.

10.4.5 Safety

Safety will be evaluated by a summary of adverse events, changes in laboratory test findings and changes in vital signs. The MedDRA will be used to code adverse events. The number and frequency of adverse events will be summarized using the corresponding terms according to the system organ classification (SOC). Cytokine release syndrome (CRS) will be analyzed according to its severity and systemic organ classification. All adverse events will be graded according to the NCI CTCAE (version 4.03). All the SAEs (including death) and DLT will be tabulated and summarized separately.

11 Regulatory obligations

11.1 Confidentiality for the subjects

All the materials of the subjects submitted to the contact person of the primary sponsor shall be kept confidential. The following principles will apply:

The subjects will be marked with a unique identification number

The date of birth will be reported according to local laws and regulations.

Age upon enrollment

In case of the reporting of serious adverse events, the subjects will be identified by their subject identification numbers, initials and date of birth (in accordance with the local reporting requirements for initials and date of birth).

According to the national regulations of China and the ICH/GCP guidelines, the investigator and the institution are required to allow the sponsor, CRO, IRB/IEC, and regulatory authority to access the

original records of the subjects to verify the study data.

11.2 Responsibilities of subjects to affix their signatures

Each clinical study report will be signed by the coordinating investigator.

12 Amendments and termination of the protocol

The protocol, if revised, shall be subject to the approvals of the investigator and the IRB/IEC. The approval records confirmed by both parties will be submitted to the contact person of the primary sponsor.

The sponsor reserves the right to terminate the study at any time. Both the sponsor and the investigator reserve the right to suspend the investigator’s participation in the study according to the terms of the study contract. The investigator will provide the IRB/IEC with written correspondence on the completion or early termination of the trial and provide CRO with a copy of the correspondence.

The sponsor reserves the right of unilateral licensing at its discretion, including whether to prepare the targeted CAR T cells and to provide them to the study center and subjects after the completion of the study and prior to production and commercial marketing.

13 Study documents and retentions

The investigator will maintain a list of qualified personnel entrusted with the responsibilities for the study. The individuals authorized to perform such responsibilities shall be recorded and included in the Letter of Entrustment.

The original files refer to the original documents, data and records on the study data collected and verified, including but not limited to hospital records and patient records, laboratory reports, pharmacy reports, radiology records, subject dates, microfilms, correspondence information, and death registration.

The investigator and researchers are responsible for establishing a comprehensive and centralized archiving system for retaining the records of all subjects, which shall be easy to retrieve and be monitored and/or audited by the contact person of the primary sponsor, regulatory authority and the IRB/IEC. The archiving system will include at least the following information:

The subjects’ consent forms, including ICFs and the list of subject identification numbers

The protocol and amendments thereof, and copies of the investigator's brochure and prestudy records, as well as all the correspondences between the IRB/IEC and the sponsor.

Certificate of receipt, trial therapy process record, and correspondences related to the investigational product.

The supporting information of the original files recorded in the CRFs shall be kept in the study center and be provided on request. No study documents shall be discarded before written agreements have been concluded between the sponsor and the investigator. If the stored information is no longer available or the

original files archived have to be transferred to other locations, the investigator shall notify the contact person of the primary sponsor prior to transfers of such files.

14 Study monitoring and data collection

The contact person of the primary sponsor, monitor, auditor or supervisor is responsible for contacting and visiting the investigator, inspecting the institution, verifying the original files and records, and guaranteeing the confidentiality of the subjects.

During the study period, the monitor is responsible for verifying the CRF data of the original files on a regular basis and confirming the compliance and accuracy of the protocol and the consistency between the implementation of the study with data collection according to the regulations. The monitor will obtain the subject records as described in Section 13.

By signing the investigator agreement, the investigator agrees to cooperate with the monitor to determine and to solve the problems identified during the inspection visit. All the data will be collected and recorded in the electronic CRF system.

15 Publication

Any publication in any form regarding this study shall be submitted to the sponsor for review and approval. Any study contracts concluded between the institution, principle investigator and sponsor or their representatives will list the requirements for publication review.

References

Ardeshna K, Kakouros N, Qian W, et al. Conventional second-line salvage chemotherapy regimens are not warranted in patients with malignant lymphomas who have progressive disease after first-line salvage therapy regimens. *Br J Haematol* 2005 Aug;130(3):310-72.

Barrett DM, Teachey DT, Grupp SA. Toxicity management for patients receiving novel T-cell engaging therapies. *Curr Opin Pediatr*. 2014;26(1):43-49.

Bettelli E, Carrier Y, Gao W, et al. Reciprocal developmental pathways for the generation of pathogenic effector TH17 and regulatory T cells. *Nature*. 2006;441(7090):235-238.

Better M, Pugach O, Lu L, et al. Rapid cell expansion (RACE) technology for production of engineered autologous T-cell therapy: Path toward manageable multicenter clinical trials in aggressive NHL with anti-CD19 CAR. *Journal of Clinical Oncology.* 2014;32(15_suppl):3079-3079.

doi:10.1200/jco.2014.32.15_suppl.3079.

Biswas SK, Mantovani A. Orchestration of metabolism by macrophages. *Cell Metab.* 2012;15(4):432-437.

Blanc V, Bousseau A, Caron A, Carrez C, Lutz RJ, Lambert JM. SAR3419: an anti-CD19-Maytansinoid Immunoconjugate for the treatment of B-cell malignancies. *Clin Cancer Res.* 2011;17(20):6448-6458.

Chen F, Teachey DT, Pequignot E, et al. Measuring IL-6 and sIL-6R in serum from patients treated with tocilizumab and/or siltuximab following CAR T cell therapy. *J Immunol Methods*. 2016;434:1-8.

Cheson BD, Pfistner B, Juweid ME, et al. Revised response criteria for malignant lymphoma. *J Clin Oncol*. 2007;25(5):579-586.

Cheson B, Fisher R, Barrington S, et al. Recommendations for Initial Evaluation, Staging, and Response Assessment of Hodgkin and Non-Hodgkin Lymphoma: The Lugano Classification. *J Clin Oncol.* 2014;32(27):3059-3067.

Davila M, Riviere I, Wang X, et al. Efficacy and Toxicity Management of 1928z CAR T Cell Therapy in B Cell Acute Lymphoblastic Leukemia. *Sci Transl Med* Feb2014;6(224):224ra25.

Delves PJ, Martin SJ, Burton DR, Roitt IM. *Roitt's essential immunology.* John Wiley & Sons; 2017.

Dorman SE, Holland SM. Interferon-γ and interleukin-12 pathway defects and human disease. *Cytokine & growth factor reviews.* 2000;11(4):321-333. doi:

Dunleavy K, Pittaluga S, Maeda LS, et al. Dose-adjusted EPOCH-rituximab therapy in primary mediastinal B-cell lymphoma. *New England Journal of Medicine.* 2013;368(15):1408-1416. doi:

Ferreria R, Vastert S, Abinun M, et al. Hemophagocytosis during fludarabine-based SCT for systemic juvenile idiopathic arthritis. *Bone Marrow Transplantation* 2006;38:249-251.

Flammiger A, Fiedler W, Bacher U, Bokemeyer C, Schneider M, Binder M. Critical imbalance of TNF-α and soluble TNF receptor 1 in a patient with macrophage activation syndrome: potential implications for diagnostics and treatment. *Acta Haematol*. 2012;128(2):69-72. doi:10.1159/000338179

Flowers CR, Sinha R, Vose JM. Improving outcomes for patients with diffuse large B-cell lymphoma. *CA Cancer J Clin*. 2010;60(6):393-408. doi:10.3322/caac.20087

Dunleavy K, Pittaluga S, Maeda LS, et al. Dose-adjusted EPOCH-rituximab therapy in primary mediastinal B-cell lymphoma. *New England Journal of Medicine.* 2013;368(15):1408-1416. doi:

Gabay C, Lamacchia C, Palmer G. IL-1 pathways in inflammation and human diseases. *Nat Rev Rheumatol*. 2010;6(4):232-241. doi:10.1038/nrrheum.2010.4

Gattinoni L, Finkelstein SE, Klebanoff CA, et al. Removal of homeostatic cytokine sinks by lymphodepletion enhances the efficacy of adoptively transferred tumor-specific CD8+ T cells. *J Exp Med*. 2005;202(7):907-912. doi:10.1084/jem.20050732

Guidance for Sponsors, Clinical Investigators, and IRBs Data Retention When Subjects Withdraw from FDA-Regulated Clinical Trials, 2008 (http://www.fda.gov/Drugs/default.htm) Published October 2008, Accessed June 27, 2014.

Dunleavy K, Pittaluga S, Maeda LS, et al. Dose-adjusted EPOCH-rituximab therapy in primary mediastinal B-cell lymphoma. *New England Journal of Medicine.* 2013;368(15):1408-1416. doi:

Jordan MB, Allen CE, Weitzman S, Filipovich AH, McClain KL. How I treat hemophagocytic lymphohistiocytosis. *Blood, The Journal of the American Society of Hematology*. 2011;118(15):4041-4052. doi:

Josting A, Rueffer U, Franklin J, Sieber M, Diehl V, Engert A. Prognostic factors and treatment outcome in primary progressive Hodgkin lymphoma: a report from the German Hodgkin Lymphoma Study Group. *Blood*. 2000;96(4):1280-1286.

Kershaw MH, Westwood JA, Darcy PK. Gene-engineered T cells for cancer therapy. *Nat Rev Cancer*. 2013;13(8):525-541. doi:10.1038/nrc3565

Khouri IF, Keating M, Körbling M, et al. Transplant-lite: induction of graft-versus-malignancy using fludarabine-based nonablative chemotherapy and allogeneic blood progenitor-cell transplantation as treatment for lymphoid malignancies. *J Clin Oncol*. 1998;16(8):2817-2824. doi:10.1200/JCO.1998.16.8.2817

Kimura A, Kishimoto T. IL-6: regulator of Treg/Th17 balance. *Eur J Immunol*. 2010;40(7):1830-1835. doi:10.1002/eji.201040391

Klebanoff CA, Khong HT, Antony PA, Palmer DC, Restifo NP. Sinks, suppressors and antigen presenters: how lymphodepletion enhances T cell-mediated tumor immunotherapy [published correction appears in Trends Immunol. 2005 Jun;26(6):298]. *Trends Immunol*. 2005;26(2):111-117. doi:10.1016/j.it.2004.12.003

Klein JP, Moeschberger ML. *Survival analysis: techniques for censored and truncated data.* Vol 1230: Springer; 2003.

Kochenderfer JN, Feldman SA, Zhao Y, et al. Construction and preclinical evaluation of an anti-CD19 chimeric antigen receptor. *J Immunother*. 2009;32(7):689-702. doi:10.1097/CJI.0b013e3181ac6138

Kochenderfer JN, Yu Z, Frasheri D, Restifo NP, Rosenberg SA. Adoptive transfer of syngeneic T cells transduced with a chimeric antigen receptor that recognizes murine CD19 can eradicate lymphoma and normal B cells. *Blood*. 2010;116(19):3875-3886. doi:10.1182/blood-2010-01-265041

Kochenderfer JN, Dudley ME, Feldman SA, et al. B-cell depletion and remissions of malignancy along with cytokine-associated toxicity in a clinical trial of anti-CD19 chimeric-antigen-receptor-transduced T cells. *Blood*. 2012;119(12):2709-2720. doi:10.1182/blood-2011-10-384388

Kochenderfer JN, Dudley ME, Kassim SH, et al. Effective treatment of chemotherapy-refractory diffuse large B-cell lymphoma with autologous T cells genetically-engineered to express an anti-CD19 chimeric

antigen receptor. *Blood.* 2013;122(21):168. doi:

Kochenderfer JN, Dudley ME, Kassim SH, et al. Chemotherapy-refractory diffuse large B-cell lymphoma and indolent B-cell malignancies can be effectively treated with autologous T cells expressing an anti-CD19 chimeric antigen receptor. *Journal of Clinical Oncology.* 2015;33(6):540. doi:

Kowolik C, Topp M, Gonzalez S, et al. CD28 costimulation provided through a specific chimeric antigen receptor enhances in vivo persistence and antitumor efficacy of adoptively transferred T cells. *Cancer Res* 2006;66(22):10995-11004.

Kuruvilla J, Pintilie M, Tsang R, Nagy T, Keating A, Crump M. Salvage chemotherapy and autologous stem cell transplantation are inferior for relapsed or refractory primary mediastinal large B-cell lymphoma compared with diffuse large B-cell lymphoma. *Leuk Lymphoma*. 2008;49(7):1329-1336. doi:10.1080/10428190802108870

Lackner H, Urban C, Sovinz P, Benesch M, Moser A, Schwinger W. Hemophagocytic lymphohistiocytosis as severe adverse event of antineoplastic treatment in children. *Haematologica*. 2008;93(2):291-294. doi:10.3324/haematol.11704

La Rosée P. Treatment of hemophagocytic lymphohistiocytosis in adults. *Hematology Am Soc Hematol Educ Program*. 2015;2015:190-196. doi:10.1182/asheducation-2015.1.190

Lee DW, Gardner R, Porter DL, et al. Current concepts in the diagnosis and management of cytokine release syndrome [published correction appears in Blood. 2015 Aug 20;126(8):1048. Dosage error in article text] [published correction appears in Blood. 2016 Sep 15;128(11):1533]. *Blood*. 2014;124(2):188-195. doi:10.1182/blood-2014-05-552729

Lee D, Kochenderfer J, Stetler-Stevenson M, et al. T cells expressing chimeric antigen receptorsfor acute lymphoblastic leukaemia in children and young adults: a phase 1 dose-escalation trial. *Lancet* 2015;185(9967):517-28.

Li P, Du Q, Cao Z, et al. Interferon-γ induces autophagy with growth inhibition and cell death in human

hepatocellular carcinoma (HCC) cells through interferon-regulatory factor-1 (IRF-1). *Cancer Lett*. 2012;314(2):213-222. doi:10.1016/j.canlet.2011.09.031

Key NS, Khorana AA, Kuderer NM, et al. Venous Thromboembolism Prophylaxis and Treatment in Patients With Cancer: ASCO Clinical Practice Guideline Update. *J Clin Oncol*. 2020;38(5):496-520. doi:10.1200/JCO.19.01461

Lasek W, Zagożdżon R, Jakobisiak M. Interleukin 12: still a promising candidate for tumor immunotherapy. *Cancer Immunol Immunother*. 2014;63(5):419-435. doi:10.1007/s00262-014-1523-1

Ma CS, Deenick EK, Batten M, Tangye SG. The origins, function, and regulation of T follicular helper cells. *J Exp Med*. 2012;209(7):1241-1253. doi:10.1084/jem.20120994

Magee MS, Kraft CL, Abraham TS, et al. GUCY2C-directed CAR-T cells oppose colorectal cancer metastases without autoimmunity. *Oncoimmunology*. 2016;5(10):e1227897. Published 2016 Sep 2. doi:10.1080/2162402X.2016.1227897

Kochenderfer JN, Dudley ME, Kassim SH, et al. Chemotherapy-refractory diffuse large B-cell lymphoma and indolent B-cell malignancies can be effectively treated with autologous T cells expressing an anti-CD19 chimeric antigen receptor. *J Clin Oncol*. 2015;33(6):540-549. doi:10.1200/JCO.2014.56.2025

Marszalowicz GP, Snook AE, Magee MS, Merlino D, Berman-Booty LD, Waldman SA. GUCY2C lysosomotropic endocytosis delivers immunotoxin therapy to metastatic colorectal cancer. *Oncotarget*. 2014;5(19):9460-9471. doi:10.18632/oncotarget.2455

Matasar MJ, Czuczman MS, Rodriguez MA, et al. Ofatumumab in combination with ICE or DHAP chemotherapy in relapsed or refractory intermediate grade B-cell lymphoma. *Blood*. 2013;122(4):499-506. doi:10.1182/blood-2012-12-472027

Matsuzawa T, Fujiwara E, Washi Y. Autophagy activation by interferon-γ via the p38 mitogen-activated protein kinase signalling pathway is involved in macrophage bactericidal activity. *Immunology*. 2014;141(1):61-69. doi:10.1111/imm.12168

Matsuzawa T, Kim BH, Shenoy AR, Kamitani S, Miyake M, Macmicking JD. IFN-γ elicits macrophage autophagy via the p38 MAPK signaling pathway. *J Immunol*. 2012;189(2):813-818. doi:10.4049/jimmunol.1102041

Maude SL, Teachey DT, Porter DL, Grupp SA. CD19-targeted chimeric antigen receptor T-cell therapy for acute lymphoblastic leukemia. *Blood*. 2015;125(26):4017-4023. doi:10.1182/blood-2014-12-580068

Maude SL, Barrett D, Teachey DT, Grupp SA. Managing cytokine release syndrome associated with novel T cell-engaging therapies. *Cancer J*. 2014;20(2):119-122. doi:10.1097/PPO.0000000000000035

Moskowitz CH, Bertino JR, Glassman JR, et al. Ifosfamide, carboplatin, and etoposide: a highly effective cytoreduction and peripheral-blood progenitor-cell mobilization regimen for transplant-eligible patients with non-Hodgkin's lymphoma. *J Clin Oncol*. 1999;17(12):3776-3785. doi:10.1200/JCO.1999.17.12.3776

Moyé LA, Deswal A. Trials within trials: confirmatory subgroup analyses in controlled clinical experiments. *Control Clin Trials*. 2001;22(6):605-619. doi:10.1016/s0197-2456(01)00180-5

Nagle SJ, Woo K, Schuster SJ, et al. Outcomes of patients with relapsed/refractory diffuse large B-cell lymphoma with progression of lymphoma after autologous stem cell transplantation in the rituximab era. *Am J Hematol*. 2013;88(10):890-894. doi:10.1002/ajh.23524

National Comprehensive Cancer Network. NCCN Clinical Practice Guidelines in Oncology (NCCN, 2015).

Nicholson IC, Lenton KA, Little DJ, et al. Construction and characterisation of a functional CD19 specific single chain Fv fragment for immunotherapy of B lineage leukaemia and lymphoma. *Mol Immunol*. 1997;34(16-17):1157-1165. doi:10.1016/s0161-5890(97)00144-2

O'Brien SM, Kantarjian HM, Cortes J, et al. Results of the fludarabine and cyclophosphamide combination regimen in chronic lymphocytic leukemia. *J Clin Oncol*. 2001;19(5):1414-1420. doi:10.1200/JCO.2001.19.5.1414

Philip T, Guglielmi C, Hagenbeek A, et al. Autologous bone marrow transplantation as compared with salvage chemotherapy in relapses of chemotherapy-sensitive non-Hodgkin's lymphoma. *N Engl J Med*. 1995;333(23):1540-1545. doi:10.1056/NEJM199512073332305

Porter DL, Hwang WT, Frey NV, Lacey SF, Shaw PA, Loren AW, Bagg A, Marcucci KT, Shen A, Gonzalez V, Ambrose D, Grupp SA, Chew A, Zheng Z, Milone MC, Levine BL, Melenhorst JJ, June CH. Chimeric antigen receptor T cells persist and induce sustained remissions in relapsed refractory chronic lymphocytic leukemia. Sci Transl Med. 2015 Sep 2;7(303):303ra139. doi: 10.1126/scitranslmed.aac5415. PMID: 26333935; PMCID: PMC5909068.

Prahalad S, Bove KE, Dickens D, Lovell DJ, Grom AA. Etanercept in the treatment of macrophage activation syndrome [published correction appears in J Rheumatol 2001 Oct;28(10):2370]. *J Rheumatol*. 2001;28(9):2120-2124.

Putter H, Fiocco M, Geskus RB. Tutorial in biostatistics: competing risks and multi-state models. *Stat Med*. 2007;26(11):2389-2430. doi:10.1002/sim.2712

Savage KJ, Al-Rajhi N, Voss N, et al. Favorable outcome of primary mediastinal large B-cell lymphoma in a single institution: the British Columbia experience. *Ann Oncol*. 2006;17(1):123-130. doi:10.1093/annonc/mdj030

Schroder K, Hertzog PJ, Ravasi T, Hume DA. Interferon-gamma: an overview of signals, mechanisms and functions. *J Leukoc Biol*. 2004;75(2):163-189. doi:10.1189/jlb.0603252

Sehn LH, Antin JH, Shulman LN, et al. Primary diffuse large B-cell lymphoma of the mediastinum: outcome following high-dose chemotherapy and autologous hematopoietic cell transplantation. *Blood*. 1998;91(2):717-723.

Seshadri T, Kuruvilla J, Crump M, Keating A. Salvage therapy for relapsed/refractory diffuse large B cell lymphoma. *Biol Blood Marrow Transplant*. 2008;14(3):259-267. doi:10.1016/j.bbmt.2007.11.013

Snook AE, Li P, Stafford BJ, Faul EJ, Huang L, Birbe RC, Bombonati A, Schulz S, Schnell MJ, Eisenlohr LC, Waldman SA. Lineage-specific T-cell responses to cancer mucosa antigen oppose systemic metastases without mucosal inflammatory disease. Cancer Res. 2009 Apr 15;69(8):3537-44. doi: 10.1158/0008-5472.CAN-08-3386. Epub 2009 Apr 7. PMID: 19351847; PMCID: PMC2707278.

Snook AE, Magee MS, Schulz S, Waldman SA. Selective antigen-specific CD4(+) T-cell, but not CD8(+) T- or B-cell, tolerance corrupts cancer immunotherapy. *Eur J Immunol*. 2014;44(7):1956-1966. doi:10.1002/eji.201444539

Surveillance, Epidemiology, and End Results (SEER) Program Populations (1969-2012) (www.seer.cancer.gov/popdata), National Cancer Institute, DCCPS, Surveillance Research Program, Surveillance Systems Branch, released March 2014.

Song Y, Chi GY. A method for testing a prespecified subgroup in clinical trials. *Stat Med*. 2007;26(19):3535-3549. doi:10.1002/sim.2825

Teachey DT, Rheingold SR, Maude SL, et al. Cytokine release syndrome after blinatumomab treatment related to abnormal macrophage activation and ameliorated with cytokine-directed therapy. *Blood*. 2013;121(26):5154-5157. doi:10.1182/blood-2013-02-485623

Telio D, Fernandes K, Ma C, et al. Salvage chemotherapy and autologous stem cell transplant in primary refractory diffuse large B-cell lymphoma: outcomes and prognostic factors. *Leuk Lymphoma*. 2012;53(5):836-841. doi:10.3109/10428194.2011.643404

Voest EE, Kenyon BM, O'Reilly MS, Truitt G, D'Amato RJ, Folkman J. Inhibition of angiogenesis in vivo by interleukin 12. *J Natl Cancer Inst*. 1995;87(8):581-586. doi:10.1093/jnci/87.8.581

Wang SJ, O'Neill RT, Hung HM. Approaches to evaluation of treatment effect in randomized clinical trials with genomic subset. *Pharm Stat*. 2007;6(3):227-244. doi:10.1002/pst.300

Appendix

Mini Mental State Examination (MMSE)

Department:_Bed No.: ___Name: _Gender: _Age:

Inpatient No.:_____Education background:_______ Date: Total score:

Items			Result	Score
**I Orientation (10 points)**	Week			0 1
	Date			0 1
	Month			0 1
	Season			0 1
	Year			0 1
	Province and city			0 1
	District and country			0 1
	Street or townships			0 1
	Location			0 1
	Floor			0 1
**II Memory (3 points)**	Ball			0 1
	Flag			0 1
	Tree			0 1
**III Attention and computing ability (5 points)**	100-7			0 1
	-7			0 1
	-7			0 1
	-7			0 1
	-7			0 1
**IV Recall (3 points)**	Ball			0 1
	Flag			0 1
	Tree			0 1
**V Language ability (9 points)**	Naming ability			0 1
				0 1
	Retelling ability			0 1
	Three-step command			0 1
				0 1
				0 1
	Reading ability			0 1
	Writing ability			0 1
	Drawing ability	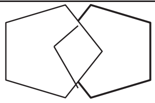		0 1
**Total**				

Doctor’s signature:

Instructions on the procedure

I. Orientation (highest score: 10 points)

1. Ask about the date first, and then ask about other questions, such as "Can you tell me what season it is?" One point is given for each correct answer.

2. Please raise the questions in turn, "Can you tell me where we are?" (district or county? street? location? or floor?). One point is given for each correct answer.

II．Memory (highest score: 3 points)

Tell the subject that you will ask a few questions to check his or her memory and then clearly and slowly mention three unrelated things (such as a ball, a flag, and a tree, with the interval at approximately 1 second). After you have mentioned all three things, ask the subject to repeat them. The subjects' scores depend on the answers they repeat for the first time. (One point for each correct answer, and 3 points at most). If they do not remember all the things completely, you will repeat again, but the repetition shall be no more than five times. If they still fail to remember all three things after your repetition five times, then it is meaningless to conduct the rest of the recall ability test. (Please skip Part IV "Recall" Test).

III．Attention and computing ability (highest score: 5 points)

Ask the patient to subtract 7 from 100, and then subtract 7 again, and so on, 5 consecutive times (i.e., 93, 86, 79, 72, and 65). One point is given for each correct answer, and 1 point is given for the correct answer of the next question if the answer of the previous one was wrong.

IV．Recall (highest score: 3 points)

If the subject remembers all three things in the last test, ask him or her to repeat them now. One point is given for each correct answer. The highest possible score is 3 points.

V．Language ability (highest score: 9 points)

1. Naming ability (0-2 points): show a watch card to the subject and ask him or her to say what it is. After that, show a pencil to him or her and ask him or her the same question.

2. Retelling ability (0-1 points): ask the subject to pay attention to what you say and ask him or her to repeat it once (only once). This sentence you will say is "forty-four stone lions". Only the subject who articulates correctly and clearly will be awarded 1 point.

3. Three-step command (0-3 points): give the subject a piece of blank paper and ask him or her to follow your command. Note not to repeat or give any demonstration. The subject will be scored only if he or she completes the steps of the command in the correct order. One point is given for each correct step.

4. Reading ability (0-1 points): take out a card stating "Close Your Eyes" and show it to the subject. Ask the subject to read it and do it as requested. The subject will be scored only if he or she does close his or her eyes.

5. Writing ability (0-1 points): give the subject a piece of white paper and ask him or her to write a complete sentence spontaneously. The sentence shall have a subject, a verb and a meaning. Note that you are not allowed to give any hints. Mistakes in grammars and punctuations may be ignored.
6. Drawing ability (0-1 points): draw two overlapping pentagons on a piece of white paper and ask the subject to draw and copy them accurately. Scoring criteria: five corners and five sides of the pentagons shall be clearly drawn. In addition, the overlapping part of the two pentagons forms a diamond. The crooked lines and the rotation of graphics may be ignored.
Dementia classification criteria: illiteracy level < 17 points, primary school level < 20 points, and secondary school level (including special school) < 24 points

Appendix

Interpretation of abnormal glycosylated GUCY2C in tumor tissues

1. The expression of abnormal glycosylated GUCY2C in tumor tissues will be evaluated using immunohistochemistry (IHC). The antibody used to label abnormal GUCY2C is provided by our company.
2. Immunohistochemistry testing requires the use of recent tumor tissues (collected by puncture/operation), embedded tissue blocks, tumor sections, etc. The samples are required to be complete morphologically with clear structures and be different from normal tissues.
3. Based on the results of immunohistochemical staining intensity, abnormal GUCY2C expression is divided into three intensities: weak, medium and strong. In addition, the ratio of abnormal GUCY2C to tumor cells is scored according to the staining results, i.e., 0% = 0 points, 1-10% = 1 point, 11-50% = 2 points, 51-80% = 3 points, and 81-100% = 4 points. Based on the results of the test and Remmele scoring system, a comprehensive immune response score ≥ 3 points is initially considered the basic inclusion criteria.

IRS scoring formula: SI X Percentage of positive cells= Score

Immunoreactive Score accord. To Remmele und Stegner(IRS)			
SI x	Percentage of positive cells =	Score	Classification
0	0= Negative	0-1	0
1	1=<10% positive cells	2-3	1
2	2= 10%-50% positive cells	4-8	2
3	3= 51%-80% positive cells	9-12	3
	4= >80% positive cells		

SI (Staining Intensities)

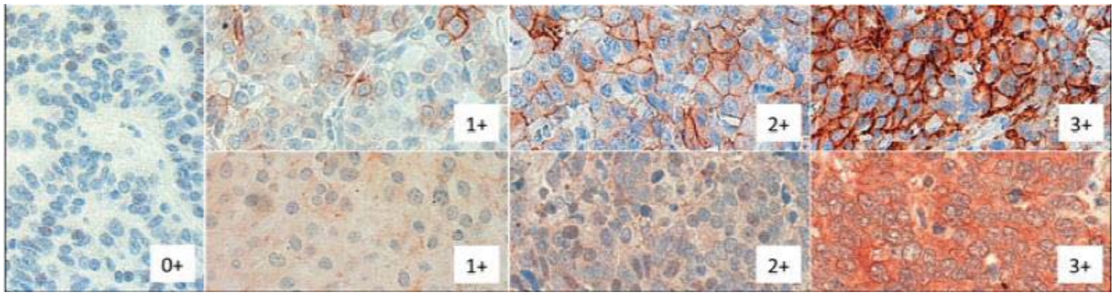

Appendix

XX Hospital- patient Zhang San with solid tumor- sampling plan

	Blood collected on	0D	2D	4D	6D	8D	10D	12D	14D	16D	18D
Date	2019/7/25	2019/8/3	2019/8/5	2019/8/7	2019/8/9	2019/8/11	2019/8/13	2019/8/15	2019/8/17	2019/8/19	2019/8/21
Day	Thursday	Saturday	Monday	Wednesday	Friday	Sunday	Tuesday	Thursday	Saturday	Monday	Wednesday
Sample type	PB	PB	PB	PB	PB	PB	PB	PB	PB	PB	PB
EDTA anticoagulant tube	2 tubes: (2~3 ml)	2 tubes before e retransfusion	2 tubes (2~3 ml)	2 tubes (2~3 ml)	2 tubes (2~3 ml)	2 tubes (2~3 ml)	2 tubes (2~3 ml)	2 tubes (2~3 ml)	2 tubes (2~3 ml)	(2~3 ml)	2 tubes: (2~3 ml)
Total	2 tubes	2 tubes	2 tubes	2 tubes	2 tubes	2 tubes	2 tubes	2 tubes	2 tubes	2 tubes:	2 tubes:
	21D	24D	27D	30D	37D	45D	52D	60D	M3	M6	M9
Date	2019/8/24	2019/8/27	2019/8/30	2019/9/2	2019/9/9	2019/9/17	2019/9/24	2019/10/2	2019/11/1	2020/2/2	2020/5/2
Day	Saturday	Tuesday	Friday	Monday	Monday	Tuesday	Tuesday	Wednesday	Friday	Sunday	Saturday
Sample type	PB	PB	PB	PB	PB	PB	PB	PB	PB	PB	PB
EDTA anticoagulant tube	2 tubes (2~3 ml)	2 tubes (2~3 ml)	2 tubes (2~3 ml)	2 tubes (2~3 ml)	2 tubes (2~3 ml)	2 tubes (2~3 ml)	2 tubes (2~3 ml)	2 tubes (2~3 ml)	2 tubes (2~3 ml)	2 tubes (2~3 ml)	2 tubes: (2~3 ml)
Total	2 tubes	2 tubes	2 tubes	2 tubes	2 tubes	2 tubes	2 tubes	2 tubes	2 tubes	2 tubes:	2 tubes:
	M12	M15	M18	M24	M30	M36	M42	M48	M54	M60	Note: 1. Tissue samples will be collected depending on the actual situation. 2. Sample collection shall be completed before 12 O'clock every day (except for special circumstances). 3. The company will arrange to receive the samples. 4. Temporary samples shall be stored in a 4 °C refrigerator. 5. If a patient develops a fever over 38.3 °C within the first three months after the retransfusion, 2 EDTA anticoagulant tubes of blood samples shall be collected, and the patient shall cooperate with blood routine test on the same day
Date	2020/8/2	2020/10/31	2021/1/31	2021/8/2	2022/1/31	2022/8/2	2023/1/31	2023/8/2	2024/1/31	2024/8/1	
Day	Sunday	Saturday	Sunday	Monday	Monday	Tuesday	Tuesday	Wednesday	Wednesday	Thursday	
Sample type	PB	PB	PB	PB	PB	PB	PB	PB	PB	PB	
EDTA anticoagulant tube	2 tubes: (2~3 ml)	2 tubes: (2~3 ml)	2 tubes: (2~3 ml)	2 tubes: (2~3 ml)	2 tubes: (2~3 ml)	2 tubes: (2~3 ml)	2 tubes: (2~3 ml)	2 tubes: (2~3 ml)	2 tubes: (2~3 ml)	2 tubes: (2~3 ml)	
Total	2 tubes:	2 tubes:	2 tubes:	2 tubes:	2 tubes:	2 tubes:	2 tubes:	2 tubes:	2 tubes:	2 tubes:	

											of blood collection.

Notes:

1. According to the patient's clinical conditions and the cytokine test results, additional blood samples may be requested to be collected from the patient on the day after the copy numbers of cytokines or CAR cells suddenly increase;
