# Supplementary material for: Chimeric Antigen Receptor T Cells Targeting CD19 and GCC in Metastatic Colorectal Cancer: A Nonrandomized Clinical Trial
Source: JAMA Oncol. 2024 Sep 19;10(11):1532–6. doi: 10.1001/jamaoncol.2024.3891 (PMC11413756; doi:10.1001/jamaoncol.2024.3891)
Supplement: Supplement 3. — Data sharing statement [file jamaoncol-e243891-s003.pdf]

Data Sharing Statement

Chen. Chimeric Antigen Receptor T Cells Targeting CD19 and GCC in Metastatic Colorectal Cancer. *JAMA Oncol*. Published September 19, 2024. doi:10.1001/jamaoncol.2024.3891

Data
Data available: Yes
Data types: Deidentified participant data
How to access data: cuijw@jlu.edu.cn
When available: With publication

Supporting Documents
Document types: None

Additional Information
Who can access the data: Jiuwei Cui
Types of analyses: for a specified purpose
Mechanisms of data availability: with investigator support
